# Gastroesophageal reflux disease and osteoporosis: A bidirectional Mendelian randomization study

**DOI:** 10.1097/MD.0000000000042083

**Published:** 2025-04-04

**Authors:** Qinghua Yang, Longao Huang, Hongyuan Xu, Junfei Feng, Dun Liu, Shengwang Wei, Hua Jiang

**Affiliations:** a Department of Spine Surgery, The First Affiliated Hospital of Guangxi Medical University, Nanning, China; b Department of Orthopedics, The Fourth Affiliated Hospital of Guangxi Medical University/Liuzhou Worker’s Hospital, Liuzhou, China.

**Keywords:** bone mineral density, gastroesophageal reflux disease, GWAS data, Mendelian randomization, osteoporosis

## Abstract

In observational studies, associations between osteoporosis (OP) and gastroesophageal reflux disease (GERD) have been found. We conducted a 2-way, 2-sample Mendelian randomization (MR) analysis to determine whether these associations have a causal relationship. Data on GERD at the summary-level were sourced from extensive genome-wide association studies encompassing 129,080 cases and 473,524 control subjects. Bone mineral density (BMD) served as the phenotypic indicator for OP. BMD metrics were compiled from a cohort of 537,750 individuals, encompassing total body BMD (TB-BMD) and stratified TB-BMD across age groups, along with BMD measurements at 4 anatomical locations: lumbar spine, femoral neck, heel, and ultra-distal forearm. Multiple MR approaches, such as the inverse-variance weighted (IVW) method, MR-Egger regression, and the MR-PRESSO test, were employed, among which findings obtained by IVW method were designated as the primary outcomes. For quality assurance, sensitivity analyses were conducted using the MR-Egger intercept, Cochran *Q*, and leave-one-out test. There were no significant causal links between genetic inclination towards GERD and reduced BMD levels. Nonetheless, the genetic evidence suggests a causal link between higher BMD levels and lower incidence of GERD [TB-BMD: OR = 0.941, 95% confidence intervals (CI) = 0.910–0.972, *P* < .001; TB-BMD-1: OR = 0.919, 95% CI = 0.885–0.954, *P* < .001; TB-BMD-3: OR = 0.945, 95% CI = 0.915–0.977, *P* = .001; TB-BMD-4: OR = 0.926, 95% CI = 0.896–0.957, *P* < .001]. Sensitivity analyses corroborate our findings. The MR analysis indicates no significant causal link between genetic inclination towards GERD and OP or reduced BMD within the European demographic. In addition, the study suggests that lower BMD or OP, as predicted by genetics, may contribute to the development of GERD.

## 1. Introduction

Osteoporosis (OP) is a slowly progressing systemic metabolic bone disease, where the primary pathological process is characterized by an imbalance between bone formation and bone resorption during bone remodeling.^[1]^ This imbalance can lead to bone loss, bone microstructure destruction, and increased bone fragility, making patients prone to low-energy fractures.^[1]^ Notably, OP affects not only the skeleton but also the internal organs.^[2,3]^ For instance, OP-related kyphosis may lead to end-organ dysfunction, such as the association between thoracic hyperkyphosis and dysphagia.^[3]^ Currently, the gold standard for diagnosing OP is through bone mineral density (BMD) measurements of the lumbar spine, proximal femur, and distal forearm, using dual-energy X-ray absorptiometry (DXA).^[4]^

Gastroesophageal reflux disease (GERD) is a common digestive disorder characterized by the abnormal reflux of gastric contents into the esophagus, which leads to damage of the esophageal mucosa or symptoms associated with reflux.^[5]^ GERD affects approximately 13% of the worldwide population, with about 20% of the adult population in Western countries being impacted.^[6,7]^ GERD significantly reduces the patients’ quality of life and increases the risk of esophageal complications such as esophagitis, esophageal strictures, Barrett esophagus, and esophageal adenocarcinoma.^[6,7]^ Studies have shown that OP and GERD are related to each other. A gastrointestinal endoscopy study conducted by Furukawa et al^[8]^ indicated that older women had a higher prevalence of hiatus hernia, possibly explained by the increased prevalence of osteoporotic kyphosis in this population. Shiraki et al^[9]^ performed an upper gastrointestinal barium study and proved an association between kyphosis and gastric acid reflux in postmenopausal women. Proton pump inhibitors (PPIs) are the most effective agents in treating GERD.^[10]^ A consensus regarding the relationship between long-term use of PPIs and OP risk has not yet been established. Research has indicated that PPIs usage could be associated with a marginal reduction in BMD, potentially elevating the likelihood of OP and fractures which are not caused by trauma.^[11–14]^ Nevertheless, a previous study suggested that the use of PPIs might slightly increase BMD by reducing bone turnover.^[15]^ In addition, other studies have found no association between PPIs and OP or bone loss.^[15]^

However, present studies have limitations such as imprecise or unmeasured confounders, various interpretations of the research findings, and insufficient statistical robustness due to small sample size. Employing Mendelian randomization (MR) can overcome those limitations. MR uses genetic variants as instrumental variables (IVs) to evaluate whether the relationships between exposure factors and observed outcomes are causal.^[16]^ The advantage of MR lies in the properties of randomly assigned genetic variations unaffected by self-selected lifestyle and environmental factors, thereby minimizing residual confounding.^[16]^ Moreover, it can address the issue of reverse causation because genetic variants cannot be modified by disease status.^[16]^ Our team adopted a bidirectional MR approach to explore the possible causal links between GERD and OP.

## 2. Materials and methods

### 2.1. Study design

**Figure 1.**
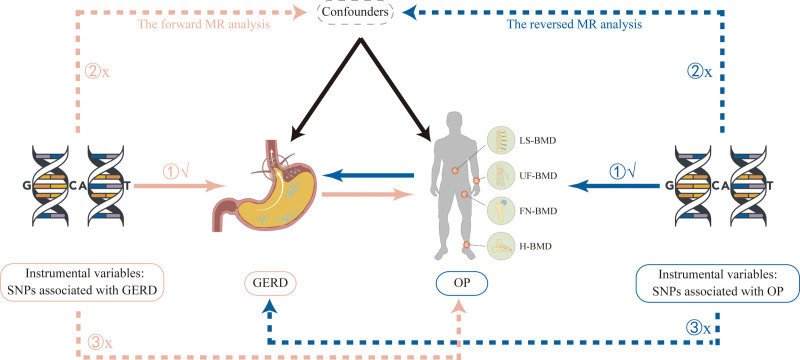
The framework of 2-sample bidirectional MR analysis. Three core assumptions were as follows: (A) relevance assumption; (B) independence assumption; (C) exclusion restriction. FN-BMD = Femoral neck bone mineral density, GERD = gastroesophageal reflux disease, H-BMD = heel bone mineral density, LS-BMD = lumbar spine bone mineral density, MR = Mendelian randomization, OP = osteoporosis, SNP = single nucleotide polymorphism, UF-BMD = ultra-distal forearm bone mineral density.

The validity of the MR approach is contingent upon 3 fundamental assumptions: genetic variation is associated with exposure; genetic variation is not linked to any confounders that influence exposure or outcome; genetic variation is associated with the outcome solely through the exposure. The bidirectional MR analysis proceeded in 2 stages: in the first step, GERD was examined as the exposure, and OP-related traits as the outcome. Subsequently, the roles were reversed. Figure 1 provides a schematic representation of the 3 assumptions and the study design. This study was reported in accordance with the strengthening the reporting of observational studies in epidemiology using Mendelian randomization (STROBE-MR) checklist (Table S1, Supplemental Digital Content, https://links.lww.com/MD/O636).^[17]^

### 2.2. Data sources

The GERD genome-wide association study (GWAS) dataset was sourced from Ong et al,^[18]^ encompassing 129,080 cases and 473,524 controls. The GWAS summary-level data on GERD was retrieved from the GWAS Catalog. In the study by Ong et al,^[18]^ controls were defined as individuals without any history or current occurrence of upper digestive system disorders. GERD cases were defined based on a combination of self-reported GERD symptoms such as heartburn, the use of GERD medication, and medical records based on ICD-10 codes. Every participant was obligated to fill out a consent form, and the study by Ong et al^[18]^ had been approved by the QIMR Berghofer Human Research Ethics Committee under project ID 3501.

To examine age-specific and site-specific BMD, we used summary-level data of BMD from different age stages and skeletal sites. For the overall BMD, the total body BMD (TB-BMD) was assessed by DXA. The TB-BMD dataset was obtained from the genetic factors for osteoporosis consortium (GEFOS) meta-analysis,^[19]^ encompassing 66,628 participants. Among the participants, 86% were identified as European, 12% as mixed Oceanian, and 2% as African American. This dataset covered 5 different age stages: ≤15 years (N = 11,807), 15–30 years (N = 4180), 30–45 years (N = 10,062), 45–60 years (N = 18,805), ≥60 years (N = 22,504).^[19]^ The GWAS datasets for heel BMD (H-BMD)^[20]^ and ultra-distal forearm BMD (UF-BMD)^[21]^ included 426,824 and 21,907 individuals, respectively, all of European descent. H-BMD was estimated by quantitative ultrasound, and UF-BMD was measured by single-energy X-ray absorptiometry. The data for femoral neck BMD (FN-BMD) and lumbar spine BMD (LS-BMD) originated from another GEFOS study,^[22]^ comprising 32,735 and 28,498 participants, the majority of whom are White British. FN-BMD and LS-BMD were assessed by DXA. Relevant ethics committees had approved all studies contributing data to these analyses.

All the summary statistics for GERD data and BMD data can be downloaded from the integrative epidemiology unit open GWAS database (https://gwas.mrcieu.ac.uk/). Detailed information regarding the datasets employed in our research has been summarized in Table 1.

**Table 1:** Details of the genome-wide association studies and datasets used in this study.

Exposure or outcome	Abbreviations	Sample size	Data source	Ancestry	PMID
Total body bone mineral density	TB-BMD	56,284	GEFOS	European	29304378
Total body bone mineral density (age over 60)	TB-BMD-1	22,504	GEFOS	Mixed	29304378
Total body bone mineral density (age 45–60)	TB-BMD-2	18,805	GEFOS	European	29304378
Total body bone mineral density (age 30–45)	TB-BMD-3	10,062	GEFOS	Mixed	29304378
Total body bone mineral density (age 15–30)	TB-BMD-4	4180	GEFOS	Mixed	29304378
Total body bone mineral density (age 0–15)	TB-BMD-5	11,807	GEFOS	Mixed	29304378
Heel bone mineral density	H-BMD	426,824	UK Biobank	European	30598549
Ultra-distal forearm bone mineral density	UF-BMD	21,907	GEFOS	European	33097703
Femoral neck bone mineral density	FN-BMD	32,735	GEFOS	Mixed	26367794
Lumbar spine bone mineral density	LS-BMD	28,498	GEFOS	Mixed	26367794
Gastroesophageal reflux disease	GERD	602,604	GWAS meta-analysis	European	34187846

FN-BMD = Femoral neck bone mineral density, GEFOS = Genetic Factors for Osteoporosis Consortium Website, GWAS = genome-Wide Association Studies, H-BMD = heel bone mineral density, LS-BMD = lumbar spine bone mineral density, PMID = pubmed unique identifier, TB-BMD = total body bone mineral density, TB-BMD-1 = total body bone mineral density (age over 60), TB-BMD-2 = total body bone mineral density (age 45–60), TB-BMD-3 = total body bone mineral density (age 30–45), TB-BMD-4 = total body bone mineral density (age 15–30), TB-BMD-5 = total body bone mineral density (age 0–15), UF-BMD = ultra-distal forearm bone mineral density.

### 2.3. Genetic IVs selection

First, genome-wide divergent SNPs with *P* < 5 × 10^–8^ and linkage disequilibrium of *r*^2^ < 0.001 and a genetic distance of 10,000 kb were selected as IVs. For TB-BMD-4, a more lenient significance threshold of *P* < 5 × 10^−6^ was adopted because only a single SNP was identified at the more stringent level of *P* < 5 × 10^−8^ in the GWAS summary data. Second, the robustness of the IVs was evaluated using the *F* statistic; IVs with an *F* value below 10 were considered weak and thus removed from the MR study to mitigate bias. The *F* statistic was determined using the formula: *F* = [(N − *K* − 1)/*k*] × [*R*^2^/(1 − *R*^2^)],^[23]^ in which N indicates the GWAS sample size, and *K* refers to the number of IVs included. *R*^2^ was calculated as *R*^2^ = [beta^2^]/[se^2^ × *N* + beta^2^],^[24]^ where beta represents the SNP exposure effect, and se is the standard error of the SNP exposure effect.^[25]^ Third, the exposure and outcome GWAS datasets were harmonized to ensure that the effect size for the exposure and outcome correspond to the same allele. Palindromic genetic variants with ambiguous allele frequencies or incompatible alleles were eliminated. In the reverse MR analysis, the SNP screening process was consistent with the aforementioned procedure.

### 2.4. Statistical analyses

Our study employed 5 distinct MR analyses: the MR-Egger regression, the weighted median, the inverse-variance weighted (IVW), the simple mode, and the weighted mode methods. Notably, the IVW method is regarded as the most robust for MR analysis.^[26]^ Besides, our team applied a multi-tiered MR-pleiotropy residual sum and outlier (MR-PRESSO) analysis to guarantee the reliability of our results by detecting and correcting any statistical outliers.

In addition, we performed several sensitivity analyses to verify the robustness of the final results.^[27]^ Initially, MR-Egger regression was applied to test directional pleiotropy.^[28,29]^ An intercept with statistical significance (*P* < .05) in the MR-Egger analysis suggests the presence of horizontal pleiotropy. Additionally, Cochran *Q* statistic was utilized to evaluate heterogeneity. A statistically significant Cochran *Q* (*P* < .05) indicates heterogeneity within the analysis. Lastly, leave-one-out sensitivity analyses were performed by removing a single SNP at a time to assess whether the variant would drive the association between the exposure and outcome variables.

The Bonferroni correction method was applied to adjust for multiple comparisons, setting the threshold for statistical significance at *P* < .005 (0.05/10), in line with the number of BMDs assessed. *P*-values ranging from .005 to .05 were interpreted as providing suggestive evidence of a potential causal relationship.^[30]^ The results of the causal associations were quantified as odds ratios (ORs) with corresponding 95% confidence intervals (CI). These analyses were conducted using the TwoSampleMR (version 0.5.7) and MR-PRESSO (version 1.0) packages on the R software platform (version 4.3.2).

## 3. Results

### 3.1. Stage 1: forward MR analysis of the effects of GERD on BMD

After removing SNPs that may be associated with confounding factors and those that are palindromic, we pinpointed the relevant SNPs for TB-BMD along with its 5 subcategories (TB-BMD-1 to TB-BMD-5), as well as for FN-BMD, LS-BMD, H-BMD, and UF-BMD, to be used in the MR analyses of this research. We selected 77 SNPs for each of the first 9 indicators and 76 SNPs for UF-BMD. The *F* statistics for all selected SNPs were above 10, suggesting robust instruments were free of weak instrument bias (Table S2, Supplemental Digital Content, https://links.lww.com/MD/O637).

**Figure 2.**
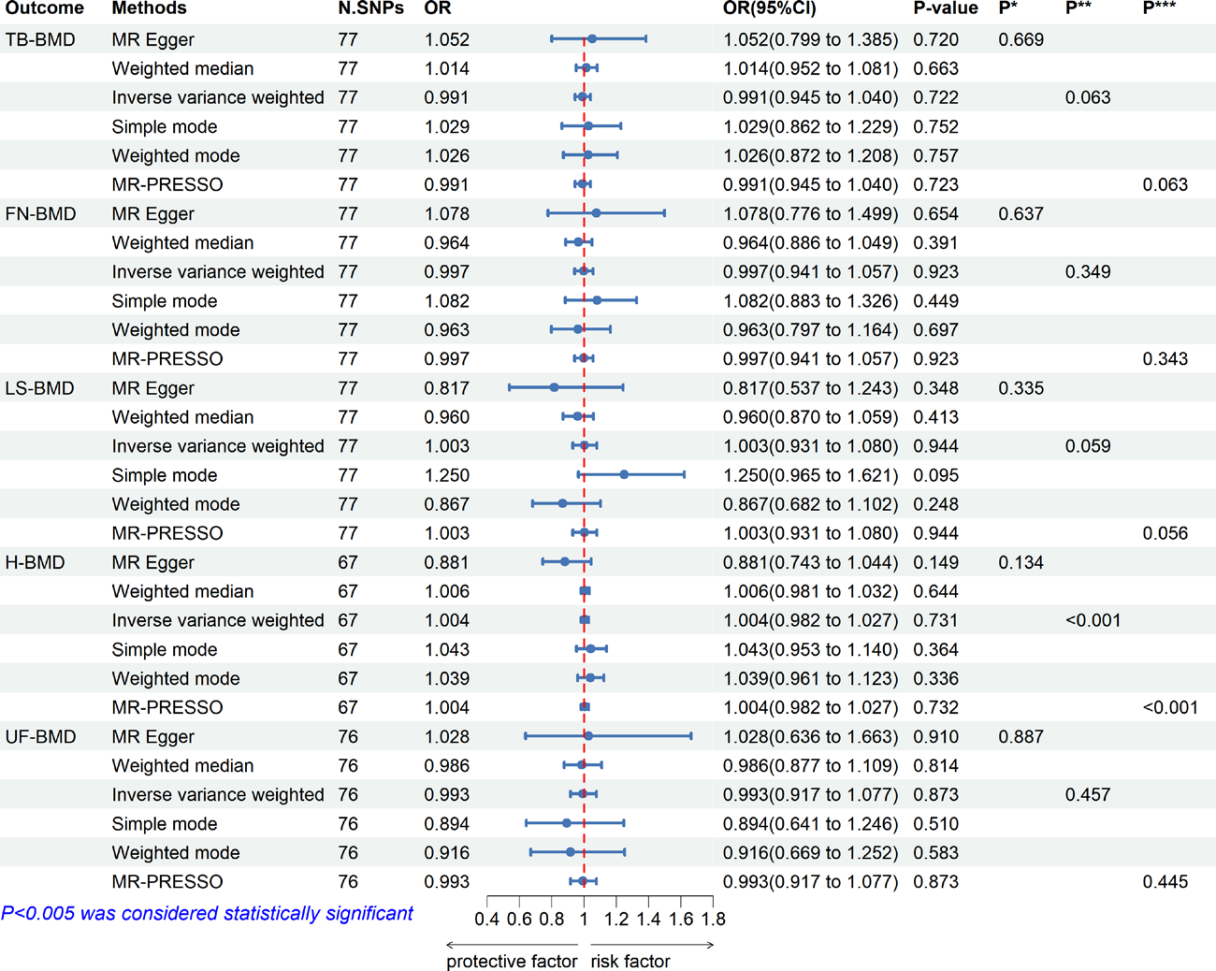
Causal effects of GERD on BMD at different sites. CI = confidence interval, FN-BMD = femoral neck bone mineral density, GERD = gastroesophageal reflux disease, H-BMD = heel bone mineral density, LS-BMD = lumbar spine bone mineral density, N SNPs = number of single nucleotide polymorphism, OR = odds ratio, P^*^ = *P*-value for MR-Egger intercept test, P^**^ = *P*-value for Cochran *Q* test, P^***^ = *P*-value for MR-PRESSO global test, TB-BMD = total body bone mineral density, UF-BMD = ultra-distal forearm bone mineral density.

**Figure 3.**
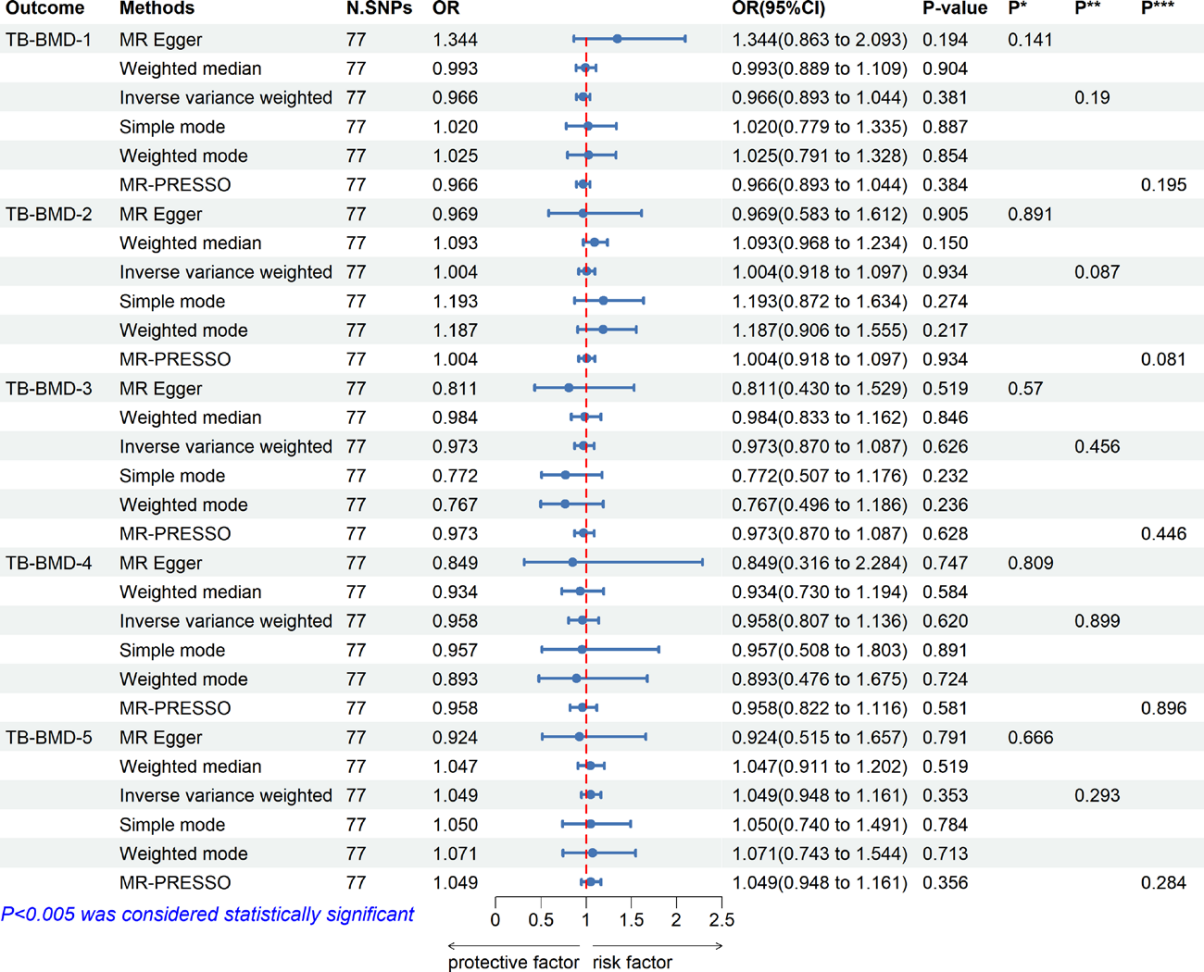
Causal effects of GERD on TB-BMD in different age groups. CI = confidence interval, GERD = gastroesophageal reflux disease, N SNPs = number of single nucleotide polymorphism, OR = odds ratio, P^*^ = *P*-value for MR-Egger intercept test, P^**^ = *P*-value for Cochran *Q* test, P^***^ = *P*-value for MR-PRESSO global test, TB-BMD-1 = total body bone mineral density (age over 60), TB-BMD-2 = total body bone mineral density (age 45–60), TB-BMD-3 = total body bone mineral density (age 30–45), TB-BMD-4 = total body bone mineral density (age 15–30), TB-BMD-5 = total body bone mineral density (age 0–15).

The results of the MR analyses are shown in Figures 2 and 3. No significant causal relationship between GERD and any of the BMD susceptibilities was observed in this study. Results from the MR-Egger and weighted median methods were in significant agreement with the IVW method regarding the direction of effect, affirming the reliability of our findings (Figs 2 and 3). Utilizing the remaining IVs, we performed the first MR-PRESSO test; the distortion test results identified ten significant outliers in H-BMD, and the MR-PRESSO global test indicated heterogeneity in H-BMD (*P* < .001). In other subgroups, no outliers were identified. After removing the outliers, we conducted a second MR-PRESSO test; the MR-PRESSO distortion test did not reveal any significant outlier in H-BMD, while the MR-PRESSO global test indicated the presence of significant heterogeneity in H-BMD (*P* < .001). Even when outliers were removed, genetically predicted GERD remained unrelated to the risk of H-BMD. Furthermore, the MR-Egger intercept test results yielded no evidence of pleiotropy at any level (all *P* > .05) (Figs. 2 and 3).

The heterogeneity between individual SNP estimates in all groups except for H-BMD was not significant (Figs. 2 and 3). Given that the *P*-value from Cochran *Q* test and the MR-PRESSO global test for H-BMD yielded *P*-values below .05, the MR-PRESSO method was selected as the primary analytical approach for the H-BMD groups.^[31]^ While IVW was used as the primary analytical method for the other groups, no significant heterogeneity was found.

### 3.2. Stage 2: reverse MR analysis of the effects of BMD on GERD

Upon rigorous quality control filtering (*P* < 5 × 10^−8^, *r*^2^<.001, *F* > 10), a selection of 57 SNPs for TB-BMD, 13 for TB-BMD-1, 15 for TB-BMD-2, 8 for TB-BMD-3, 8 for TB-BMD-4, 6 for TB-BMD-5, 363 for H-BMD, 5 for UF-BMD, 13 for FN-BMD, and 13 for LS-BMD were identified to serve as IVs. In selecting these SNPs, we eliminated palindromes and invalid SNPs. The calculated *F* statistics showed a strong correlation between IV and exposure, with all F-values exceeding the threshold of 10 (Table S2, Supplemental Digital Content, https://links.lww.com/MD/O637).

Strikingly, in the IVW analysis, a higher genetic predisposition towards TB-BMD, TB-BMD-1, and TB-BMD-3 was linked to a reduced risk of GERD. Specifically, the ORs for TB-BMD, TB-BMD-1, and TB-BMD-3 were 0.946 (95% CI, 0.913–0.981; *P* = .003), 0.919 (95% CI, 0.885–0.954; *P* < .001), and 0.945 (95% CI, 0.915–0.977; *P* = .001), respectively (Table 2). The MR-PRESSO distortion test identified 1 outlier in TB-BMD, and the MR-PRESSO global test showed heterogeneity in TB-BMD (*P* < .001). Upon the exclusion of this outlier, subsequent MR-PRESSO test revealed no significant outliers; however, the global test continued to demonstrate heterogeneity (*P* = .017). After removing this outlier, the IVW analysis further confirmed that a higher genetic predisposition towards TB-BMD is associated with a reduced risk of GERD (95% CI, 0.910–0.972; *P* < .001).

**Table 2:** Associations between genetically predicted BMD at different sites and in different age groups and GERD.

Exposure	Outcome	IVW					PLEIO test		Cochran *Q* test (IVW)		MR-PRESSO global test	
		nSNPs	Beta	SE	OR (95% CI)	*P*	Intercept	*P*	*Q*	*P*	No. of Outliers	*P*
TB-BMD	GERD*	57	−0.055	0.018	0.946 (0.913–0.981)	.003	0.002	.379	98.240	<0.001	1	<.001
TB-BMD	GERD†	56	−0.061	0.017	0.941 (0.910–0.972)	<.001	0.001	.554	80.768	.013	NA	0.017
TB-BMD-1	GERD	13	−0.085	0.019	0.919 (0.885–0.954)	<.001	−0.003	.619	12.498	.407	NA	.458
TB-BMD-2	GERD	15	−0.048	0.020	0.953 (0.916–0.991)	.015	0.015	.029	21.644	.086	NA	.093
TB-BMD-3	GERD	8	−0.056	0.017	0.945 (0.915–0.977)	.001	0.001	.896	6.240	.512	NA	.508
TB-BMD-4	GERD*	8	−0.054	0.024	0.947 (0.903–0.993)	.024	−0.002	.912	17.187	.016	1	.035
TB-BMD-4	GERD†	7	−0.077	0.017	0.926 (0.896–0.957)	<.001	0.009	.468	3.648	.724	NA	.797
TB-BMD-5	GERD	6	−0.041	0.029	0.960 (0.907–1.016)	.159	0.021	.099	8.028	.155	NA	.185
H-BMD	GERD*	363	−0.014	0.015	0.986 (0.958–1.015)	.334	0.001	.485	792.343	<.001	9	<0.001
H-BMD	GERD†	354	−0.024	0.013	0.976 (0.952–1.001)	.063	<0.001	.821	578.829	<.001	NA	<.001
UF-BMD	GERD	5	−0.019	0.021	0.981 (0.942–1.022)	.365	0.010	.206	3.310	.507	NA	.491
FN-BMD	GERD*	13	−0.059	0.042	0.943 (0.868–1.024)	.162	0.002	.917	39.412	<.001	2	<.001
FN-BMD	GERD†	11	−0.066	0.028	0.936 (0.887–0.988)	.016	−0.003	.748	12.737	.239	NA	.241
LS-BMD	GERD	13	−0.074	0.029	0.929 (0.877–0.984)	.011	−0.004	.718	21.158	.048	NA	.060

Beta = effect estimate, CI = confidence interval, FN-BMD = Femoral neck bone mineral density, GERD = gastroesophageal reflux disease, H-BMD = heel bone mineral density, IVW = inverse-variance-weighted, LS-BMD = lumbar spine bone mineral density, MR = Mendelian randomization, No. = number, OR = odds ratio, PLEIO = pleiotropic locus exploration and interpretation using optimal test, PRESSO = pleiotropy residual sum and outlier, *Q* = Cochran *Q* statistic, SE = standard error, SNP = single nucleotide polymorphism, TB-BMD = total body bone mineral density, TB-BMD-1 = total body bone mineral density (age over 60), TB-BMD-2 = total body bone mineral density (age 45–60), TB-BMD-3 = total body bone mineral density (age 30–45), TB-BMD-4 = total body bone mineral density (age 15–30), TB-BMD-5 = total body bone mineral density (age 0–15), UF-BMD = ultra-distal forearm bone mineral density.

* Results after the first deletion of outliers displayed by the MR-PRESSO analysis.

† Results after the second deletion of outliers displayed by the MR-PRESSO analysis.

Furthermore, a higher genetic predisposition to TB-BMD-2 (OR = 0.953, 95% CI = 0.916–0.991, *P* = .015), TB-BMD-4 (OR = 0.947, 95% CI = 0.903–0.993, *P* = .024), and LS-BMD (OR = 0.929, 95% CI = 0.877–0.984, *P* = .011) was suggestively associated with a reduced risk of GERD. The MR-PRESSO distortion test identified 1 outlier in TB-BMD-4, and the MR-PRESSO global test showed heterogeneity in TB-BMD-4 (*P* = .035). Upon the exclusion of this outlier, the MR-PRESSO test revealed no significant outliers, and the MR-PRESSO global test did not show heterogeneity (*P* = .797). After removing the outlier, the IVW analysis indicated that a higher genetic predisposition to TB-BMD-4 remains suggestively associated with a reduced risk of GERD (OR = 0.826, 95% CI = 0.896–0.957, *P* < .001).

For TB-BMD-5 (OR = 0.960, 95% CI = 0.907–1.016, *P* = .159), H-BMD (OR = 0.986, 95% CI = 0.958–1.015, *P* = .334), UF-BMD (OR = 0.981, 95% CI = 0.942–1.022, *P* = .365), and FN-BMD (OR = 0.943, 95% CI = 0.868–1.024, *P* = .162), no significant causal association with GERD was observed. The MR-PRESSO distortion test identified 9 outliers in H-BMD and 2 in FN-BMD. The MR-PRESSO global test also indicated significant heterogeneity in H-BMD and FN-BMD (H-BMD: *P* < .001; FN-BMD: *P* < .001). After adjusting for outliers, the MR-PRESSO test did not identify any significant anomalies for most groups; however, the global test for H-BMD still indicated notable heterogeneity (*P* < .001). Following the exclusion process, the IVW suggested a suggestive causal link between FN-BMD and GERD (OR = 0.936, 95% CI = 0.887–0.988, *P* = .016). Notably, the findings from MR-Egger and weighted median analyses were in substantial agreement with the IVW approach in terms of directional consistency (Table 2). Furthermore, the MR-Egger intercept test results yielded no evidence of pleiotropy in any subgroup except for TB-BMD-2 (*P* = .029).

The heterogeneity between individual SNP estimated in all groups except for TB-BMD, H-BMD, and LS-BMD was nonsignificant. MR-PRESSO was employed as the primary analytical approach for TB-BMD and H-BMD, owing to the *P*-values of the MR-Egger *Q* test and the MR-PRESSO global test being below the threshold of .05. Other BMD subgroups were analyzed using IVW as the primary method.

**Figure 4.**
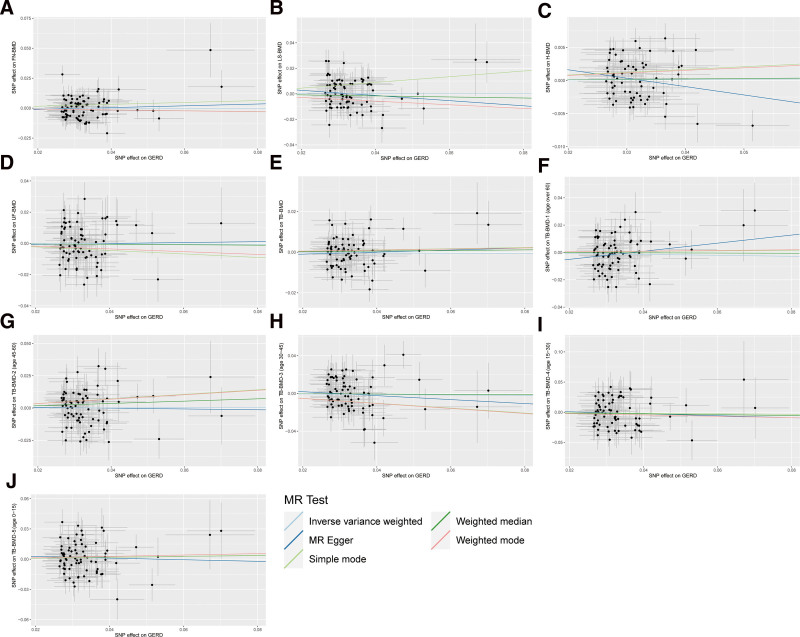
MR scatter plots for the relationship of GERD with BMDs. (A) Causal estimates for GERD on FN-BMD; (B) causal estimates for GERD on LS-BMD; (C) causal estimates for GERD on H-BMD; (D) causal estimates for GERD on UF-BMD; (E) causal estimates for GERD on TB-BMD; (F) causal estimates for GERD on TB-BMD-1 (age over 60); (G) causal estimates for GERD on TB-BMD-2 (age 45–60); (H) causal estimates for GERD on TB-BMD-3 (age 30–45); (I) causal estimates for GERD on TB-BMD-4 (age 15–30); (J) causal estimates for GERD on TB-BMD-5 (age 0–15). The slope of each line corresponds to the causal estimates for each method. Individual SNP effect on the outcome (point and vertical line) against its effect on the exposure (point and horizontal line) is delineated in the background.

Funnel plots demonstrated that the distribution of causal effect estimates was symmetrical, suggesting an absence of bias in the results. Furthermore, leave-one-out sensitivity analyses indicated that, after sequentially excluding each SNP, the results of the IVW analyses for the remaining SNPs were consistent with those that incorporated all SNPs. None of the SNPs exhibited a significant influence on the causal association estimates. The results are visualized in Figure 4 and Figures S1–S5, Supplemental Digital Content, https://links.lww.com/MD/O638.

## 4. Discussion

Our team utilized a bidirectional MR approach to assess the potential causal relationships between GERD and OP. The MR analysis did not reveal a causal link between an increased genetic predisposition to GERD and reduced BMD/OP. Remarkably, our findings indicated an association that a genetic increase in BMD correlates with a diminished risk of developing GERD. Our sensitivity analyses further confirmed the robustness of the association.

Some existing observational studies have suggested that long-term use of PPIs is associated with an increased incidence of fractures in both the hip and vertebrae.^[11,32]^ The heightened risk is believed to be associated with achlorhydria resulting from prolonged PPIs usage, which could disrupt the body’s calcium and vitamin B_12_ absorption, potentially leading to bone density reduction.^[33]^ However, these studies were observational, so the risk remains controversial and uncertain. Other studies have found no association between PPIs and OP or bone loss and no association between PPIs and B_12_ deficiency in elderly patients.^[15,34]^ Therefore, a definitive causal link between GERD and OP has yet to be established. Although a MR approach had been employed to assess causality, it revealed no causal link between a genetically elevated risk of GERD and lower BMD or OP.

Previous literature has reported that OP frequently accompanies GERD, yet their causal relationship remains unclear. Chen et al^[13]^ found that the cumulative incidence of OP was significantly higher in the GERD cohort than in the control cohort. Some researchers have speculated that OP and OP-related kyphosis are the main risk factors for the increased incidence of GERD and/or esophageal hiatal hernia in elderly women.^[2,25]^ Yamaguchi et al^[2]^ conducted a study on 18 Japanese postmenopausal women with refractory reflux esophagitis and 57 control subjects without the condition to observe the incidence of multiple osteoporotic vertebral fractures and esophageal hiatal hernias. They found a notable link between the occurrence of multiple vertebral fractures and persistent cases of reflux esophagitis. This link is even more evident in cases where a hiatal hernia is concurrently present. Kusano et al^[35]^ reported a positive correlation between the size of hiatal hernias assessed by endoscopy and the severity of kyphosis evaluated using postural photographs of 100 elderly Japanese women. However, these observational studies did not assess the degree of vertebral fracture (thoracic or lumbar) or detail spinal alignment (e.g., angle of thoracic and lumbar kyphosis). Thus, we know little about the effects of thoracic and lumbar kyphosis and vertebral fracture on GERD symptoms. Miyakoshi research revealed a positive relationship between the total frequency scale for GERD score symptoms and lumbar kyphosis and lumbar vertebral fractures.^[36]^ Multivariate logistic regression analysis has identified the degree of lumbar kyphosis and the count of lumbar vertebral fractures as significant risk factors for the prevalence of GERD. Of these, every 1-degree increase in lumbar kyphosis and every additional lumbar vertebral fracture were associated with approximately 1.1 and 1.9 times higher odds of developing GERD, respectively. Our MR results are consistent with these observational analyses. However, these previous studies were predominantly case-control or retrospective, with potential confounding risk factors. By contrast, our MR analysis avoids the issues of reverse causation and confounding factors, which can provide evidence to support the potential causal effect of OP on the risk of GERD.

Several mechanisms can explain the causal relationship between OP and GERD. Gastroesophageal reflux is generally considered a manifestation of decreased function at the gastroesophageal junction,^[37]^ with muscle abnormalities and elevated intra-abdominal pressure also contributing. A strong positive correlation exists between intra-abdominal pressure and lumbar compression force,^[38]^ and an increase in intra-abdominal pressure can alleviate the pressure on the lumbar spine.^[39]^ A kyphotic lumbar spine with multiple vertebral fractures might thus raise the intra-abdominal pressure to alleviate compression, resulting in pressure on the esophagus, thereby predisposing the individual to GERD.^[36]^ Bisphosphonates are selective inhibitors of osteoclast-mediated bone resorption used in the treatment and prevention of OP.^[40]^ They are known to effectively prevent the occurrence of new vertebral fractures.^[41,42]^ However, they could cause gastrointestinal symptoms such as heartburn more than placebos do^[41–44]^ and thus may aggravate GERD symptoms in elderly patients with kyphosis or multiple vertebral fractures. GERD is believed to develop from an inflammatory process caused by chronic gastroesophageal reflux, which leads to mucosal injury and DNA damage.^[45]^ Inflammatory mediators such as NF-κB are associated with the induction of CDX genes, which play a crucial role in the initiation of Barrett esophagus.^[46,47]^ Bisphosphonate users may develop esophagitis, esophageal erosions, and esophageal ulcers due to direct topical injury.^[48]^ Oral bisphosphonates may be associated with GERD through a common inflammatory process. Consequently, an investigation into the intricate interplay between OP and GERD could lead to the identification of new therapeutic targets for patients suffering from these conditions.

To the authors’ knowledge, this is the first MR study to explore the causal relationship between GERD and lower BMD/OP that no one has so far attempted. Leveraging multiple IVs derived from large GWAS on GERD and BMD, our investigation has enhanced statistical power over causality detection. In addition, our stratified analysis of BMD, which sorted data into different groups according to age stages and skeletal sites, has clarified the causal relationship between BMD and GERD by age and site. However, our study still has some limitations. First, given that the GWAS data primarily consist of individuals of European descent, the results of this study may not generalize to other populations. Second, the use of self-reported diagnoses for certain GERD cases could potentially impact the trustworthiness of the MR findings. Third, there should have been no overlap of participants between the exposure and outcome studies used in a 2-sample MR analysis. Regrettably, we could not quantify the precise degree of participant overlap in this context.

## 5. Conclusion

In summary, our MR analysis reveals no causal link between genetic predisposition to GERD and the risk of OP or lower BMD. In addition, we found that the genetically predicted decreased BMD/OP significantly caused an increase in the incidence of GERD, suggesting that OP is a potential risk factor for GERD. Therefore, patients with OP should be aware of the potential concurrence of developing GERD.

## Acknowledgments

We are grateful for all the reviewers and all datasets of this paper for providing the free data.

## Author contributions

**Conceptualization:** Hua Jiang.

**Formal analysis:** Qinghua Yang, Shengwang Wei.

**Funding acquisition:** Shengwang Wei, Hua Jiang.

**Investigation:** Junfei Feng, Dun Liu.

**Methodology:** Qinghua Yang, Longao Huang, Junfei Feng.

**Project administration:** Qinghua Yang, Longao Huang.

**Resources:** Qinghua Yang, Longao Huang, Junfei Feng, Shengwang Wei.

**Software:** Hongyuan Xu.

**Supervision:** Hua Jiang.

**Validation:** Junfei Feng.

**Visualization:** Longao Huang.

**Writing – original draft:** Qinghua Yang.

**Writing – review & editing:** Hua Jiang.

## Supplementary Material



## References

[1] Golob AL Laya MB. Osteoporosis: screening, prevention, and management. Med Clin North Am. 2015;99:587–606. 25841602 10.1016/j.mcna.2015.01.010

[2] Yamaguchi T Sugimoto T Yamauchi M Matsumori Y Tsutsumi M Chihara K. Multiple vertebral fractures are associated with refractory reflux esophagitis in postmenopausal women. J Bone Miner Metab. 2005;23:36–40. 10.1007/s00774-004-0538-7 15616892

[3] Blechacz B Gajic O. Severe kyphosis. N Engl J Med. 2008;358:e28. 18550872 10.1056/NEJMicm074057

[4] Wang L Ran L Zha X. Adjustment of DXA BMD measurements for anthropometric factors and its impact on the diagnosis of osteoporosis. Arch Osteoporos. 2020;15:155. 33025208 10.1007/s11657-020-00833-1

[5] Vakil N van Zanten SV Kahrilas P Dent J Jones R; Global Consensus Group. The Montreal definition and classification of gastroesophageal reflux disease: a global evidence-based consensus. Am J Gastroenterol. 2006;101:1900–20; quiz 1943. 16928254 10.1111/j.1572-0241.2006.00630.x

[6] Richter JE Rubenstein JH. Presentation and epidemiology of gastroesophageal reflux disease. Gastroenterology. 2018;154:267–76. 28780072 10.1053/j.gastro.2017.07.045 PMC5797499

[7] Maret-Ouda J Markar SR Lagergren J. Gastroesophageal reflux disease: a review. JAMA. 2020;324:2536–47. 33351048 10.1001/jama.2020.21360

[8] Furukawa N Iwakiri R Koyama T. Proportion of reflux esophagitis in 6010 Japanese adults: prospective evaluation by endoscopy. J Gastroenterol. 1999;34:441–4. 10452674 10.1007/s005350050293

[9] Shiraki MJ. The effect of kyphosis on internal organ function. Osteoporosis Jpn. 2001;9:489–92.

[10] Yepuri G Sukhovershin R Nazari-Shafti TZ Petrascheck M Ghebre YT Cooke JP. Proton pump inhibitors accelerate endothelial senescence. Circ Res. 2016;118:e36–42. 27166251 10.1161/CIRCRESAHA.116.308807 PMC4902745

[11] Targownik LE Lix LM Metge CJ Prior HJ Leung S Leslie WD. Use of proton pump inhibitors and risk of osteoporosis-related fractures. CMAJ. 2008;179:319–26. 18695179 10.1503/cmaj.071330 PMC2492962

[12] Vestergaard P Rejnmark L Mosekilde L. Proton pump inhibitors, histamine H2 receptor antagonists, and other antacid medications and the risk of fracture. Calcif Tissue Int. 2006;79:76–83. 16927047 10.1007/s00223-006-0021-7

[13] Chen CH Lin CL Kao CH. Gastroesophageal reflux disease with proton pump inhibitor use is associated with an increased risk of osteoporosis: a nationwide population-based analysis. Osteoporos Int. 2016;27:2117–26. 26860609 10.1007/s00198-016-3510-1

[14] Fraser LA Leslie WD Targownik LE Papaioannou A Adachi JD; CaMos Research Group. The effect of proton pump inhibitors on fracture risk: report from the Canadian Multicenter Osteoporosis Study. Osteoporos Int. 2013;24:1161–8. 22890365 10.1007/s00198-012-2112-9 PMC5096922

[15] Targownik LE Lix LM Leung S Leslie WD. Proton-pump inhibitor use is not associated with osteoporosis or accelerated bone mineral density loss. Gastroenterology. 2010;138:896–904. 19931262 10.1053/j.gastro.2009.11.014

[16] Burgess S Davey Smith G Davies NM. Guidelines for performing Mendelian randomization investigations: update for summer 2023. Wellcome Open Res. 2019;4:186. 32760811 10.12688/wellcomeopenres.15555.1 PMC7384151

[17] Skrivankova VW Richmond RC Woolf BAR. Strengthening the reporting of observational studies in epidemiology using Mendelian randomization: the STROBE-MR statement. JAMA. 2021;326:1614–21. 34698778 10.1001/jama.2021.18236

[18] Ong JS An J Han X; 23andMe Research team. Multitrait genetic association analysis identifies 50 new risk loci for gastro-oesophageal reflux, seven new loci for Barrett’s oesophagus and provides insights into clinical heterogeneity in reflux diagnosis. Gut. 2022;71:1053–61. 34187846 10.1136/gutjnl-2020-323906 PMC9120377

[19] Medina-Gomez C Kemp JP Trajanoska K. Life-course genome-wide association study meta-analysis of total body BMD and assessment of age-specific effects. Am J Hum Genet. 2018;102:88–102. 29304378 10.1016/j.ajhg.2017.12.005 PMC5777980

[20] Morris JA Kemp JP Youlten SE; 23andMe Research Team. An atlas of genetic influences on osteoporosis in humans and mice. Nat Genet. 2019;51:258–66. 30598549 10.1038/s41588-018-0302-x PMC6358485

[21] Surakka I Fritsche LG Zhou W; Regeneron Genetics Center. MEPE loss-of-function variant associates with decreased bone mineral density and increased fracture risk. Nat Commun. 2020;11:4093. 33097703 10.1038/s41467-020-17315-0 PMC7585430

[22] Zheng HF Forgetta V Hsu YH; AOGC Consortium. Whole-genome sequencing identifies EN1 as a determinant of bone density and fracture. Nature. 2015;526:112–7. 26367794 10.1038/nature14878 PMC4755714

[23] Palmer TM Lawlor DA Harbord RM. Using multiple genetic variants as instrumental variables for modifiable risk factors. Stat Methods Med Res. 2012;21:223–42. 21216802 10.1177/0962280210394459 PMC3917707

[24] Chen B Yan Y Wang H Xu J. Association between genetically determined telomere length and health-related outcomes: a systematic review and meta-analysis of Mendelian randomization studies. Aging Cell. 2023;22:e13874. 37232505 10.1111/acel.13874 PMC10352568

[25] Skrivankova VW Richmond RC Woolf BAR. Strengthening the reporting of observational studies in epidemiology using mendelian randomisation (STROBE-MR): explanation and elaboration. BMJ. 2021;375:n2233. 34702754 10.1136/bmj.n2233 PMC8546498

[26] Burgess S Thompson SG; CRP CHD Genetics Collaboration. Avoiding bias from weak instruments in Mendelian randomization studies. Int J Epidemiol. 2011;40:755–64. 21414999 10.1093/ije/dyr036

[27] Verbanck M Chen CY Neale B Do R. Detection of widespread horizontal pleiotropy in causal relationships inferred from Mendelian randomization between complex traits and diseases. Nat Genet. 2018;50:693–8. 29686387 10.1038/s41588-018-0099-7 PMC6083837

[28] Bowden J Davey Smith G Burgess S. Mendelian randomization with invalid instruments: effect estimation and bias detection through Egger regression. Int J Epidemiol. 2015;44:512–25. 26050253 10.1093/ije/dyv080 PMC4469799

[29] Lee CH Shi H Pasaniuc B Eskin E Han B. PLEIO: a method to map and interpret pleiotropic loci with GWAS summary statistics. Am J Hum Genet. 2021;108:36–48. 33352115 10.1016/j.ajhg.2020.11.017 PMC7820744

[30] Sedgwick P. Multiple hypothesis testing and Bonferroni’s correction. BMJ. 2014;349:g6284. 25331533 10.1136/bmj.g6284

[31] Jin H Lee S Won S. Causal evaluation of laboratory markers in type 2 diabetes on cancer and vascular diseases using various Mendelian randomization tools. Front Genet. 2020;11:597420. 33408737 10.3389/fgene.2020.597420 PMC7780896

[32] Yang YX Lewis JD Epstein S Metz DC. Long-term proton pump inhibitor therapy and risk of hip fracture. JAMA. 2006;296:2947–53. 17190895 10.1001/jama.296.24.2947

[33] Yang YX Metz DC. Safety of proton pump inhibitor exposure. Gastroenterology. 2010;139:1115–27. 20727892 10.1053/j.gastro.2010.08.023

[34] den Elzen WP Groeneveld Y de Ruijter W. Long-term use of proton pump inhibitors and vitamin B12 status in elderly individuals. Aliment Pharmacol Ther. 2008;27:491–7. 18194503 10.1111/j.1365-2036.2008.03601.x

[35] Kusano M Hashizume K Ehara Y Shimoyama Y Kawamura O Mori M. Size of hiatus hernia correlates with severity of kyphosis, not with obesity, in elderly Japanese women. J Clin Gastroenterol. 2008;42:345–50. 18277907 10.1097/MCG.0b013e318037556c

[36] Miyakoshi N Kasukawa Y Sasaki H Kamo K Shimada Y. Impact of spinal kyphosis on gastroesophageal reflux disease symptoms in patients with osteoporosis. Osteoporos Int. 2009;20:1193–8. 18949531 10.1007/s00198-008-0777-x

[37] Fujimoto K. Review article: prevalence and epidemiology of gastro-oesophageal reflux disease in Japan. Aliment Pharmacol Ther. 2004;20:5–8. 15575864 10.1111/j.1365-2036.2004.02220.x

[38] Morris JM Lucas DB Bresler B. Role of the trunk in stability of the spine. J Bone Joint Surg Am. 1961;43:327–51

[39] Lander JE Hundley JR Simonton RL. The effectiveness of weight-belts during multiple repetitions of the squat exercise. Med Sci Sports Exerc. 1992;24:603–9. 1533266

[40] Lin D Kramer JR Ramsey D. Oral bisphosphonates and the risk of Barrett’s esophagus: case-control analysis of US veterans. Am J Gastroenterol. 2013;108:1576–83. 23857477 10.1038/ajg.2013.222 PMC4046950

[41] Harris ST Watts NB Genant HK. Effects of risedronate treatment on vertebral and nonvertebral fractures in women with postmenopausal osteoporosis: a randomized controlled trial. JAMA. 1999;282:1344–52. 10527181 10.1001/jama.282.14.1344

[42] Black DM Cummings SR Karpf DB. Randomised trial of effect of alendronate on risk of fracture in women with existing vertebral fractures. Lancet. 1996;348:1535–41. 8950879 10.1016/s0140-6736(96)07088-2

[43] Cohen S Levy RM Keller M. Risedronate therapy prevents corticosteroid-induced bone loss: a twelve-month, multicenter, randomized, double-blind, placebo-controlled, parallel-group study. Arthritis Rheum. 1999;42:2309–18. 10555025 10.1002/1529-0131(199911)42:11<2309::AID-ANR8>3.0.CO;2-K

[44] Saag KG Emkey R Schnitzer TJ. Alendronate for the prevention and treatment of glucocorticoid-induced osteoporosis. N Engl J Med. 1998;339:292–9. 9682041 10.1056/NEJM199807303390502

[45] Olliver JR Hardie LJ Gong Y. Risk factors, DNA damage, and disease progression in Barrett’s esophagus. Cancer Epidemiol Biomarkers Prev. 2005;14:620–5. 15767340 10.1158/1055-9965.EPI-04-0509

[46] Colleypriest BJ Ward SG Tosh D. How does inflammation cause Barrett’s metaplasia? Curr Opin Pharmacol. 2009;9:721–6. 19828375 10.1016/j.coph.2009.09.005

[47] Chen H Fang Y Tevebaugh W Orlando RC Shaheen NJ Chen X. Molecular mechanisms of Barrett’s esophagus. Dig Dis Sci. 2011;56:3405–20. 21984436 10.1007/s10620-011-1885-6 PMC3750118

[48] Ribeiro A DeVault KR Wolfe JT 3rd Stark ME. Alendronate-associated esophagitis: endoscopic and pathologic features. Gastrointest Endosc. 1998;47:525–8. 9647380 10.1016/s0016-5107(98)70256-1

