# Supplementary material for: Gastroesophageal reflux disease and osteoporosis: A bidirectional Mendelian randomization study
Source: Medicine (Baltimore). 2025 Apr 4;104(14):e42083. doi: 10.1097/MD.0000000000042083 (PMC11977714; doi:10.1097/MD.0000000000042083)
Supplement: Supplementary file 1 [file medi-104-e42083-s001.docx]

**Supplementary Table S1 STROBE-MR checklist of recommended items to address in reports of Mendelian randomization studies**^1^ ^2^

| **Item No.** | **Section** | **Checklist item** | **Page No.** | **Relevant text from manuscript** |
| --- | --- | --- | --- | --- |
| 1 | **TITLE and ABSTRACT** | Indicate Mendelian randomization (MR) as the study’s design in the title and/or the abstract if that is a main purpose of the study | Pages 1-2 | Gastroesophageal reflux disease and osteoporosis: a bidirectional Mendelian randomization study |
|  | **INTRODUCTION** |  |  |  |
| 2 | **Background** | Explain the scientific background and rationale for the reported study. What is the exposure? Is a potential causal relationship between exposure and outcome plausible? Justify why MR is a helpful method to address the study question | Pages 4-5 | Observational studies have shown a close relationship with strong evidence between osteoporosis (OP) and gastroesophageal reflux disease (GERD). While the co-occurrence of GERD and OP is recognized, the exact mechanism by which GERD is complicated by OP is unclear. Elucidating the specific mechanism of OP in individuals suffering from GERD is essential for developing targeted prevention and therapeutic approaches within this patient group. Moreover, while OP has been identified as a risk factor, the exact nature of the relationship between it and GERD remains to be established. |
| 3 | **Objectives** | State specific objectives clearly, including pre-specified causal hypotheses (if any). State that MR is a method that, under specific assumptions, intends to estimate causal effects | Page 5 | We employed a bidirectional Mendelian randomization (MR) analysis to explore the causal relationship between GERD and OP. |
|  | **METHODS** |  |  |  |
| 4 | **Study design and data sources** | Present key elements of the study design early in the article. Consider including a table listing sources of data for all phases of the study. For each data source contributing to the analysis, describe the following: |  |  |
|  | a) | Setting: Describe the study design and the underlying population, if possible. Describe the setting, locations, and relevant dates, including periods of recruitment, exposure, follow-up, and data collection, when available. | Page 6 | The GERD genome-wide association study (GWAS) dataset was sourced from Ong *et al.*, encompassing 129,080 cases and 473,524 controls. The GWAS summary-level data on GERD is retrieved from the GWAS Catalog. In the study by Ong *et al.*, controls were defined as individuals without any history or current occurrence of upper digestive system disorders. GERD cases were defined based on a combination of self-reported GERD symptoms such as heartburn, the use of GERD medication, and hospital records based on ICD-10 codes. Every participant was obligated to fill out a consent form, and the study by Ong *et al.* was approved by the QIMR Berghofer’s Human Research Ethics Committee under project ID 3501.  To examine age-specific and site-specific BMD, we used summary-level data of BMD from different age stages and skeletal sites. For the overall BMD, the total body BMD (TB-BMD) was assessed by DXA. The TB-BMD dataset was obtained from the GEnetic Factors for Osteoporosis Consortium (GEFOS) meta-analysis, encompassing 66,628 participants, predominantly of European ancestry (86%), followed by mixed Oceanian (14%) and African American ancestry (2%). This dataset covered five different age stages: ≤15 years (N=11,807), 15-30 years (N=4180), 30-45 years (N=10,062), 45-60 years (N=18,805), ≥60 years (N=22,504). The GWAS datasets for heel BMD (H-BMD) and ultra-distal forearm BMD (UF-BMD) included 426,824 and 21,907 individuals, respectively, all of European descent. H-BMD was estimated by quantitative ultrasound, and UF-BMD was measured by single-energy X-ray absorptiometry. The data for femoral neck BMD (FN-BMD) and lumbar spine BMD (LS-BMD) originated from another GEFOS study, comprising 32,735 and 28,498 participants, mostly of white British origin. FN-BMD and LS-BMD were assessed by DXA. Relevant ethics committees approved all studies contributing data to these analyses. |
|  | b) | Participants: Give the eligibility criteria, and the sources and methods of selection of participants. Report the sample size, and whether any power or sample size calculations were carried out prior to the main analysis | Not  mentioned |  |
|  | c) | Describe measurement, quality control and selection of genetic variants | Page 7 | First, genome-wide divergent SNPs with *P*<5×10^–8^ and linkage disequilibrium of r^2^<0.001 and a genetic distance of 10,000 kb were selected as IVs. For TB-BMD-4, a more lenient significance threshold of *P*<5×10^−6^ was adopted because only a single SNP was identified at the more stringent level of *P*<5×10^−8^ in the GWAS summary data. Second, the robustness of the IVs was evaluated using the F statistic; IVs with an F value below 10 were considered weak and thus removed from the MR study to mitigate bias. The F statistic was determined using the formula: F = [(N-K-1)/k]×[R^2^/(1-R^2^)], where N is the GWAS sample size, and K is the number of variants comprising the instrument. R^2^ was calculated as R^2^ = [beta^2^]/[se^2^×N+beta^2^], where beta represents the SNP exposure effect, and se is the standard error of the SNP exposure effect. Third, the exposure and outcome GWAS datasets were harmonized to ensure that the effect size for the exposure and outcome corresponded to the identical effect alleles. Palindromic genetic variants with ambiguous allele frequencies or incompatible alleles were excluded. In the reverse MR analysis, the SNP screening process was consistent with the aforementioned procedure. |
|  | d) | For each exposure, outcome, and other relevant variables, describe methods of assessment and diagnostic criteria for diseases | Table 1 | Details of the genome-wide association studies and datasets used in this study |
|  | e) | Provide details of ethics committee approval and participant informed consent, if relevant | Page 6 | All data analyzed in this study were obtained from publicly available databases in which ethical approval was obtained for each cohort, and informed consent was obtained from all participants prior to participation. |
| 5 | **Assumptions** | Explicitly state the three core IV assumptions for the main analysis (relevance, independence and exclusion restriction) as well assumptions for any additional or sensitivity analysis | Pages 5, 7, and 8 | We selected IV based on three generally recognized assumptions: (1) the IV needs to be strongly associated with exposure; (2) the IV is independent of confounders; and (3) the IV is linked to the outcome solely through the exposure, without a direct association with the outcome. Three methods of sensitivity analysis and horizontal pleiotropy testing are also described: weighted median (WM), MR-Egger and Mendelian Randomization pleiotropy residual sum and outlier (MR-PRESSO), and the use of F statistics to assess statistical power and Cochran’s Q test to assess heterogeneity are reported. |
| 6 | **Statistical methods: main analysis** | Describe statistical methods and statistics used |  |  |
|  | a) | Describe how quantitative variables were handled in the analyses (i.e., scale, units, model) | Not mentioned | This research does not involve any transformations of quantitative variables. |
|  | b) | Describe how genetic variants were handled in the analyses and, if applicable, how their weights were selected | Pages 7-8 | Three analysis methods were employed in this study: Inverse variance weighting (IVW), MR-Egger, and WM. The IVW method, considered the primary method for assessing causality, yielded a nominally significantly correlated result when the *P* value was less than 0.05. To ensure the robustness of the MR results, both MR-Egger and WM methods were employed as complementary approaches. The Cochran’s Q test was used to estimate the heterogeneity of SNPs. Additionally, to ensure the reliability of the results, a leave-one-out analysis was carried out. To identify horizontal pleiotropy, the MR-egger intercept was utilized. Causality was evaluated using the odds ratio (OR) and 95% confidence interval (CI). |
|  | c) | Describe the MR estimator (e.g. two-stage least squares, Wald ratio) and related statistics. Detail the included covariates and, in case of two-sample MR, whether the same covariate set was used for adjustment in the two samples | Pages 7-8 | The IVW method was used as the primary analytical method for estimating causal effects, which is an extension of the Wald ratio estimator based on the principles of meta-analysis. To further demonstrate the stability and directionality of the results, in addition to the IVW method, we used MR-Egger, WM, Simple mode, and Weighted mode for the auxiliary assessment of causality. In addition, we applied Bayesian model averaging (BMA) to further validate the results of IVW *P*-value. |
|  | d) | Explain how missing data were addressed | Not mentioned |  |
|  | e) | If applicable, indicate how multiple testing was addressed | Page 8 | The Bonferroni correction method was applied to adjust for multiple comparisons, setting the threshold for statistical significance at *P*<0.005 (0.05/10), in line with the number of BMDs assessed. |
| 7 | **Assessment of assumptions** | Describe any methods or prior knowledge used to assess the assumptions or justify their validity | Page 8 | We computed the F statistic for IVs to assess the extent of weak instrumental bias. Only IVs with F > 10 were retained to avoid bias caused by weak IVs. |
| 8 | **Sensitivity analyses and additional analyses** | Describe any sensitivity analyses or additional analyses performed (e.g. comparison of effect estimates from different approaches, independent replication, bias analytic techniques, validation of instruments, simulations) | Pages 7-8 | Firstly, to mitigate the impact of heterogeneity on the causal effect, Cochran’s Q test was employed to evaluate heterogeneity. If the *P*-value exceeded 0.05, the influence of heterogeneity on the causal effect was deemed negligible. Conversely, in the presence of significant heterogeneity (*P*-value < 0.05), the IVW random effects model was applied to mitigate the impact of heterogeneity on the causal effect. Then, considering the influence of unknown confounders on genetic diversity and causal effects, we used MR Egger regression to assess whether the included SNPs were potentially horizontally pleiotropic and the results of horizontally pleiotropic (*P*<0.05) were excluded. In addition, we used MR-PRESSO to identify and remove any outliers with significant differences. After excluding outliers, the MR analysis was repeated, and the causal effect was reassessed. Finally, a leave-one-out sensitivity analysis is performed to assess the robustness of the results. |
| 9 | **Software and pre-registration** |  |  |  |
|  | a) | Name statistical software and package(s), including version and settings used | Page 8 | All analyses were conducted using R version 4.3.2, with the software packages “Two-SampleMR” and “MR-PRESSO”. To visualize the MR analysis, forest plots, scatter plots, and leave-one-out plots were generated using the data analysis function of the Rstudio platform. |
|  | b) | State whether the study protocol and details were pre-registered (as well as when and where) | Not mentioned |  |
|  | **RESULTS** |  |  |  |
| 10 | **Descriptive data** |  |  |  |
|  | a) | Report the numbers of individuals at each stage of included studies and reasons for exclusion. Consider use of a flow diagram | Table 1 | The numbers of individuals included study is already provided in the Table 1. |
|  | b) | Report summary statistics for phenotypic exposure(s), outcome(s), and other relevant variables (e.g. means, SDs, proportions) | Table 2  Figures 2-3 | Summary data on exposure and outcomes are shown in Table 2 and Figures 2 and 3. |
|  | c) | If the data sources include meta-analyses of previous studies, provide the assessments of heterogeneity across these studies | Table 2  Figures 2-3 | The Cochran’s Q test was used to estimate the heterogeneity of SNPs, detailed data are provided in Table 2 and Figures 2 and 3. |
|  | d) | For two-sample MR:  i.  Provide justification of the similarity of the genetic variant-exposure associations between the exposure and outcome samples  ii.  Provide information on the number of individuals who overlap between the exposure and outcome studies | Table 2  Figures 2-3 | The data presented in this study were derived exclusively from European population samples. These samples were obtained from independent GWAS databases, ensuring minimal overlap and bias, detailed data on the number of individuals in the exposure and outcome samples are provided in Table 2 and Figures 2 and 3. |
| 11 | **Main results** |  |  |  |
|  | a) | Report the associations between genetic variant and exposure, and between genetic variant and outcome, preferably on an interpretable scale | Pages 8-10 | There were no significant causal links between genetic inclination towards GERD and reduced BMD levels. Nonetheless, the genetic evidence suggests a causal link between higher BMD levels and lower incidence of GERD (TB-BMD: OR=0.941, 95% CI: 0.910-0.972, *P*<0.001; TB-BMD-1: OR=0.919, 95% CI: 0.885-0.954, *P*<0.001; TB-BMD-3: OR=0.945, 95% CI: 0.915-0.977, *P*=0.001; TB-BMD-4: OR=0.926, 95% CI: 0.896-0.957, *P*<0.001). Sensitivity analyses corroborated our findings. |
|  | b) | Report MR estimates of the relationship between exposure and outcome, and the measures of uncertainty from the MR analysis, on an interpretable scale, such as odds ratio or relative risk per SD difference | Not mentioned |  |
|  | c) | If relevant, consider translating estimates of relative risk into absolute risk for a meaningful time period | Not mentioned |  |
|  | d) | Consider plots to visualize results (e.g. forest plot, scatterplot of associations between genetic variants and outcome versus between genetic variants and exposure) | Figures 2-4  Supplementary Figures 1-5 | The results are visualized in Figures 2-4 and Supplementary Figures 1-5. |
| 12 | **Assessment of assumptions** |  |  |  |
|  | a) | Report the assessment of the validity of the assumptions |  | Firstly, we selected the SNPs of GERD as instrumental variables, which have a strong association with BMD, allowing us to perform Mendelian randomization inferences, and the large F statistics indicate that these analyzes will not be affected by weak instrument bias. Secondly, the selected SNPs were ensured to have no association with any confounding factors that could influence the relationship between exposure and outcome. Lastly, the SNPs were confirmed to only impact the outcome through exposure factors. |
|  | b) | Report any additional statistics (e.g., assessments of heterogeneity across genetic variants, such as *I^2^*, Q statistic or E-value) | Table 2  Figures 2-3 | Cochran’s Q and I^2^ statistics were calculated to check for the presence of heterogeneity (dispersion of SNP effects) which can indicate pleiotropy. We found little evidence of heterogeneity for the association between BMD and GERD (see Table 2 and Figures 2 and 3 for further information). |
| 13 | **Sensitivity analyses and additional analyses** |  |  |  |
|  | a) | Report any sensitivity analyses to assess the robustness of the main results to violations of the assumptions | Table 2  Figures 2-3 | MR-Egger, WM and IVW-random-effects model methods were provided as sensitivity analyses. When significant pleiotropy was present, we used the MR-PRESSO method to remove outlier SNPs and calculate corrected ORs and CIs. |
|  | b) | Report results from other sensitivity analyses or additional analyses | Table 2  Figures 2-3 | MR-Egger, WM and IVW-random-effects model methods were provided as sensitivity analyses. When significant pleiotropy was present, we used the MR-PRESSO method to remove outlier SNPs and calculate corrected ORs and CIs. |
|  | c) | Report any assessment of direction of causal relationship (e.g., bidirectional MR) | Table 2  Figures 2-3 | We conducted a bidirectional MR analysis. The forward analysis did not find a causal relationship between GERD and OP or reduced BMD, as detailed in Figure 2 and Figure 3. However, the reverse analysis demonstrated a causal relationship between OP or reduced BMD and GERD. See Table 2 for details. |
|  | d) | When relevant, report and compare with estimates from non-MR analyses |  | This study does not involve non-MR studies. |
|  | e) | Consider additional plots to visualize results (e.g., leave-one-out analyses) | Figures 2-4  Supplementary Figures 1-5 | To visualize the MR analysis, forest plots, scatter plots, and leave-one-out plots were generated using the data analysis function of the Rstudio platform, as detailed in Figures 2-4 and Supplementary Figures 1-5. |
|  | **DISCUSSION** |  |  |  |
| 14 | **Key results** | Summarize key results with reference to study objectives | Page 11 | The MR analysis did not reveal a causal link between an increased genetic predisposition for GERD and reduced BMD/OP. Remarkably, our findings indicated an association where a genetic increase in BMD correlates with a diminished risk of developing GERD. |
| 15 | **Limitations** | Discuss limitations of the study, taking into account the validity of the IV assumptions, other sources of potential bias, and imprecision. Discuss both direction and magnitude of any potential bias and any efforts to address them | Page 14 | However, our study has some limitations. First, given that the GWAS data primarily consist of individuals of European descent, the results of this study may not generalize to other populations. Second, the use of self-reported diagnoses for certain GERD cases could potentially impact the trustworthiness of the MR findings. Third, there should be no overlap of participants between the exposure and outcome studies used in a two-sample MR analysis. Regrettably, we could not quantify the precise degree of participant overlap in this context. |
| 16 | **Interpretation** |  |  |  |
|  | a) | Meaning: Give a cautious overall interpretation of results in the context of their limitations and in comparison with other studies | Pages 11-13 | In this manuscript, the content of this item is discussed a lot, and the MR results are reasonably interpreted by comparing them with several published studies. |
|  | b) | Mechanism: Discuss underlying biological mechanisms that could drive a potential causal relationship between the investigated exposure and the outcome, and whether the gene-environment equivalence assumption is reasonable. Use causal language carefully, clarifying that IV estimates may provide causal effects only under certain assumptions | Page 13 | This research discussed the possible mechanisms by which reductions in BMD or OP may lead to GERD. |
|  | c) | Clinical relevance: Discuss whether the results have clinical or public policy relevance, and to what extent they inform effect sizes of possible interventions | Page 14 | We found that the genetically predicted decreased BMD/OP significantly caused an increase in the incidence of GERD, suggesting that OP is a potential risk factor for GERD. Therefore, patients with OP should be aware of the potential of concurrent GERD development. |
| 17 | **Generalizability** | Discuss the generalizability of the study results (a) to other populations, (b) across other exposure periods/timings, and (c) across other levels of exposure | Page 14 | The majority of participants in our study were of European descent; thus, our results may not be generalizable to other ethnic populations. |
|  | **OTHER INFORMATION** |  |  |  |
| 18 | **Funding** | Describe sources of funding and the role of funders in the present study and, if applicable, sources of funding for the databases and original study or studies on which the present study is based | Pages 14-15 | This project was supported by National Natural Science Foundation of China (82360438), Joint Project on Regional High-Incidence Diseases Research of Guangxi Natural Science Foundation (2024GXNSFDA010043), Guangxi Natural Science Foundation (2023GXNSFAA026339), Liuzhou Science and Technology Plan Project (2021CBB0110) and 2023 Guangxi Basic Research Ability Improvement Project for Young and Middle-aged Teachers at University (2023KY0086). |
| 19 | **Data and data sharing** | Provide the data used to perform all analyses or report where and how the data can be accessed, and reference these sources in the article. Provide the statistical code needed to reproduce the results in the article, or report whether the code is publicly accessible and if so, where | Page 15 | The original contributions presented in the study are included in the article/Supplementary Material. Further inquiries can be directed to the corresponding author. |
| 20 | **Conflicts of Interest** | All authors should declare all potential conflicts of interest | Page 15-16 | The authors declare no competing interests. |

This checklist is copyrighted by the Equator Network under the Creative Commons Attribution 3.0 Unported (CC BY 3.0) license.

1. Skrivankova VW, Richmond RC, Woolf BAR, Yarmolinsky J, Davies NM, Swanson SA, et al. Strengthening the Reporting of Observational Studies in Epidemiology using Mendelian Randomization: the STROBE-MR Statement. JAMA. 2021;326:1614-1621.

2. Skrivankova VW, Richmond RC, Woolf BAR, Davies NM, Swanson SA, VanderWeele TJ, et al. Strengthening the Reporting of Observational Studies in Epidemiology using Mendelian Randomisation (STROBE-MR): Explanation and Elaboration. BMJ. 2021;375:n2233.
