# Supplementary material for: Gastroesophageal reflux disease and osteoporosis: A bidirectional Mendelian randomization study
Source: Medicine (Baltimore). 2025 Apr 4;104(14):e42083. doi: 10.1097/MD.0000000000042083 (PMC11977714; doi:10.1097/MD.0000000000042083)
Supplement: Supplementary file 2 [file medi-104-e42083-s002.docx]

| Supplementary Table S2  Characteristics of the IVs used in MR analysis of bidirectional causal relationships between gastroesophageal reflux disease and osteoporosis | | | | | | | | | | | | | | | | | |
| --- | --- | --- | --- | --- | --- | --- | --- | --- | --- | --- | --- | --- | --- | --- | --- | --- | --- |
| **Outcome** | **Exposure** | **SNP** | **Chr** | **Pos** | **Exposure** | | | | |  | **Outcome** | | | | | **R2** | **F** |
|  |  |  |  |  | **EA** | **OA** | **Beta** | **Se** | **Pval** |  | **EA** | **OA** | **Beta** | **Se** | **Pval** |  |  |
| TB-BMD | GERD | rs10010963 | 4 | 159839313 | T | C | -0.027 | 0.005 | 4.91779E-08 |  | T | C | -0.004 | 0.006 | 0.510 | 4.93648E-05 | 29.749 |
| TB-BMD | GERD | rs1011407 | 2 | 60665768 | G | A | -0.042 | 0.007 | 1.09343E-08 |  | G | A | 0.001 | 0.009 | 0.918 | 5.42079E-05 | 32.668 |
| TB-BMD | GERD | rs10133111 | 14 | 103377321 | A | G | 0.042 | 0.007 | 1.35338E-10 |  | A | G | -0.002 | 0.008 | 0.800 | 6.84149E-05 | 41.230 |
| TB-BMD | GERD | rs1021363 | 10 | 106610839 | G | A | -0.031 | 0.005 | 5.09976E-10 |  | G | A | -0.001 | 0.006 | 0.874 | 6.41154E-05 | 38.639 |
| TB-BMD | GERD | rs10837002 | 11 | 38565727 | G | C | 0.028 | 0.005 | 4.02893E-08 |  | G | C | 0.000 | 0.006 | 0.942 | 5.00063E-05 | 30.135 |
| TB-BMD | GERD | rs11762636 | 7 | 2061111 | A | C | -0.051 | 0.006 | 1.88235E-16 |  | A | C | -0.001 | 0.007 | 0.927 | 0.00011237 | 67.722 |
| TB-BMD | GERD | rs11953061 | 5 | 120144025 | T | C | 0.028 | 0.005 | 3.09949E-08 |  | T | C | -0.007 | 0.006 | 0.266 | 5.08505E-05 | 30.644 |
| TB-BMD | GERD | rs12204714 | 6 | 152235339 | T | C | -0.029 | 0.005 | 7.9241E-09 |  | T | C | -0.003 | 0.006 | 0.677 | 5.52467E-05 | 33.294 |
| TB-BMD | GERD | rs12357321 | 10 | 21790476 | A | G | 0.032 | 0.005 | 1.33325E-09 |  | A | G | -0.002 | 0.006 | 0.814 | 6.10048E-05 | 36.764 |
| TB-BMD | GERD | rs12453010 | 17 | 50316131 | T | C | 0.030 | 0.005 | 1.74606E-09 |  | T | C | -0.007 | 0.006 | 0.209 | 6.01325E-05 | 36.238 |
| TB-BMD | GERD | rs12598916 | 16 | 60658751 | G | C | -0.033 | 0.005 | 6.8729E-10 |  | G | C | -0.001 | 0.006 | 0.902 | 6.31493E-05 | 38.056 |
| TB-BMD | GERD | rs12967855 | 18 | 35138245 | G | A | -0.037 | 0.005 | 1.08793E-12 |  | G | A | 0.006 | 0.006 | 0.316 | 8.40922E-05 | 50.678 |
| TB-BMD | GERD | rs12997558 | 2 | 41704580 | A | G | 0.028 | 0.005 | 3.03941E-08 |  | A | G | -0.008 | 0.006 | 0.195 | 5.09135E-05 | 30.682 |
| TB-BMD | GERD | rs13107325 | 4 | 103188709 | T | C | 0.070 | 0.009 | 2.19938E-14 |  | T | C | 0.014 | 0.012 | 0.255 | 9.68122E-05 | 58.345 |
| TB-BMD | GERD | rs1334297 | 13 | 58335375 | A | G | -0.039 | 0.005 | 1.1413E-12 |  | A | G | 0.014 | 0.007 | 0.032 | 8.39364E-05 | 50.585 |
| TB-BMD | GERD | rs13409451 | 2 | 144257639 | G | A | -0.028 | 0.005 | 1.92943E-08 |  | G | A | -0.001 | 0.006 | 0.817 | 5.23772E-05 | 31.564 |
| TB-BMD | GERD | rs1431196 | 18 | 50832102 | G | A | 0.032 | 0.005 | 2.65461E-11 |  | G | A | -0.001 | 0.006 | 0.919 | 7.37023E-05 | 44.416 |
| TB-BMD | GERD | rs1479405 | 12 | 15387519 | T | C | 0.031 | 0.005 | 9.85213E-10 |  | T | C | 0.014 | 0.006 | 0.020 | 6.19839E-05 | 37.354 |
| TB-BMD | GERD | rs1510719 | 4 | 140938116 | C | T | -0.039 | 0.005 | 3.83619E-15 |  | C | T | -0.001 | 0.006 | 0.880 | 0.000102515 | 61.782 |
| TB-BMD | GERD | rs1592757 | 5 | 103889998 | C | G | 0.031 | 0.005 | 5.9975E-10 |  | C | G | -0.004 | 0.006 | 0.525 | 6.35905E-05 | 38.322 |
| TB-BMD | GERD | rs1596747 | 2 | 193802478 | G | A | 0.031 | 0.005 | 1.00344E-10 |  | G | A | 0.002 | 0.006 | 0.747 | 6.93855E-05 | 41.815 |
| TB-BMD | GERD | rs1716171 | 12 | 123716376 | T | C | 0.038 | 0.006 | 7.82528E-11 |  | T | C | 0.016 | 0.007 | 0.018 | 7.01925E-05 | 42.301 |
| TB-BMD | GERD | rs17379561 | 1 | 98340139 | T | A | 0.053 | 0.007 | 1.07622E-14 |  | T | A | -0.009 | 0.008 | 0.274 | 9.91454E-05 | 59.751 |
| TB-BMD | GERD | rs1883842 | 20 | 41223062 | G | T | 0.031 | 0.005 | 9.26766E-09 |  | G | T | 0.007 | 0.007 | 0.319 | 5.47412E-05 | 32.989 |
| TB-BMD | GERD | rs1937450 | 1 | 66478840 | G | T | 0.032 | 0.005 | 7.06806E-11 |  | G | T | -0.010 | 0.006 | 0.091 | 7.05223E-05 | 42.500 |
| TB-BMD | GERD | rs2016933 | 3 | 65653157 | G | C | -0.031 | 0.005 | 1.04275E-08 |  | G | C | 0.003 | 0.006 | 0.588 | 5.43609E-05 | 32.760 |
| TB-BMD | GERD | rs2023878 | 19 | 18834124 | T | C | -0.036 | 0.006 | 3.03501E-09 |  | T | C | -0.006 | 0.007 | 0.432 | 5.83456E-05 | 35.161 |
| TB-BMD | GERD | rs2043539 | 7 | 12253880 | A | G | 0.027 | 0.005 | 2.24042E-08 |  | A | G | 0.016 | 0.006 | 0.006 | 5.18955E-05 | 31.274 |
| TB-BMD | GERD | rs2106353 | 7 | 126506598 | T | G | 0.037 | 0.006 | 1.37177E-10 |  | T | G | -0.009 | 0.007 | 0.186 | 6.83712E-05 | 41.203 |
| TB-BMD | GERD | rs215614 | 7 | 32347335 | A | G | -0.033 | 0.005 | 4.08413E-11 |  | A | G | 0.003 | 0.006 | 0.577 | 7.23027E-05 | 43.573 |
| TB-BMD | GERD | rs2164300 | 4 | 67813017 | T | C | -0.026 | 0.005 | 4.13352E-08 |  | T | C | 0.005 | 0.006 | 0.410 | 4.99239E-05 | 30.086 |
| TB-BMD | GERD | rs2240326 | 3 | 50128386 | A | G | -0.047 | 0.005 | 1.13214E-22 |  | A | G | -0.012 | 0.006 | 0.043 | 0.000159331 | 96.029 |
| TB-BMD | GERD | rs2396133 | 7 | 109197067 | G | A | 0.029 | 0.005 | 1.10889E-09 |  | G | A | 0.001 | 0.006 | 0.900 | 6.16008E-05 | 37.123 |
| TB-BMD | GERD | rs2396766 | 7 | 114318071 | A | G | 0.032 | 0.005 | 2.33507E-11 |  | A | G | -0.001 | 0.006 | 0.910 | 7.41186E-05 | 44.667 |
| TB-BMD | GERD | rs2734839 | 11 | 113286490 | T | C | -0.028 | 0.005 | 8.78537E-09 |  | T | C | -0.007 | 0.006 | 0.237 | 5.49136E-05 | 33.093 |
| TB-BMD | GERD | rs2744961 | 6 | 34655000 | T | C | 0.029 | 0.005 | 5.81126E-09 |  | T | C | -0.004 | 0.006 | 0.549 | 5.62475E-05 | 33.897 |
| TB-BMD | GERD | rs2782641 | 1 | 44013355 | A | G | 0.027 | 0.005 | 4.32643E-08 |  | A | G | 0.005 | 0.006 | 0.387 | 4.9777E-05 | 29.997 |
| TB-BMD | GERD | rs2815749 | 1 | 72814783 | G | A | 0.039 | 0.006 | 1.07359E-10 |  | G | A | -0.004 | 0.007 | 0.578 | 6.9166E-05 | 41.682 |
| TB-BMD | GERD | rs2834005 | 21 | 34291708 | C | T | 0.030 | 0.005 | 9.42041E-09 |  | C | T | 0.002 | 0.006 | 0.798 | 5.46885E-05 | 32.957 |
| TB-BMD | GERD | rs2838771 | 21 | 46501576 | C | G | -0.028 | 0.005 | 2.90964E-08 |  | C | G | 0.012 | 0.006 | 0.039 | 5.10538E-05 | 30.767 |
| TB-BMD | GERD | rs324769 | 12 | 83969240 | T | C | -0.027 | 0.005 | 3.04775E-08 |  | T | C | 0.000 | 0.006 | 0.978 | 5.09044E-05 | 30.677 |
| TB-BMD | GERD | rs329122 | 5 | 133864599 | A | G | -0.029 | 0.005 | 3.05485E-09 |  | A | G | -0.003 | 0.006 | 0.636 | 5.83244E-05 | 35.148 |
| TB-BMD | GERD | rs3766823 | 1 | 32197257 | A | G | 0.039 | 0.006 | 7.09316E-10 |  | A | G | -0.006 | 0.007 | 0.448 | 6.30472E-05 | 37.995 |
| TB-BMD | GERD | rs3793577 | 9 | 23737627 | G | A | 0.027 | 0.005 | 2.49436E-08 |  | G | A | 0.005 | 0.006 | 0.417 | 5.15497E-05 | 31.066 |
| TB-BMD | GERD | rs3828917 | 6 | 31465917 | T | G | 0.067 | 0.012 | 2.26945E-08 |  | T | G | 0.019 | 0.015 | 0.207 | 5.18541E-05 | 31.249 |
| TB-BMD | GERD | rs3863241 | 8 | 73890335 | T | C | 0.032 | 0.005 | 1.48799E-11 |  | T | C | 0.004 | 0.006 | 0.524 | 7.55829E-05 | 45.550 |
| TB-BMD | GERD | rs4300861 | 2 | 22549441 | T | C | 0.031 | 0.005 | 5.431E-10 |  | T | C | 0.004 | 0.006 | 0.532 | 6.39117E-05 | 38.516 |
| TB-BMD | GERD | rs4382592 | 9 | 134870755 | G | T | -0.030 | 0.005 | 8.19917E-09 |  | G | T | 0.008 | 0.006 | 0.173 | 5.51365E-05 | 33.227 |
| TB-BMD | GERD | rs4713692 | 6 | 33807638 | T | C | -0.028 | 0.005 | 3.06507E-08 |  | T | C | 0.007 | 0.006 | 0.269 | 5.08866E-05 | 30.666 |
| TB-BMD | GERD | rs569356 | 1 | 29136686 | G | A | -0.038 | 0.007 | 4.07005E-08 |  | G | A | -0.012 | 0.009 | 0.166 | 4.99736E-05 | 30.116 |
| TB-BMD | GERD | rs6711584 | 2 | 104421692 | A | G | 0.032 | 0.005 | 2.65583E-11 |  | A | G | 0.001 | 0.006 | 0.864 | 7.37009E-05 | 44.416 |
| TB-BMD | GERD | rs6722661 | 2 | 100806588 | A | G | -0.032 | 0.005 | 1.14623E-10 |  | A | G | -0.006 | 0.006 | 0.298 | 6.89539E-05 | 41.555 |
| TB-BMD | GERD | rs6780459 | 3 | 104624105 | T | A | 0.031 | 0.006 | 3.13993E-08 |  | T | A | -0.014 | 0.007 | 0.036 | 5.08086E-05 | 30.619 |
| TB-BMD | GERD | rs7032155 | 9 | 122672771 | A | C | 0.028 | 0.005 | 1.62686E-08 |  | A | C | 0.001 | 0.006 | 0.933 | 5.29267E-05 | 31.895 |
| TB-BMD | GERD | rs7206608 | 16 | 82872628 | G | C | 0.029 | 0.005 | 1.45761E-08 |  | G | C | -0.006 | 0.006 | 0.301 | 5.32811E-05 | 32.109 |
| TB-BMD | GERD | rs7241572 | 18 | 77580712 | A | G | 0.037 | 0.006 | 9.49336E-10 |  | A | G | 0.004 | 0.007 | 0.562 | 6.21038E-05 | 37.426 |
| TB-BMD | GERD | rs7527682 | 1 | 189172684 | G | A | -0.027 | 0.005 | 3.12831E-08 |  | G | A | -0.002 | 0.006 | 0.745 | 5.08206E-05 | 30.626 |
| TB-BMD | GERD | rs7541875 | 1 | 190957589 | G | A | 0.027 | 0.005 | 1.61213E-08 |  | G | A | -0.001 | 0.006 | 0.845 | 5.29562E-05 | 31.913 |
| TB-BMD | GERD | rs7600261 | 2 | 212622818 | T | C | 0.034 | 0.005 | 9.47327E-11 |  | T | C | 0.004 | 0.006 | 0.580 | 6.95717E-05 | 41.927 |
| TB-BMD | GERD | rs7612999 | 3 | 35678337 | A | G | 0.031 | 0.006 | 4.89824E-08 |  | A | G | -0.004 | 0.007 | 0.513 | 4.93779E-05 | 29.757 |
| TB-BMD | GERD | rs761777 | 10 | 134938075 | G | A | 0.035 | 0.006 | 4.71455E-10 |  | G | A | -0.002 | 0.008 | 0.821 | 6.43697E-05 | 38.792 |
| TB-BMD | GERD | rs7675588 | 4 | 80734978 | A | C | -0.034 | 0.006 | 1.80264E-08 |  | A | C | -0.009 | 0.007 | 0.208 | 5.2596E-05 | 31.696 |
| TB-BMD | GERD | rs7685686 | 4 | 3207142 | G | A | -0.028 | 0.005 | 1.14427E-08 |  | G | A | -0.013 | 0.006 | 0.021 | 5.40613E-05 | 32.579 |
| TB-BMD | GERD | rs773109 | 12 | 56374695 | A | G | -0.038 | 0.005 | 8.71365E-14 |  | A | G | 0.018 | 0.006 | 0.003 | 9.23206E-05 | 55.638 |
| TB-BMD | GERD | rs7942368 | 11 | 76465362 | T | C | -0.034 | 0.006 | 9.54135E-09 |  | T | C | 0.000 | 0.007 | 0.976 | 5.46475E-05 | 32.932 |
| TB-BMD | GERD | rs903678 | 1 | 201809918 | A | G | 0.028 | 0.005 | 4.89091E-08 |  | A | G | 0.003 | 0.006 | 0.660 | 4.93824E-05 | 29.759 |
| TB-BMD | GERD | rs903959 | 8 | 142630782 | A | T | 0.029 | 0.005 | 2.9893E-09 |  | A | T | 0.007 | 0.006 | 0.205 | 5.83945E-05 | 35.191 |
| TB-BMD | GERD | rs9372625 | 6 | 98344031 | A | G | -0.038 | 0.005 | 2.61818E-14 |  | A | G | 0.007 | 0.006 | 0.229 | 9.62435E-05 | 58.002 |
| TB-BMD | GERD | rs9373363 | 6 | 143150043 | G | A | -0.033 | 0.006 | 4.13457E-09 |  | G | A | 0.003 | 0.007 | 0.701 | 5.73464E-05 | 34.559 |
| TB-BMD | GERD | rs9396740 | 6 | 17023108 | A | G | -0.031 | 0.006 | 1.46562E-08 |  | A | G | 0.008 | 0.007 | 0.204 | 5.32631E-05 | 32.098 |
| TB-BMD | GERD | rs942065 | 14 | 94032065 | A | G | 0.031 | 0.005 | 8.44559E-10 |  | A | G | 0.001 | 0.006 | 0.889 | 6.24824E-05 | 37.654 |
| TB-BMD | GERD | rs9517313 | 13 | 99105892 | C | G | 0.033 | 0.005 | 2.04786E-11 |  | C | G | -0.002 | 0.006 | 0.757 | 7.45453E-05 | 44.925 |
| TB-BMD | GERD | rs9529055 | 13 | 66957533 | A | G | 0.027 | 0.005 | 3.10549E-08 |  | A | G | -0.009 | 0.006 | 0.135 | 5.0844E-05 | 30.640 |
| TB-BMD | GERD | rs9542729 | 13 | 31833578 | G | C | -0.036 | 0.006 | 1.41205E-09 |  | G | C | -0.004 | 0.007 | 0.630 | 6.08192E-05 | 36.652 |
| TB-BMD | GERD | rs9615905 | 22 | 48875699 | T | C | 0.028 | 0.005 | 1.21233E-08 |  | T | C | 0.002 | 0.006 | 0.741 | 5.38749E-05 | 32.467 |
| TB-BMD | GERD | rs9636202 | 19 | 18449238 | A | G | -0.035 | 0.005 | 1.51022E-10 |  | A | G | -0.002 | 0.007 | 0.803 | 6.80594E-05 | 41.016 |
| TB-BMD | GERD | rs9940128 | 16 | 53800754 | A | G | 0.033 | 0.005 | 8.05749E-12 |  | A | G | -0.012 | 0.006 | 0.039 | 7.75772E-05 | 46.752 |
| TB-BMD-1 | GERD | rs10010963 | 4 | 159839313 | T | C | -0.027 | 0.005 | 4.91779E-08 |  | T | C | 0.012 | 0.010 | 0.239 | 4.93648E-05 | 29.749 |
| TB-BMD-1 | GERD | rs1011407 | 2 | 60665768 | G | A | -0.042 | 0.007 | 1.09343E-08 |  | G | A | -0.008 | 0.015 | 0.601 | 5.42079E-05 | 32.668 |
| TB-BMD-1 | GERD | rs10133111 | 14 | 103377321 | A | G | 0.042 | 0.007 | 1.35338E-10 |  | A | G | -0.023 | 0.013 | 0.068 | 6.84149E-05 | 41.230 |
| TB-BMD-1 | GERD | rs1021363 | 10 | 106610839 | G | A | -0.031 | 0.005 | 5.09976E-10 |  | G | A | -0.005 | 0.010 | 0.630 | 6.41154E-05 | 38.639 |
| TB-BMD-1 | GERD | rs10837002 | 11 | 38565727 | G | C | 0.028 | 0.005 | 4.02893E-08 |  | G | C | -0.002 | 0.010 | 0.862 | 5.00063E-05 | 30.135 |
| TB-BMD-1 | GERD | rs11762636 | 7 | 2061111 | A | C | -0.051 | 0.006 | 1.88235E-16 |  | A | C | 0.005 | 0.013 | 0.721 | 0.00011237 | 67.722 |
| TB-BMD-1 | GERD | rs11953061 | 5 | 120144025 | T | C | 0.028 | 0.005 | 3.09949E-08 |  | T | C | -0.017 | 0.010 | 0.097 | 5.08505E-05 | 30.644 |
| TB-BMD-1 | GERD | rs12204714 | 6 | 152235339 | T | C | -0.029 | 0.005 | 7.9241E-09 |  | T | C | 0.010 | 0.010 | 0.309 | 5.52467E-05 | 33.294 |
| TB-BMD-1 | GERD | rs12357321 | 10 | 21790476 | A | G | 0.032 | 0.005 | 1.33325E-09 |  | A | G | -0.008 | 0.011 | 0.473 | 6.10048E-05 | 36.764 |
| TB-BMD-1 | GERD | rs12453010 | 17 | 50316131 | T | C | 0.030 | 0.005 | 1.74606E-09 |  | T | C | -0.017 | 0.010 | 0.084 | 6.01325E-05 | 36.238 |
| TB-BMD-1 | GERD | rs12598916 | 16 | 60658751 | G | C | -0.033 | 0.005 | 6.8729E-10 |  | G | C | 0.013 | 0.011 | 0.234 | 6.31493E-05 | 38.056 |
| TB-BMD-1 | GERD | rs12967855 | 18 | 35138245 | G | A | -0.037 | 0.005 | 1.08793E-12 |  | G | A | 0.003 | 0.010 | 0.749 | 8.40922E-05 | 50.678 |
| TB-BMD-1 | GERD | rs12997558 | 2 | 41704580 | A | G | 0.028 | 0.005 | 3.03941E-08 |  | A | G | -0.014 | 0.010 | 0.178 | 5.09135E-05 | 30.682 |
| TB-BMD-1 | GERD | rs13107325 | 4 | 103188709 | T | C | 0.070 | 0.009 | 2.19938E-14 |  | T | C | 0.031 | 0.020 | 0.130 | 9.68122E-05 | 58.345 |
| TB-BMD-1 | GERD | rs1334297 | 13 | 58335375 | A | G | -0.039 | 0.005 | 1.1413E-12 |  | A | G | 0.019 | 0.011 | 0.082 | 8.39364E-05 | 50.585 |
| TB-BMD-1 | GERD | rs13409451 | 2 | 144257639 | G | A | -0.028 | 0.005 | 1.92943E-08 |  | G | A | -0.011 | 0.010 | 0.292 | 5.23772E-05 | 31.564 |
| TB-BMD-1 | GERD | rs1431196 | 18 | 50832102 | G | A | 0.032 | 0.005 | 2.65461E-11 |  | G | A | 0.015 | 0.010 | 0.143 | 7.37023E-05 | 44.416 |
| TB-BMD-1 | GERD | rs1479405 | 12 | 15387519 | T | C | 0.031 | 0.005 | 9.85213E-10 |  | T | C | 0.014 | 0.010 | 0.162 | 6.19839E-05 | 37.354 |
| TB-BMD-1 | GERD | rs1510719 | 4 | 140938116 | C | T | -0.039 | 0.005 | 3.83619E-15 |  | C | T | -0.001 | 0.010 | 0.941 | 0.000102515 | 61.782 |
| TB-BMD-1 | GERD | rs1592757 | 5 | 103889998 | C | G | 0.031 | 0.005 | 5.9975E-10 |  | C | G | -0.012 | 0.011 | 0.248 | 6.35905E-05 | 38.322 |
| TB-BMD-1 | GERD | rs1596747 | 2 | 193802478 | G | A | 0.031 | 0.005 | 1.00344E-10 |  | G | A | -0.010 | 0.010 | 0.329 | 6.93855E-05 | 41.815 |
| TB-BMD-1 | GERD | rs1716171 | 12 | 123716376 | T | C | 0.038 | 0.006 | 7.82528E-11 |  | T | C | 0.006 | 0.012 | 0.609 | 7.01925E-05 | 42.301 |
| TB-BMD-1 | GERD | rs17379561 | 1 | 98340139 | T | A | 0.053 | 0.007 | 1.07622E-14 |  | T | A | 0.002 | 0.014 | 0.884 | 9.91454E-05 | 59.751 |
| TB-BMD-1 | GERD | rs1883842 | 20 | 41223062 | G | T | 0.031 | 0.005 | 9.26766E-09 |  | G | T | -0.012 | 0.011 | 0.287 | 5.47412E-05 | 32.989 |
| TB-BMD-1 | GERD | rs1937450 | 1 | 66478840 | G | T | 0.032 | 0.005 | 7.06806E-11 |  | G | T | 0.000 | 0.010 | 0.967 | 7.05223E-05 | 42.500 |
| TB-BMD-1 | GERD | rs2016933 | 3 | 65653157 | G | C | -0.031 | 0.005 | 1.04275E-08 |  | G | C | 0.001 | 0.011 | 0.931 | 5.43609E-05 | 32.760 |
| TB-BMD-1 | GERD | rs2023878 | 19 | 18834124 | T | C | -0.036 | 0.006 | 3.03501E-09 |  | T | C | -0.015 | 0.012 | 0.233 | 5.83456E-05 | 35.161 |
| TB-BMD-1 | GERD | rs2043539 | 7 | 12253880 | A | G | 0.027 | 0.005 | 2.24042E-08 |  | A | G | 0.016 | 0.010 | 0.094 | 5.18955E-05 | 31.274 |
| TB-BMD-1 | GERD | rs2106353 | 7 | 126506598 | T | G | 0.037 | 0.006 | 1.37177E-10 |  | T | G | 0.009 | 0.012 | 0.468 | 6.83712E-05 | 41.203 |
| TB-BMD-1 | GERD | rs215614 | 7 | 32347335 | A | G | -0.033 | 0.005 | 4.08413E-11 |  | A | G | -0.001 | 0.010 | 0.953 | 7.23027E-05 | 43.573 |
| TB-BMD-1 | GERD | rs2164300 | 4 | 67813017 | T | C | -0.026 | 0.005 | 4.13352E-08 |  | T | C | 0.009 | 0.010 | 0.351 | 4.99239E-05 | 30.086 |
| TB-BMD-1 | GERD | rs2240326 | 3 | 50128386 | A | G | -0.047 | 0.005 | 1.13214E-22 |  | A | G | -0.006 | 0.010 | 0.547 | 0.000159331 | 96.029 |
| TB-BMD-1 | GERD | rs2396133 | 7 | 109197067 | G | A | 0.029 | 0.005 | 1.10889E-09 |  | G | A | 0.005 | 0.010 | 0.587 | 6.16008E-05 | 37.123 |
| TB-BMD-1 | GERD | rs2396766 | 7 | 114318071 | A | G | 0.032 | 0.005 | 2.33507E-11 |  | A | G | 0.006 | 0.010 | 0.570 | 7.41186E-05 | 44.667 |
| TB-BMD-1 | GERD | rs2734839 | 11 | 113286490 | T | C | -0.028 | 0.005 | 8.78537E-09 |  | T | C | -0.016 | 0.010 | 0.112 | 5.49136E-05 | 33.093 |
| TB-BMD-1 | GERD | rs2744961 | 6 | 34655000 | T | C | 0.029 | 0.005 | 5.81126E-09 |  | T | C | -0.003 | 0.010 | 0.764 | 5.62475E-05 | 33.897 |
| TB-BMD-1 | GERD | rs2782641 | 1 | 44013355 | A | G | 0.027 | 0.005 | 4.32643E-08 |  | A | G | 0.003 | 0.010 | 0.778 | 4.9777E-05 | 29.997 |
| TB-BMD-1 | GERD | rs2815749 | 1 | 72814783 | G | A | 0.039 | 0.006 | 1.07359E-10 |  | G | A | 0.000 | 0.012 | 0.990 | 6.9166E-05 | 41.682 |
| TB-BMD-1 | GERD | rs2834005 | 21 | 34291708 | C | T | 0.030 | 0.005 | 9.42041E-09 |  | C | T | 0.006 | 0.010 | 0.537 | 5.46885E-05 | 32.957 |
| TB-BMD-1 | GERD | rs2838771 | 21 | 46501576 | C | G | -0.028 | 0.005 | 2.90964E-08 |  | C | G | 0.009 | 0.010 | 0.403 | 5.10538E-05 | 30.767 |
| TB-BMD-1 | GERD | rs324769 | 12 | 83969240 | T | C | -0.027 | 0.005 | 3.04775E-08 |  | T | C | -0.002 | 0.010 | 0.863 | 5.09044E-05 | 30.677 |
| TB-BMD-1 | GERD | rs329122 | 5 | 133864599 | A | G | -0.029 | 0.005 | 3.05485E-09 |  | A | G | 0.015 | 0.010 | 0.115 | 5.83244E-05 | 35.148 |
| TB-BMD-1 | GERD | rs3766823 | 1 | 32197257 | A | G | 0.039 | 0.006 | 7.09316E-10 |  | A | G | 0.009 | 0.013 | 0.505 | 6.30472E-05 | 37.995 |
| TB-BMD-1 | GERD | rs3793577 | 9 | 23737627 | G | A | 0.027 | 0.005 | 2.49436E-08 |  | G | A | 0.011 | 0.010 | 0.248 | 5.15497E-05 | 31.066 |
| TB-BMD-1 | GERD | rs3828917 | 6 | 31465917 | T | G | 0.067 | 0.012 | 2.26945E-08 |  | T | G | 0.020 | 0.027 | 0.452 | 5.18541E-05 | 31.249 |
| TB-BMD-1 | GERD | rs3863241 | 8 | 73890335 | T | C | 0.032 | 0.005 | 1.48799E-11 |  | T | C | 0.003 | 0.010 | 0.731 | 7.55829E-05 | 45.550 |
| TB-BMD-1 | GERD | rs4300861 | 2 | 22549441 | T | C | 0.031 | 0.005 | 5.431E-10 |  | T | C | -0.002 | 0.010 | 0.834 | 6.39117E-05 | 38.516 |
| TB-BMD-1 | GERD | rs4382592 | 9 | 134870755 | G | T | -0.030 | 0.005 | 8.19917E-09 |  | G | T | 0.020 | 0.011 | 0.059 | 5.51365E-05 | 33.227 |
| TB-BMD-1 | GERD | rs4713692 | 6 | 33807638 | T | C | -0.028 | 0.005 | 3.06507E-08 |  | T | C | -0.012 | 0.010 | 0.262 | 5.08866E-05 | 30.666 |
| TB-BMD-1 | GERD | rs569356 | 1 | 29136686 | G | A | -0.038 | 0.007 | 4.07005E-08 |  | G | A | -0.030 | 0.015 | 0.043 | 4.99736E-05 | 30.116 |
| TB-BMD-1 | GERD | rs6711584 | 2 | 104421692 | A | G | 0.032 | 0.005 | 2.65583E-11 |  | A | G | 0.000 | 0.010 | 0.974 | 7.37009E-05 | 44.416 |
| TB-BMD-1 | GERD | rs6722661 | 2 | 100806588 | A | G | -0.032 | 0.005 | 1.14623E-10 |  | A | G | -0.009 | 0.011 | 0.413 | 6.89539E-05 | 41.555 |
| TB-BMD-1 | GERD | rs6780459 | 3 | 104624105 | T | A | 0.031 | 0.006 | 3.13993E-08 |  | T | A | -0.003 | 0.011 | 0.775 | 5.08086E-05 | 30.619 |
| TB-BMD-1 | GERD | rs7032155 | 9 | 122672771 | A | C | 0.028 | 0.005 | 1.62686E-08 |  | A | C | -0.009 | 0.010 | 0.357 | 5.29267E-05 | 31.895 |
| TB-BMD-1 | GERD | rs7206608 | 16 | 82872628 | G | C | 0.029 | 0.005 | 1.45761E-08 |  | G | C | -0.009 | 0.011 | 0.408 | 5.32811E-05 | 32.109 |
| TB-BMD-1 | GERD | rs7241572 | 18 | 77580712 | A | G | 0.037 | 0.006 | 9.49336E-10 |  | A | G | 0.008 | 0.013 | 0.550 | 6.21038E-05 | 37.426 |
| TB-BMD-1 | GERD | rs7527682 | 1 | 189172684 | G | A | -0.027 | 0.005 | 3.12831E-08 |  | G | A | 0.000 | 0.010 | 0.979 | 5.08206E-05 | 30.626 |
| TB-BMD-1 | GERD | rs7541875 | 1 | 190957589 | G | A | 0.027 | 0.005 | 1.61213E-08 |  | G | A | -0.002 | 0.010 | 0.806 | 5.29562E-05 | 31.913 |
| TB-BMD-1 | GERD | rs7600261 | 2 | 212622818 | T | C | 0.034 | 0.005 | 9.47327E-11 |  | T | C | 0.019 | 0.011 | 0.084 | 6.95717E-05 | 41.927 |
| TB-BMD-1 | GERD | rs7612999 | 3 | 35678337 | A | G | 0.031 | 0.006 | 4.89824E-08 |  | A | G | -0.025 | 0.011 | 0.022 | 4.93779E-05 | 29.757 |
| TB-BMD-1 | GERD | rs761777 | 10 | 134938075 | G | A | 0.035 | 0.006 | 4.71455E-10 |  | G | A | -0.001 | 0.014 | 0.936 | 6.43697E-05 | 38.792 |
| TB-BMD-1 | GERD | rs7675588 | 4 | 80734978 | A | C | -0.034 | 0.006 | 1.80264E-08 |  | A | C | 0.005 | 0.012 | 0.688 | 5.2596E-05 | 31.696 |
| TB-BMD-1 | GERD | rs7685686 | 4 | 3207142 | G | A | -0.028 | 0.005 | 1.14427E-08 |  | G | A | -0.011 | 0.010 | 0.251 | 5.40613E-05 | 32.579 |
| TB-BMD-1 | GERD | rs773109 | 12 | 56374695 | A | G | -0.038 | 0.005 | 8.71365E-14 |  | A | G | 0.015 | 0.011 | 0.151 | 9.23206E-05 | 55.638 |
| TB-BMD-1 | GERD | rs7942368 | 11 | 76465362 | T | C | -0.034 | 0.006 | 9.54135E-09 |  | T | C | -0.001 | 0.011 | 0.950 | 5.46475E-05 | 32.932 |
| TB-BMD-1 | GERD | rs903678 | 1 | 201809918 | A | G | 0.028 | 0.005 | 4.89091E-08 |  | A | G | 0.000 | 0.010 | 0.989 | 4.93824E-05 | 29.759 |
| TB-BMD-1 | GERD | rs903959 | 8 | 142630782 | A | T | 0.029 | 0.005 | 2.9893E-09 |  | A | T | 0.000 | 0.010 | 0.985 | 5.83945E-05 | 35.191 |
| TB-BMD-1 | GERD | rs9372625 | 6 | 98344031 | A | G | -0.038 | 0.005 | 2.61818E-14 |  | A | G | 0.010 | 0.010 | 0.340 | 9.62435E-05 | 58.002 |
| TB-BMD-1 | GERD | rs9373363 | 6 | 143150043 | G | A | -0.033 | 0.006 | 4.13457E-09 |  | G | A | 0.025 | 0.011 | 0.022 | 5.73464E-05 | 34.559 |
| TB-BMD-1 | GERD | rs9396740 | 6 | 17023108 | A | G | -0.031 | 0.006 | 1.46562E-08 |  | A | G | 0.018 | 0.011 | 0.106 | 5.32631E-05 | 32.098 |
| TB-BMD-1 | GERD | rs942065 | 14 | 94032065 | A | G | 0.031 | 0.005 | 8.44559E-10 |  | A | G | 0.003 | 0.010 | 0.761 | 6.24824E-05 | 37.654 |
| TB-BMD-1 | GERD | rs9517313 | 13 | 99105892 | C | G | 0.033 | 0.005 | 2.04786E-11 |  | C | G | -0.004 | 0.010 | 0.684 | 7.45453E-05 | 44.925 |
| TB-BMD-1 | GERD | rs9529055 | 13 | 66957533 | A | G | 0.027 | 0.005 | 3.10549E-08 |  | A | G | -0.020 | 0.010 | 0.042 | 5.0844E-05 | 30.640 |
| TB-BMD-1 | GERD | rs9542729 | 13 | 31833578 | G | C | -0.036 | 0.006 | 1.41205E-09 |  | G | C | 0.002 | 0.012 | 0.846 | 6.08192E-05 | 36.652 |
| TB-BMD-1 | GERD | rs9615905 | 22 | 48875699 | T | C | 0.028 | 0.005 | 1.21233E-08 |  | T | C | 0.004 | 0.010 | 0.681 | 5.38749E-05 | 32.467 |
| TB-BMD-1 | GERD | rs9636202 | 19 | 18449238 | A | G | -0.035 | 0.005 | 1.51022E-10 |  | A | G | -0.024 | 0.011 | 0.032 | 6.80594E-05 | 41.016 |
| TB-BMD-1 | GERD | rs9940128 | 16 | 53800754 | A | G | 0.033 | 0.005 | 8.05749E-12 |  | A | G | -0.007 | 0.010 | 0.477 | 7.75772E-05 | 46.752 |
| TB-BMD-2 | GERD | rs10010963 | 4 | 159839313 | T | C | -0.027 | 0.005 | 4.91779E-08 |  | T | C | -0.011 | 0.011 | 0.337 | 4.93648E-05 | 29.749 |
| TB-BMD-2 | GERD | rs1011407 | 2 | 60665768 | G | A | -0.042 | 0.007 | 1.09343E-08 |  | G | A | -0.005 | 0.016 | 0.764 | 5.42079E-05 | 32.668 |
| TB-BMD-2 | GERD | rs10133111 | 14 | 103377321 | A | G | 0.042 | 0.007 | 1.35338E-10 |  | A | G | 0.021 | 0.014 | 0.143 | 6.84149E-05 | 41.230 |
| TB-BMD-2 | GERD | rs1021363 | 10 | 106610839 | G | A | -0.031 | 0.005 | 5.09976E-10 |  | G | A | 0.008 | 0.011 | 0.489 | 6.41154E-05 | 38.639 |
| TB-BMD-2 | GERD | rs10837002 | 11 | 38565727 | G | C | 0.028 | 0.005 | 4.02893E-08 |  | G | C | 0.011 | 0.011 | 0.309 | 5.00063E-05 | 30.135 |
| TB-BMD-2 | GERD | rs11762636 | 7 | 2061111 | A | C | -0.051 | 0.006 | 1.88235E-16 |  | A | C | -0.009 | 0.014 | 0.492 | 0.00011237 | 67.722 |
| TB-BMD-2 | GERD | rs11953061 | 5 | 120144025 | T | C | 0.028 | 0.005 | 3.09949E-08 |  | T | C | -0.007 | 0.011 | 0.511 | 5.08505E-05 | 30.644 |
| TB-BMD-2 | GERD | rs12204714 | 6 | 152235339 | T | C | -0.029 | 0.005 | 7.9241E-09 |  | T | C | -0.005 | 0.011 | 0.656 | 5.52467E-05 | 33.294 |
| TB-BMD-2 | GERD | rs12357321 | 10 | 21790476 | A | G | 0.032 | 0.005 | 1.33325E-09 |  | A | G | -0.005 | 0.012 | 0.674 | 6.10048E-05 | 36.764 |
| TB-BMD-2 | GERD | rs12453010 | 17 | 50316131 | T | C | 0.030 | 0.005 | 1.74606E-09 |  | T | C | 0.012 | 0.011 | 0.278 | 6.01325E-05 | 36.238 |
| TB-BMD-2 | GERD | rs12598916 | 16 | 60658751 | G | C | -0.033 | 0.005 | 6.8729E-10 |  | G | C | -0.014 | 0.012 | 0.249 | 6.31493E-05 | 38.056 |
| TB-BMD-2 | GERD | rs12967855 | 18 | 35138245 | G | A | -0.037 | 0.005 | 1.08793E-12 |  | G | A | 0.010 | 0.011 | 0.397 | 8.40922E-05 | 50.678 |
| TB-BMD-2 | GERD | rs12997558 | 2 | 41704580 | A | G | 0.028 | 0.005 | 3.03941E-08 |  | A | G | -0.015 | 0.011 | 0.174 | 5.09135E-05 | 30.682 |
| TB-BMD-2 | GERD | rs13107325 | 4 | 103188709 | T | C | 0.070 | 0.009 | 2.19938E-14 |  | T | C | -0.006 | 0.022 | 0.784 | 9.68122E-05 | 58.345 |
| TB-BMD-2 | GERD | rs1334297 | 13 | 58335375 | A | G | -0.039 | 0.005 | 1.1413E-12 |  | A | G | 0.012 | 0.012 | 0.336 | 8.39364E-05 | 50.585 |
| TB-BMD-2 | GERD | rs13409451 | 2 | 144257639 | G | A | -0.028 | 0.005 | 1.92943E-08 |  | G | A | -0.002 | 0.011 | 0.865 | 5.23772E-05 | 31.564 |
| TB-BMD-2 | GERD | rs1431196 | 18 | 50832102 | G | A | 0.032 | 0.005 | 2.65461E-11 |  | G | A | -0.023 | 0.011 | 0.039 | 7.37023E-05 | 44.416 |
| TB-BMD-2 | GERD | rs1479405 | 12 | 15387519 | T | C | 0.031 | 0.005 | 9.85213E-10 |  | T | C | 0.011 | 0.011 | 0.351 | 6.19839E-05 | 37.354 |
| TB-BMD-2 | GERD | rs1510719 | 4 | 140938116 | C | T | -0.039 | 0.005 | 3.83619E-15 |  | C | T | -0.007 | 0.011 | 0.503 | 0.000102515 | 61.782 |
| TB-BMD-2 | GERD | rs1592757 | 5 | 103889998 | C | G | 0.031 | 0.005 | 5.9975E-10 |  | C | G | -0.010 | 0.012 | 0.411 | 6.35905E-05 | 38.322 |
| TB-BMD-2 | GERD | rs1596747 | 2 | 193802478 | G | A | 0.031 | 0.005 | 1.00344E-10 |  | G | A | 0.011 | 0.011 | 0.308 | 6.93855E-05 | 41.815 |
| TB-BMD-2 | GERD | rs1716171 | 12 | 123716376 | T | C | 0.038 | 0.006 | 7.82528E-11 |  | T | C | 0.030 | 0.013 | 0.019 | 7.01925E-05 | 42.301 |
| TB-BMD-2 | GERD | rs17379561 | 1 | 98340139 | T | A | 0.053 | 0.007 | 1.07622E-14 |  | T | A | -0.024 | 0.015 | 0.118 | 9.91454E-05 | 59.751 |
| TB-BMD-2 | GERD | rs1883842 | 20 | 41223062 | G | T | 0.031 | 0.005 | 9.26766E-09 |  | G | T | 0.028 | 0.012 | 0.022 | 5.47412E-05 | 32.989 |
| TB-BMD-2 | GERD | rs1937450 | 1 | 66478840 | G | T | 0.032 | 0.005 | 7.06806E-11 |  | G | T | -0.026 | 0.011 | 0.018 | 7.05223E-05 | 42.500 |
| TB-BMD-2 | GERD | rs2016933 | 3 | 65653157 | G | C | -0.031 | 0.005 | 1.04275E-08 |  | G | C | -0.004 | 0.012 | 0.714 | 5.43609E-05 | 32.760 |
| TB-BMD-2 | GERD | rs2023878 | 19 | 18834124 | T | C | -0.036 | 0.006 | 3.03501E-09 |  | T | C | -0.010 | 0.014 | 0.449 | 5.83456E-05 | 35.161 |
| TB-BMD-2 | GERD | rs2043539 | 7 | 12253880 | A | G | 0.027 | 0.005 | 2.24042E-08 |  | A | G | 0.013 | 0.011 | 0.242 | 5.18955E-05 | 31.274 |
| TB-BMD-2 | GERD | rs2106353 | 7 | 126506598 | T | G | 0.037 | 0.006 | 1.37177E-10 |  | T | G | -0.002 | 0.013 | 0.879 | 6.83712E-05 | 41.203 |
| TB-BMD-2 | GERD | rs215614 | 7 | 32347335 | A | G | -0.033 | 0.005 | 4.08413E-11 |  | A | G | 0.020 | 0.011 | 0.069 | 7.23027E-05 | 43.573 |
| TB-BMD-2 | GERD | rs2164300 | 4 | 67813017 | T | C | -0.026 | 0.005 | 4.13352E-08 |  | T | C | -0.008 | 0.011 | 0.465 | 4.99239E-05 | 30.086 |
| TB-BMD-2 | GERD | rs2240326 | 3 | 50128386 | A | G | -0.047 | 0.005 | 1.13214E-22 |  | A | G | -0.008 | 0.011 | 0.427 | 0.000159331 | 96.029 |
| TB-BMD-2 | GERD | rs2396133 | 7 | 109197067 | G | A | 0.029 | 0.005 | 1.10889E-09 |  | G | A | 0.000 | 0.011 | 0.989 | 6.16008E-05 | 37.123 |
| TB-BMD-2 | GERD | rs2396766 | 7 | 114318071 | A | G | 0.032 | 0.005 | 2.33507E-11 |  | A | G | -0.007 | 0.011 | 0.505 | 7.41186E-05 | 44.667 |
| TB-BMD-2 | GERD | rs2734839 | 11 | 113286490 | T | C | -0.028 | 0.005 | 8.78537E-09 |  | T | C | 0.004 | 0.011 | 0.723 | 5.49136E-05 | 33.093 |
| TB-BMD-2 | GERD | rs2744961 | 6 | 34655000 | T | C | 0.029 | 0.005 | 5.81126E-09 |  | T | C | -0.005 | 0.011 | 0.676 | 5.62475E-05 | 33.897 |
| TB-BMD-2 | GERD | rs2782641 | 1 | 44013355 | A | G | 0.027 | 0.005 | 4.32643E-08 |  | A | G | 0.008 | 0.011 | 0.478 | 4.9777E-05 | 29.997 |
| TB-BMD-2 | GERD | rs2815749 | 1 | 72814783 | G | A | 0.039 | 0.006 | 1.07359E-10 |  | G | A | -0.011 | 0.014 | 0.428 | 6.9166E-05 | 41.682 |
| TB-BMD-2 | GERD | rs2834005 | 21 | 34291708 | C | T | 0.030 | 0.005 | 9.42041E-09 |  | C | T | -0.006 | 0.011 | 0.580 | 5.46885E-05 | 32.957 |
| TB-BMD-2 | GERD | rs2838771 | 21 | 46501576 | C | G | -0.028 | 0.005 | 2.90964E-08 |  | C | G | 0.000 | 0.011 | 0.994 | 5.10538E-05 | 30.767 |
| TB-BMD-2 | GERD | rs324769 | 12 | 83969240 | T | C | -0.027 | 0.005 | 3.04775E-08 |  | T | C | -0.002 | 0.011 | 0.879 | 5.09044E-05 | 30.677 |
| TB-BMD-2 | GERD | rs329122 | 5 | 133864599 | A | G | -0.029 | 0.005 | 3.05485E-09 |  | A | G | -0.001 | 0.011 | 0.939 | 5.83244E-05 | 35.148 |
| TB-BMD-2 | GERD | rs3766823 | 1 | 32197257 | A | G | 0.039 | 0.006 | 7.09316E-10 |  | A | G | -0.026 | 0.014 | 0.065 | 6.30472E-05 | 37.995 |
| TB-BMD-2 | GERD | rs3793577 | 9 | 23737627 | G | A | 0.027 | 0.005 | 2.49436E-08 |  | G | A | 0.006 | 0.011 | 0.554 | 5.15497E-05 | 31.066 |
| TB-BMD-2 | GERD | rs3828917 | 6 | 31465917 | T | G | 0.067 | 0.012 | 2.26945E-08 |  | T | G | 0.024 | 0.028 | 0.392 | 5.18541E-05 | 31.249 |
| TB-BMD-2 | GERD | rs3863241 | 8 | 73890335 | T | C | 0.032 | 0.005 | 1.48799E-11 |  | T | C | 0.006 | 0.011 | 0.591 | 7.55829E-05 | 45.550 |
| TB-BMD-2 | GERD | rs4300861 | 2 | 22549441 | T | C | 0.031 | 0.005 | 5.431E-10 |  | T | C | 0.005 | 0.011 | 0.631 | 6.39117E-05 | 38.516 |
| TB-BMD-2 | GERD | rs4382592 | 9 | 134876940 | G | T | -0.030 | 0.005 | 8.19917E-09 |  | G | T | -0.002 | 0.011 | 0.886 | 5.51365E-05 | 33.227 |
| TB-BMD-2 | GERD | rs4713692 | 6 | 33807638 | T | C | -0.028 | 0.005 | 3.06507E-08 |  | T | C | 0.025 | 0.011 | 0.027 | 5.08866E-05 | 30.666 |
| TB-BMD-2 | GERD | rs569356 | 1 | 29136686 | G | A | -0.038 | 0.007 | 4.07005E-08 |  | G | A | 0.004 | 0.016 | 0.819 | 4.99736E-05 | 30.116 |
| TB-BMD-2 | GERD | rs6711584 | 2 | 104421692 | A | G | 0.032 | 0.005 | 2.65583E-11 |  | A | G | 0.004 | 0.011 | 0.698 | 7.37009E-05 | 44.416 |
| TB-BMD-2 | GERD | rs6722661 | 2 | 100806588 | A | G | -0.032 | 0.005 | 1.14623E-10 |  | A | G | -0.007 | 0.012 | 0.526 | 6.89539E-05 | 41.555 |
| TB-BMD-2 | GERD | rs6780459 | 3 | 104624105 | T | A | 0.031 | 0.006 | 3.13993E-08 |  | T | A | -0.004 | 0.012 | 0.717 | 5.08086E-05 | 30.619 |
| TB-BMD-2 | GERD | rs7032155 | 9 | 122672771 | A | C | 0.028 | 0.005 | 1.62686E-08 |  | A | C | 0.005 | 0.011 | 0.650 | 5.29267E-05 | 31.895 |
| TB-BMD-2 | GERD | rs7206608 | 16 | 82872628 | G | C | 0.029 | 0.005 | 1.45761E-08 |  | G | C | -0.015 | 0.012 | 0.186 | 5.32811E-05 | 32.109 |
| TB-BMD-2 | GERD | rs7241572 | 18 | 77580712 | A | G | 0.037 | 0.006 | 9.49336E-10 |  | A | G | 0.032 | 0.014 | 0.020 | 6.21038E-05 | 37.426 |
| TB-BMD-2 | GERD | rs7527682 | 1 | 189172684 | G | A | -0.027 | 0.005 | 3.12831E-08 |  | G | A | -0.006 | 0.011 | 0.588 | 5.08206E-05 | 30.626 |
| TB-BMD-2 | GERD | rs7541875 | 1 | 190957589 | G | A | 0.027 | 0.005 | 1.61213E-08 |  | G | A | -0.006 | 0.011 | 0.614 | 5.29562E-05 | 31.913 |
| TB-BMD-2 | GERD | rs7600261 | 2 | 212622818 | T | C | 0.034 | 0.005 | 9.47327E-11 |  | T | C | -0.001 | 0.012 | 0.936 | 6.95717E-05 | 41.927 |
| TB-BMD-2 | GERD | rs7612999 | 3 | 35678337 | A | G | 0.031 | 0.006 | 4.89824E-08 |  | A | G | 0.005 | 0.012 | 0.666 | 4.93779E-05 | 29.757 |
| TB-BMD-2 | GERD | rs761777 | 10 | 134938075 | G | A | 0.035 | 0.006 | 4.71455E-10 |  | G | A | -0.014 | 0.014 | 0.321 | 6.43697E-05 | 38.792 |
| TB-BMD-2 | GERD | rs7675588 | 4 | 80734978 | A | C | -0.034 | 0.006 | 1.80264E-08 |  | A | C | -0.008 | 0.013 | 0.555 | 5.2596E-05 | 31.696 |
| TB-BMD-2 | GERD | rs7685686 | 4 | 3207142 | G | A | -0.028 | 0.005 | 1.14427E-08 |  | G | A | -0.020 | 0.011 | 0.061 | 5.40613E-05 | 32.579 |
| TB-BMD-2 | GERD | rs773109 | 12 | 56374695 | A | G | -0.038 | 0.005 | 8.71365E-14 |  | A | G | 0.017 | 0.011 | 0.146 | 9.23206E-05 | 55.638 |
| TB-BMD-2 | GERD | rs7942368 | 11 | 76465362 | T | C | -0.034 | 0.006 | 9.54135E-09 |  | T | C | 0.019 | 0.013 | 0.150 | 5.46475E-05 | 32.932 |
| TB-BMD-2 | GERD | rs903678 | 1 | 201809918 | A | G | 0.028 | 0.005 | 4.89091E-08 |  | A | G | 0.023 | 0.011 | 0.044 | 4.93824E-05 | 29.759 |
| TB-BMD-2 | GERD | rs903959 | 8 | 142630782 | A | T | 0.029 | 0.005 | 2.9893E-09 |  | A | T | 0.004 | 0.011 | 0.721 | 5.83945E-05 | 35.191 |
| TB-BMD-2 | GERD | rs9372625 | 6 | 98344031 | A | G | -0.038 | 0.005 | 2.61818E-14 |  | A | G | -0.005 | 0.011 | 0.643 | 9.62435E-05 | 58.002 |
| TB-BMD-2 | GERD | rs9373363 | 6 | 143150043 | G | A | -0.033 | 0.006 | 4.13457E-09 |  | G | A | -0.014 | 0.012 | 0.255 | 5.73464E-05 | 34.559 |
| TB-BMD-2 | GERD | rs9396740 | 6 | 17023108 | A | G | -0.031 | 0.006 | 1.46562E-08 |  | A | G | 0.011 | 0.012 | 0.366 | 5.32631E-05 | 32.098 |
| TB-BMD-2 | GERD | rs942065 | 14 | 94032065 | A | G | 0.031 | 0.005 | 8.44559E-10 |  | A | G | -0.010 | 0.011 | 0.390 | 6.24824E-05 | 37.654 |
| TB-BMD-2 | GERD | rs9517313 | 13 | 99105892 | C | G | 0.033 | 0.005 | 2.04786E-11 |  | C | G | 0.009 | 0.011 | 0.393 | 7.45453E-05 | 44.925 |
| TB-BMD-2 | GERD | rs9529055 | 13 | 66957533 | A | G | 0.027 | 0.005 | 3.10549E-08 |  | A | G | -0.018 | 0.011 | 0.084 | 5.0844E-05 | 30.640 |
| TB-BMD-2 | GERD | rs9542729 | 13 | 31833578 | G | C | -0.036 | 0.006 | 1.41205E-09 |  | G | C | -0.017 | 0.014 | 0.203 | 6.08192E-05 | 36.652 |
| TB-BMD-2 | GERD | rs9615905 | 22 | 48875699 | T | C | 0.028 | 0.005 | 1.21233E-08 |  | T | C | 0.003 | 0.011 | 0.793 | 5.38749E-05 | 32.467 |
| TB-BMD-2 | GERD | rs9636202 | 19 | 18449238 | A | G | -0.035 | 0.005 | 1.51022E-10 |  | A | G | 0.009 | 0.012 | 0.483 | 6.80594E-05 | 41.016 |
| TB-BMD-2 | GERD | rs9940128 | 16 | 53800754 | A | G | 0.033 | 0.005 | 8.05749E-12 |  | A | G | -0.025 | 0.011 | 0.022 | 7.75772E-05 | 46.752 |
| TB-BMD-3 | GERD | rs10010963 | 4 | 159839313 | T | C | -0.027 | 0.005 | 4.91779E-08 |  | T | C | -0.020 | 0.015 | 0.190 | 4.93648E-05 | 29.749 |
| TB-BMD-3 | GERD | rs1011407 | 2 | 60665768 | G | A | -0.042 | 0.007 | 1.09343E-08 |  | G | A | -0.030 | 0.022 | 0.176 | 5.42079E-05 | 32.668 |
| TB-BMD-3 | GERD | rs10133111 | 14 | 103377321 | A | G | 0.042 | 0.007 | 1.35338E-10 |  | A | G | -0.026 | 0.019 | 0.175 | 6.84149E-05 | 41.230 |
| TB-BMD-3 | GERD | rs1021363 | 10 | 106610839 | G | A | -0.031 | 0.005 | 5.09976E-10 |  | G | A | -0.018 | 0.016 | 0.240 | 6.41154E-05 | 38.639 |
| TB-BMD-3 | GERD | rs10837002 | 11 | 38565727 | G | C | 0.028 | 0.005 | 4.02893E-08 |  | G | C | -0.007 | 0.016 | 0.672 | 5.00063E-05 | 30.135 |
| TB-BMD-3 | GERD | rs11762636 | 7 | 2061111 | A | C | -0.051 | 0.006 | 1.88235E-16 |  | A | C | -0.015 | 0.019 | 0.440 | 0.00011237 | 67.722 |
| TB-BMD-3 | GERD | rs11953061 | 5 | 120144025 | T | C | 0.028 | 0.005 | 3.09949E-08 |  | T | C | -0.008 | 0.016 | 0.605 | 5.08505E-05 | 30.644 |
| TB-BMD-3 | GERD | rs12204714 | 6 | 152235339 | T | C | -0.029 | 0.005 | 7.9241E-09 |  | T | C | -0.005 | 0.015 | 0.753 | 5.52467E-05 | 33.294 |
| TB-BMD-3 | GERD | rs12357321 | 10 | 21790476 | A | G | 0.032 | 0.005 | 1.33325E-09 |  | A | G | 0.025 | 0.016 | 0.116 | 6.10048E-05 | 36.764 |
| TB-BMD-3 | GERD | rs12453010 | 17 | 50316131 | T | C | 0.030 | 0.005 | 1.74606E-09 |  | T | C | -0.013 | 0.015 | 0.391 | 6.01325E-05 | 36.238 |
| TB-BMD-3 | GERD | rs12598916 | 16 | 60658751 | G | C | -0.033 | 0.005 | 6.8729E-10 |  | G | C | 0.013 | 0.017 | 0.425 | 6.31493E-05 | 38.056 |
| TB-BMD-3 | GERD | rs12967855 | 18 | 35138245 | G | A | -0.037 | 0.005 | 1.08793E-12 |  | G | A | -0.001 | 0.015 | 0.958 | 8.40922E-05 | 50.678 |
| TB-BMD-3 | GERD | rs12997558 | 2 | 41704580 | A | G | 0.028 | 0.005 | 3.03941E-08 |  | A | G | -0.006 | 0.015 | 0.689 | 5.09135E-05 | 30.682 |
| TB-BMD-3 | GERD | rs13107325 | 4 | 103188709 | T | C | 0.070 | 0.009 | 2.19938E-14 |  | T | C | 0.003 | 0.030 | 0.919 | 9.68122E-05 | 58.345 |
| TB-BMD-3 | GERD | rs1334297 | 13 | 58335375 | A | G | -0.039 | 0.005 | 1.1413E-12 |  | A | G | 0.023 | 0.017 | 0.175 | 8.39364E-05 | 50.585 |
| TB-BMD-3 | GERD | rs13409451 | 2 | 144257639 | G | A | -0.028 | 0.005 | 1.92943E-08 |  | G | A | -0.026 | 0.015 | 0.095 | 5.23772E-05 | 31.564 |
| TB-BMD-3 | GERD | rs1431196 | 18 | 50832102 | G | A | 0.032 | 0.005 | 2.65461E-11 |  | G | A | -0.017 | 0.015 | 0.276 | 7.37023E-05 | 44.416 |
| TB-BMD-3 | GERD | rs1479405 | 12 | 15387519 | T | C | 0.031 | 0.005 | 9.85213E-10 |  | T | C | -0.003 | 0.016 | 0.860 | 6.19839E-05 | 37.354 |
| TB-BMD-3 | GERD | rs1510719 | 4 | 140938116 | C | T | -0.039 | 0.005 | 3.83619E-15 |  | C | T | 0.012 | 0.015 | 0.425 | 0.000102515 | 61.782 |
| TB-BMD-3 | GERD | rs1592757 | 5 | 103889998 | C | G | 0.031 | 0.005 | 5.9975E-10 |  | C | G | 0.011 | 0.016 | 0.481 | 6.35905E-05 | 38.322 |
| TB-BMD-3 | GERD | rs1596747 | 2 | 193802478 | G | A | 0.031 | 0.005 | 1.00344E-10 |  | G | A | -0.022 | 0.015 | 0.146 | 6.93855E-05 | 41.815 |
| TB-BMD-3 | GERD | rs1716171 | 12 | 123716376 | T | C | 0.038 | 0.006 | 7.82528E-11 |  | T | C | 0.007 | 0.018 | 0.695 | 7.01925E-05 | 42.301 |
| TB-BMD-3 | GERD | rs17379561 | 1 | 98340139 | T | A | 0.053 | 0.007 | 1.07622E-14 |  | T | A | -0.017 | 0.022 | 0.444 | 9.91454E-05 | 59.751 |
| TB-BMD-3 | GERD | rs1883842 | 20 | 41223062 | G | T | 0.031 | 0.005 | 9.26766E-09 |  | G | T | 0.016 | 0.017 | 0.350 | 5.47412E-05 | 32.989 |
| TB-BMD-3 | GERD | rs1937450 | 1 | 66478840 | G | T | 0.032 | 0.005 | 7.06806E-11 |  | G | T | -0.011 | 0.015 | 0.473 | 7.05223E-05 | 42.500 |
| TB-BMD-3 | GERD | rs2016933 | 3 | 65653157 | G | C | -0.031 | 0.005 | 1.04275E-08 |  | G | C | -0.007 | 0.016 | 0.646 | 5.43609E-05 | 32.760 |
| TB-BMD-3 | GERD | rs2023878 | 19 | 18834124 | T | C | -0.036 | 0.006 | 3.03501E-09 |  | T | C | 0.020 | 0.018 | 0.266 | 5.83456E-05 | 35.161 |
| TB-BMD-3 | GERD | rs2043539 | 7 | 12253880 | A | G | 0.027 | 0.005 | 2.24042E-08 |  | A | G | -0.007 | 0.015 | 0.631 | 5.18955E-05 | 31.274 |
| TB-BMD-3 | GERD | rs2106353 | 7 | 126506598 | T | G | 0.037 | 0.006 | 1.37177E-10 |  | T | G | -0.038 | 0.018 | 0.033 | 6.83712E-05 | 41.203 |
| TB-BMD-3 | GERD | rs215614 | 7 | 32347335 | A | G | -0.033 | 0.005 | 4.08413E-11 |  | A | G | 0.000 | 0.015 | 0.995 | 7.23027E-05 | 43.573 |
| TB-BMD-3 | GERD | rs2164300 | 4 | 67813017 | T | C | -0.026 | 0.005 | 4.13352E-08 |  | T | C | -0.010 | 0.015 | 0.498 | 4.99239E-05 | 30.086 |
| TB-BMD-3 | GERD | rs2240326 | 3 | 50128386 | A | G | -0.047 | 0.005 | 1.13214E-22 |  | A | G | -0.041 | 0.015 | 0.005 | 0.000159331 | 96.029 |
| TB-BMD-3 | GERD | rs2396133 | 7 | 109197067 | G | A | 0.029 | 0.005 | 1.10889E-09 |  | G | A | -0.007 | 0.015 | 0.659 | 6.16008E-05 | 37.123 |
| TB-BMD-3 | GERD | rs2396766 | 7 | 114318071 | A | G | 0.032 | 0.005 | 2.33507E-11 |  | A | G | 0.004 | 0.015 | 0.787 | 7.41186E-05 | 44.667 |
| TB-BMD-3 | GERD | rs2734839 | 11 | 113286490 | T | C | -0.028 | 0.005 | 8.78537E-09 |  | T | C | -0.016 | 0.015 | 0.276 | 5.49136E-05 | 33.093 |
| TB-BMD-3 | GERD | rs2744961 | 6 | 34655000 | T | C | 0.029 | 0.005 | 5.81126E-09 |  | T | C | 0.003 | 0.016 | 0.875 | 5.62475E-05 | 33.897 |
| TB-BMD-3 | GERD | rs2782641 | 1 | 44013355 | A | G | 0.027 | 0.005 | 4.32643E-08 |  | A | G | 0.010 | 0.015 | 0.536 | 4.9777E-05 | 29.997 |
| TB-BMD-3 | GERD | rs2815749 | 1 | 72814783 | G | A | 0.039 | 0.006 | 1.07359E-10 |  | G | A | 0.017 | 0.019 | 0.371 | 6.9166E-05 | 41.682 |
| TB-BMD-3 | GERD | rs2834005 | 21 | 34291708 | C | T | 0.030 | 0.005 | 9.42041E-09 |  | C | T | -0.007 | 0.016 | 0.679 | 5.46885E-05 | 32.957 |
| TB-BMD-3 | GERD | rs2838771 | 21 | 46501576 | C | G | -0.028 | 0.005 | 2.90964E-08 |  | C | G | 0.015 | 0.016 | 0.345 | 5.10538E-05 | 30.767 |
| TB-BMD-3 | GERD | rs324769 | 12 | 83969240 | T | C | -0.027 | 0.005 | 3.04775E-08 |  | T | C | 0.003 | 0.015 | 0.843 | 5.09044E-05 | 30.677 |
| TB-BMD-3 | GERD | rs329122 | 5 | 133864599 | A | G | -0.029 | 0.005 | 3.05485E-09 |  | A | G | -0.018 | 0.015 | 0.221 | 5.83244E-05 | 35.148 |
| TB-BMD-3 | GERD | rs3766823 | 1 | 32197257 | A | G | 0.039 | 0.006 | 7.09316E-10 |  | A | G | -0.052 | 0.019 | 0.005 | 6.30472E-05 | 37.995 |
| TB-BMD-3 | GERD | rs3793577 | 9 | 23737627 | G | A | 0.027 | 0.005 | 2.49436E-08 |  | G | A | -0.004 | 0.015 | 0.768 | 5.15497E-05 | 31.066 |
| TB-BMD-3 | GERD | rs3828917 | 6 | 31465917 | T | G | 0.067 | 0.012 | 2.26945E-08 |  | T | G | -0.014 | 0.038 | 0.711 | 5.18541E-05 | 31.249 |
| TB-BMD-3 | GERD | rs3863241 | 8 | 73890335 | T | C | 0.032 | 0.005 | 1.48799E-11 |  | T | C | -0.019 | 0.015 | 0.205 | 7.55829E-05 | 45.550 |
| TB-BMD-3 | GERD | rs4300861 | 2 | 22549441 | T | C | 0.031 | 0.005 | 5.431E-10 |  | T | C | 0.024 | 0.016 | 0.122 | 6.39117E-05 | 38.516 |
| TB-BMD-3 | GERD | rs4382592 | 9 | 134870755 | G | T | -0.030 | 0.005 | 8.19917E-09 |  | G | T | 0.000 | 0.016 | 0.978 | 5.51365E-05 | 33.227 |
| TB-BMD-3 | GERD | rs4713692 | 6 | 33807638 | T | C | -0.028 | 0.005 | 3.06507E-08 |  | T | C | 0.025 | 0.016 | 0.104 | 5.08866E-05 | 30.666 |
| TB-BMD-3 | GERD | rs569356 | 1 | 29136686 | G | A | -0.038 | 0.007 | 4.07005E-08 |  | G | A | 0.001 | 0.023 | 0.974 | 4.99736E-05 | 30.116 |
| TB-BMD-3 | GERD | rs6711584 | 2 | 104421692 | A | G | 0.032 | 0.005 | 2.65583E-11 |  | A | G | -0.014 | 0.015 | 0.357 | 7.37009E-05 | 44.416 |
| TB-BMD-3 | GERD | rs6722661 | 2 | 100806588 | A | G | -0.032 | 0.005 | 1.14623E-10 |  | A | G | 0.003 | 0.016 | 0.843 | 6.89539E-05 | 41.555 |
| TB-BMD-3 | GERD | rs6780459 | 3 | 104624105 | T | A | 0.031 | 0.006 | 3.13993E-08 |  | T | A | -0.013 | 0.017 | 0.435 | 5.08086E-05 | 30.619 |
| TB-BMD-3 | GERD | rs7032155 | 9 | 122672771 | A | C | 0.028 | 0.005 | 1.62686E-08 |  | A | C | 0.000 | 0.015 | 0.992 | 5.29267E-05 | 31.895 |
| TB-BMD-3 | GERD | rs7206608 | 16 | 82872628 | G | C | 0.029 | 0.005 | 1.45761E-08 |  | G | C | 0.017 | 0.016 | 0.283 | 5.32811E-05 | 32.109 |
| TB-BMD-3 | GERD | rs7241572 | 18 | 77580712 | A | G | 0.037 | 0.006 | 9.49336E-10 |  | A | G | -0.038 | 0.019 | 0.044 | 6.21038E-05 | 37.426 |
| TB-BMD-3 | GERD | rs7527682 | 1 | 189172684 | G | A | -0.027 | 0.005 | 3.12831E-08 |  | G | A | -0.014 | 0.015 | 0.348 | 5.08206E-05 | 30.626 |
| TB-BMD-3 | GERD | rs7541875 | 1 | 190957589 | G | A | 0.027 | 0.005 | 1.61213E-08 |  | G | A | 0.002 | 0.015 | 0.918 | 5.29562E-05 | 31.913 |
| TB-BMD-3 | GERD | rs7600261 | 2 | 212622818 | T | C | 0.034 | 0.005 | 9.47327E-11 |  | T | C | -0.018 | 0.017 | 0.278 | 6.95717E-05 | 41.927 |
| TB-BMD-3 | GERD | rs7612999 | 3 | 35678337 | A | G | 0.031 | 0.006 | 4.89824E-08 |  | A | G | -0.012 | 0.017 | 0.466 | 4.93779E-05 | 29.757 |
| TB-BMD-3 | GERD | rs761777 | 10 | 134938075 | G | A | 0.035 | 0.006 | 4.71455E-10 |  | G | A | 0.015 | 0.019 | 0.438 | 6.43697E-05 | 38.792 |
| TB-BMD-3 | GERD | rs7675588 | 4 | 80734978 | A | C | -0.034 | 0.006 | 1.80264E-08 |  | A | C | -0.024 | 0.018 | 0.171 | 5.2596E-05 | 31.696 |
| TB-BMD-3 | GERD | rs7685686 | 4 | 3207142 | G | A | -0.028 | 0.005 | 1.14427E-08 |  | G | A | -0.017 | 0.015 | 0.246 | 5.40613E-05 | 32.579 |
| TB-BMD-3 | GERD | rs773109 | 12 | 56374695 | A | G | -0.038 | 0.005 | 8.71365E-14 |  | A | G | 0.013 | 0.016 | 0.405 | 9.23206E-05 | 55.638 |
| TB-BMD-3 | GERD | rs7942368 | 11 | 76465362 | T | C | -0.034 | 0.006 | 9.54135E-09 |  | T | C | -0.006 | 0.017 | 0.744 | 5.46475E-05 | 32.932 |
| TB-BMD-3 | GERD | rs903678 | 1 | 201809918 | A | G | 0.028 | 0.005 | 4.89091E-08 |  | A | G | -0.012 | 0.016 | 0.457 | 4.93824E-05 | 29.759 |
| TB-BMD-3 | GERD | rs903959 | 8 | 142630782 | A | T | 0.029 | 0.005 | 2.9893E-09 |  | A | T | 0.014 | 0.015 | 0.362 | 5.83945E-05 | 35.191 |
| TB-BMD-3 | GERD | rs9372625 | 6 | 98344031 | A | G | -0.038 | 0.005 | 2.61818E-14 |  | A | G | 0.000 | 0.016 | 0.990 | 9.62435E-05 | 58.002 |
| TB-BMD-3 | GERD | rs9373363 | 6 | 143150043 | G | A | -0.033 | 0.006 | 4.13457E-09 |  | G | A | -0.007 | 0.017 | 0.668 | 5.73464E-05 | 34.559 |
| TB-BMD-3 | GERD | rs9396740 | 6 | 17023108 | A | G | -0.031 | 0.006 | 1.46562E-08 |  | A | G | -0.007 | 0.017 | 0.665 | 5.32631E-05 | 32.098 |
| TB-BMD-3 | GERD | rs942065 | 14 | 94032065 | A | G | 0.031 | 0.005 | 8.44559E-10 |  | A | G | 0.004 | 0.016 | 0.810 | 6.24824E-05 | 37.654 |
| TB-BMD-3 | GERD | rs9517313 | 13 | 99105892 | C | G | 0.033 | 0.005 | 2.04786E-11 |  | C | G | -0.007 | 0.015 | 0.658 | 7.45453E-05 | 44.925 |
| TB-BMD-3 | GERD | rs9529055 | 13 | 66957533 | A | G | 0.027 | 0.005 | 3.10549E-08 |  | A | G | -0.014 | 0.015 | 0.333 | 5.0844E-05 | 30.640 |
| TB-BMD-3 | GERD | rs9542729 | 13 | 31833578 | G | C | -0.036 | 0.006 | 1.41205E-09 |  | G | C | 0.016 | 0.019 | 0.406 | 6.08192E-05 | 36.652 |
| TB-BMD-3 | GERD | rs9615905 | 22 | 48875699 | T | C | 0.028 | 0.005 | 1.21233E-08 |  | T | C | 0.017 | 0.015 | 0.264 | 5.38749E-05 | 32.467 |
| TB-BMD-3 | GERD | rs9636202 | 19 | 18449238 | A | G | -0.035 | 0.005 | 1.51022E-10 |  | A | G | 0.006 | 0.017 | 0.730 | 6.80594E-05 | 41.016 |
| TB-BMD-3 | GERD | rs9940128 | 16 | 53800754 | A | G | 0.033 | 0.005 | 8.05749E-12 |  | A | G | -0.010 | 0.015 | 0.514 | 7.75772E-05 | 46.752 |
| TB-BMD-4 | GERD | rs10010963 | 4 | 159839313 | T | C | -0.027 | 0.005 | 4.91779E-08 |  | T | C | -0.027 | 0.024 | 0.263 | 4.93648E-05 | 29.749 |
| TB-BMD-4 | GERD | rs1011407 | 2 | 60665768 | G | A | -0.042 | 0.007 | 1.09343E-08 |  | G | A | -0.024 | 0.035 | 0.493 | 5.42079E-05 | 32.668 |
| TB-BMD-4 | GERD | rs10133111 | 14 | 103377321 | A | G | 0.042 | 0.007 | 1.35338E-10 |  | A | G | 0.014 | 0.029 | 0.619 | 6.84149E-05 | 41.230 |
| TB-BMD-4 | GERD | rs1021363 | 10 | 106610839 | G | A | -0.031 | 0.005 | 5.09976E-10 |  | G | A | -0.003 | 0.024 | 0.895 | 6.41154E-05 | 38.639 |
| TB-BMD-4 | GERD | rs10837002 | 11 | 38565727 | G | C | 0.028 | 0.005 | 4.02893E-08 |  | G | C | -0.014 | 0.024 | 0.564 | 5.00063E-05 | 30.135 |
| TB-BMD-4 | GERD | rs11762636 | 7 | 2061111 | A | C | -0.051 | 0.006 | 1.88235E-16 |  | A | C | -0.012 | 0.029 | 0.691 | 0.00011237 | 67.722 |
| TB-BMD-4 | GERD | rs11953061 | 5 | 120144025 | T | C | 0.028 | 0.005 | 3.09949E-08 |  | T | C | 0.009 | 0.024 | 0.720 | 5.08505E-05 | 30.644 |
| TB-BMD-4 | GERD | rs12204714 | 6 | 152235339 | T | C | -0.029 | 0.005 | 7.9241E-09 |  | T | C | 0.001 | 0.024 | 0.972 | 5.52467E-05 | 33.294 |
| TB-BMD-4 | GERD | rs12357321 | 10 | 21790476 | A | G | 0.032 | 0.005 | 1.33325E-09 |  | A | G | 0.021 | 0.025 | 0.402 | 6.10048E-05 | 36.764 |
| TB-BMD-4 | GERD | rs12453010 | 17 | 50316131 | T | C | 0.030 | 0.005 | 1.74606E-09 |  | T | C | -0.002 | 0.023 | 0.931 | 6.01325E-05 | 36.238 |
| TB-BMD-4 | GERD | rs12598916 | 16 | 60658751 | G | C | -0.033 | 0.005 | 6.8729E-10 |  | G | C | -0.025 | 0.025 | 0.324 | 6.31493E-05 | 38.056 |
| TB-BMD-4 | GERD | rs12967855 | 18 | 35138245 | G | A | -0.037 | 0.005 | 1.08793E-12 |  | G | A | -0.038 | 0.024 | 0.112 | 8.40922E-05 | 50.678 |
| TB-BMD-4 | GERD | rs12997558 | 2 | 41704580 | A | G | 0.028 | 0.005 | 3.03941E-08 |  | A | G | 0.033 | 0.024 | 0.166 | 5.09135E-05 | 30.682 |
| TB-BMD-4 | GERD | rs13107325 | 4 | 103188709 | T | C | 0.070 | 0.009 | 2.19938E-14 |  | T | C | 0.007 | 0.051 | 0.890 | 9.68122E-05 | 58.345 |
| TB-BMD-4 | GERD | rs1334297 | 13 | 58335375 | A | G | -0.039 | 0.005 | 1.1413E-12 |  | A | G | 0.022 | 0.026 | 0.386 | 8.39364E-05 | 50.585 |
| TB-BMD-4 | GERD | rs13409451 | 2 | 144257639 | G | A | -0.028 | 0.005 | 1.92943E-08 |  | G | A | 0.029 | 0.024 | 0.217 | 5.23772E-05 | 31.564 |
| TB-BMD-4 | GERD | rs1431196 | 18 | 50832102 | G | A | 0.032 | 0.005 | 2.65461E-11 |  | G | A | -0.008 | 0.023 | 0.742 | 7.37023E-05 | 44.416 |
| TB-BMD-4 | GERD | rs1479405 | 12 | 15387519 | T | C | 0.031 | 0.005 | 9.85213E-10 |  | T | C | -0.018 | 0.024 | 0.457 | 6.19839E-05 | 37.354 |
| TB-BMD-4 | GERD | rs1510719 | 4 | 140938116 | C | T | -0.039 | 0.005 | 3.83619E-15 |  | C | T | 0.011 | 0.024 | 0.660 | 0.000102515 | 61.782 |
| TB-BMD-4 | GERD | rs1592757 | 5 | 103889998 | C | G | 0.031 | 0.005 | 5.9975E-10 |  | C | G | 0.042 | 0.025 | 0.091 | 6.35905E-05 | 38.322 |
| TB-BMD-4 | GERD | rs1596747 | 2 | 193802478 | G | A | 0.031 | 0.005 | 1.00344E-10 |  | G | A | 0.003 | 0.024 | 0.904 | 6.93855E-05 | 41.815 |
| TB-BMD-4 | GERD | rs1716171 | 12 | 123716376 | T | C | 0.038 | 0.006 | 7.82528E-11 |  | T | C | -0.022 | 0.027 | 0.412 | 7.01925E-05 | 42.301 |
| TB-BMD-4 | GERD | rs17379561 | 1 | 98340139 | T | A | 0.053 | 0.007 | 1.07622E-14 |  | T | A | -0.047 | 0.033 | 0.159 | 9.91454E-05 | 59.751 |
| TB-BMD-4 | GERD | rs1883842 | 20 | 41223062 | G | T | 0.031 | 0.005 | 9.26766E-09 |  | G | T | -0.046 | 0.026 | 0.083 | 5.47412E-05 | 32.989 |
| TB-BMD-4 | GERD | rs1937450 | 1 | 66478840 | G | T | 0.032 | 0.005 | 7.06806E-11 |  | G | T | 0.016 | 0.023 | 0.476 | 7.05223E-05 | 42.500 |
| TB-BMD-4 | GERD | rs2016933 | 3 | 65653157 | G | C | -0.031 | 0.005 | 1.04275E-08 |  | G | C | 0.002 | 0.025 | 0.928 | 5.43609E-05 | 32.760 |
| TB-BMD-4 | GERD | rs2023878 | 19 | 18834124 | T | C | -0.036 | 0.006 | 3.03501E-09 |  | T | C | -0.007 | 0.029 | 0.812 | 5.83456E-05 | 35.161 |
| TB-BMD-4 | GERD | rs2043539 | 7 | 12253880 | A | G | 0.027 | 0.005 | 2.24042E-08 |  | A | G | 0.015 | 0.023 | 0.533 | 5.18955E-05 | 31.274 |
| TB-BMD-4 | GERD | rs2106353 | 7 | 126506598 | T | G | 0.037 | 0.006 | 1.37177E-10 |  | T | G | -0.042 | 0.028 | 0.134 | 6.83712E-05 | 41.203 |
| TB-BMD-4 | GERD | rs215614 | 7 | 32347335 | A | G | -0.033 | 0.005 | 4.08413E-11 |  | A | G | -0.001 | 0.024 | 0.964 | 7.23027E-05 | 43.573 |
| TB-BMD-4 | GERD | rs2164300 | 4 | 67813017 | T | C | -0.026 | 0.005 | 4.13352E-08 |  | T | C | 0.024 | 0.023 | 0.306 | 4.99239E-05 | 30.086 |
| TB-BMD-4 | GERD | rs2240326 | 3 | 50128386 | A | G | -0.047 | 0.005 | 1.13214E-22 |  | A | G | 0.007 | 0.023 | 0.748 | 0.000159331 | 96.029 |
| TB-BMD-4 | GERD | rs2396133 | 7 | 109197067 | G | A | 0.029 | 0.005 | 1.10889E-09 |  | G | A | -0.007 | 0.023 | 0.746 | 6.16008E-05 | 37.123 |
| TB-BMD-4 | GERD | rs2396766 | 7 | 114318071 | A | G | 0.032 | 0.005 | 2.33507E-11 |  | A | G | -0.008 | 0.023 | 0.722 | 7.41186E-05 | 44.667 |
| TB-BMD-4 | GERD | rs2734839 | 11 | 113286490 | T | C | -0.028 | 0.005 | 8.78537E-09 |  | T | C | -0.004 | 0.023 | 0.856 | 5.49136E-05 | 33.093 |
| TB-BMD-4 | GERD | rs2744961 | 6 | 34655000 | T | C | 0.029 | 0.005 | 5.81126E-09 |  | T | C | 0.003 | 0.025 | 0.920 | 5.62475E-05 | 33.897 |
| TB-BMD-4 | GERD | rs2782641 | 1 | 44013355 | A | G | 0.027 | 0.005 | 4.32643E-08 |  | A | G | 0.001 | 0.024 | 0.971 | 4.9777E-05 | 29.997 |
| TB-BMD-4 | GERD | rs2815749 | 1 | 72814783 | G | A | 0.039 | 0.006 | 1.07359E-10 |  | G | A | -0.031 | 0.028 | 0.278 | 6.9166E-05 | 41.682 |
| TB-BMD-4 | GERD | rs2834005 | 21 | 34291708 | C | T | 0.030 | 0.005 | 9.42041E-09 |  | C | T | 0.009 | 0.025 | 0.719 | 5.46885E-05 | 32.957 |
| TB-BMD-4 | GERD | rs2838771 | 21 | 46501576 | C | G | -0.028 | 0.005 | 2.90964E-08 |  | C | G | 0.021 | 0.024 | 0.389 | 5.10538E-05 | 30.767 |
| TB-BMD-4 | GERD | rs324769 | 12 | 83969240 | T | C | -0.027 | 0.005 | 3.04775E-08 |  | T | C | 0.023 | 0.023 | 0.314 | 5.09044E-05 | 30.677 |
| TB-BMD-4 | GERD | rs329122 | 5 | 133864599 | A | G | -0.029 | 0.005 | 3.05485E-09 |  | A | G | -0.029 | 0.023 | 0.199 | 5.83244E-05 | 35.148 |
| TB-BMD-4 | GERD | rs3766823 | 1 | 32197257 | A | G | 0.039 | 0.006 | 7.09316E-10 |  | A | G | -0.033 | 0.030 | 0.270 | 6.30472E-05 | 37.995 |
| TB-BMD-4 | GERD | rs3793577 | 9 | 23737627 | G | A | 0.027 | 0.005 | 2.49436E-08 |  | G | A | -0.004 | 0.023 | 0.845 | 5.15497E-05 | 31.066 |
| TB-BMD-4 | GERD | rs3828917 | 6 | 31465917 | T | G | 0.067 | 0.012 | 2.26945E-08 |  | T | G | 0.054 | 0.064 | 0.400 | 5.18541E-05 | 31.249 |
| TB-BMD-4 | GERD | rs3863241 | 8 | 73890335 | T | C | 0.032 | 0.005 | 1.48799E-11 |  | T | C | -0.013 | 0.023 | 0.573 | 7.55829E-05 | 45.550 |
| TB-BMD-4 | GERD | rs4300861 | 2 | 22549441 | T | C | 0.031 | 0.005 | 5.431E-10 |  | T | C | 0.014 | 0.024 | 0.548 | 6.39117E-05 | 38.516 |
| TB-BMD-4 | GERD | rs4382592 | 9 | 134870755 | G | T | -0.030 | 0.005 | 8.19917E-09 |  | G | T | 0.025 | 0.024 | 0.312 | 5.51365E-05 | 33.227 |
| TB-BMD-4 | GERD | rs4713692 | 6 | 33807638 | T | C | -0.028 | 0.005 | 3.06507E-08 |  | T | C | 0.038 | 0.025 | 0.122 | 5.08866E-05 | 30.666 |
| TB-BMD-4 | GERD | rs569356 | 1 | 29136686 | G | A | -0.038 | 0.007 | 4.07005E-08 |  | G | A | 0.010 | 0.034 | 0.766 | 4.99736E-05 | 30.116 |
| TB-BMD-4 | GERD | rs6711584 | 2 | 104421692 | A | G | 0.032 | 0.005 | 2.65583E-11 |  | A | G | -0.010 | 0.023 | 0.649 | 7.37009E-05 | 44.416 |
| TB-BMD-4 | GERD | rs6722661 | 2 | 100806588 | A | G | -0.032 | 0.005 | 1.14623E-10 |  | A | G | 0.031 | 0.025 | 0.210 | 6.89539E-05 | 41.555 |
| TB-BMD-4 | GERD | rs6780459 | 3 | 104624105 | T | A | 0.031 | 0.006 | 3.13993E-08 |  | T | A | -0.032 | 0.025 | 0.206 | 5.08086E-05 | 30.619 |
| TB-BMD-4 | GERD | rs7032155 | 9 | 122672771 | A | C | 0.028 | 0.005 | 1.62686E-08 |  | A | C | 0.000 | 0.023 | 0.990 | 5.29267E-05 | 31.895 |
| TB-BMD-4 | GERD | rs7206608 | 16 | 82872628 | G | C | 0.029 | 0.005 | 1.45761E-08 |  | G | C | 0.020 | 0.024 | 0.407 | 5.32811E-05 | 32.109 |
| TB-BMD-4 | GERD | rs7241572 | 18 | 77580712 | A | G | 0.037 | 0.006 | 9.49336E-10 |  | A | G | 0.038 | 0.029 | 0.197 | 6.21038E-05 | 37.426 |
| TB-BMD-4 | GERD | rs7527682 | 1 | 189172684 | G | A | -0.027 | 0.005 | 3.12831E-08 |  | G | A | 0.010 | 0.023 | 0.674 | 5.08206E-05 | 30.626 |
| TB-BMD-4 | GERD | rs7541875 | 1 | 190957589 | G | A | 0.027 | 0.005 | 1.61213E-08 |  | G | A | -0.018 | 0.023 | 0.431 | 5.29562E-05 | 31.913 |
| TB-BMD-4 | GERD | rs7600261 | 2 | 212622818 | T | C | 0.034 | 0.005 | 9.47327E-11 |  | T | C | 0.033 | 0.026 | 0.203 | 6.95717E-05 | 41.927 |
| TB-BMD-4 | GERD | rs7612999 | 3 | 35678337 | A | G | 0.031 | 0.006 | 4.89824E-08 |  | A | G | 0.032 | 0.026 | 0.212 | 4.93779E-05 | 29.757 |
| TB-BMD-4 | GERD | rs761777 | 10 | 134938075 | G | A | 0.035 | 0.006 | 4.71455E-10 |  | G | A | 0.025 | 0.034 | 0.466 | 6.43697E-05 | 38.792 |
| TB-BMD-4 | GERD | rs7675588 | 4 | 80734978 | A | C | -0.034 | 0.006 | 1.80264E-08 |  | A | C | -0.027 | 0.027 | 0.310 | 5.2596E-05 | 31.696 |
| TB-BMD-4 | GERD | rs7685686 | 4 | 3207142 | G | A | -0.028 | 0.005 | 1.14427E-08 |  | G | A | -0.012 | 0.023 | 0.613 | 5.40613E-05 | 32.579 |
| TB-BMD-4 | GERD | rs773109 | 12 | 56374695 | A | G | -0.038 | 0.005 | 8.71365E-14 |  | A | G | 0.020 | 0.024 | 0.421 | 9.23206E-05 | 55.638 |
| TB-BMD-4 | GERD | rs7942368 | 11 | 76465362 | T | C | -0.034 | 0.006 | 9.54135E-09 |  | T | C | -0.027 | 0.026 | 0.307 | 5.46475E-05 | 32.932 |
| TB-BMD-4 | GERD | rs903678 | 1 | 201809918 | A | G | 0.028 | 0.005 | 4.89091E-08 |  | A | G | 0.020 | 0.024 | 0.401 | 4.93824E-05 | 29.759 |
| TB-BMD-4 | GERD | rs903959 | 8 | 142630782 | A | T | 0.029 | 0.005 | 2.9893E-09 |  | A | T | 0.027 | 0.023 | 0.241 | 5.83945E-05 | 35.191 |
| TB-BMD-4 | GERD | rs9372625 | 6 | 98344031 | A | G | -0.038 | 0.005 | 2.61818E-14 |  | A | G | 0.030 | 0.025 | 0.225 | 9.62435E-05 | 58.002 |
| TB-BMD-4 | GERD | rs9373363 | 6 | 143150043 | G | A | -0.033 | 0.006 | 4.13457E-09 |  | G | A | 0.035 | 0.025 | 0.167 | 5.73464E-05 | 34.559 |
| TB-BMD-4 | GERD | rs9396740 | 6 | 17023108 | A | G | -0.031 | 0.006 | 1.46562E-08 |  | A | G | -0.028 | 0.026 | 0.277 | 5.32631E-05 | 32.098 |
| TB-BMD-4 | GERD | rs942065 | 14 | 94032065 | A | G | 0.031 | 0.005 | 8.44559E-10 |  | A | G | 0.004 | 0.024 | 0.866 | 6.24824E-05 | 37.654 |
| TB-BMD-4 | GERD | rs9517313 | 13 | 99105892 | C | G | 0.033 | 0.005 | 2.04786E-11 |  | C | G | -0.011 | 0.024 | 0.644 | 7.45453E-05 | 44.925 |
| TB-BMD-4 | GERD | rs9529055 | 13 | 66957533 | A | G | 0.027 | 0.005 | 3.10549E-08 |  | A | G | -0.019 | 0.023 | 0.417 | 5.0844E-05 | 30.640 |
| TB-BMD-4 | GERD | rs9542729 | 13 | 31833578 | G | C | -0.036 | 0.006 | 1.41205E-09 |  | G | C | -0.040 | 0.030 | 0.184 | 6.08192E-05 | 36.652 |
| TB-BMD-4 | GERD | rs9615905 | 22 | 48875699 | T | C | 0.028 | 0.005 | 1.21233E-08 |  | T | C | 0.003 | 0.023 | 0.886 | 5.38749E-05 | 32.467 |
| TB-BMD-4 | GERD | rs9636202 | 19 | 18449238 | A | G | -0.035 | 0.005 | 1.51022E-10 |  | A | G | 0.022 | 0.026 | 0.396 | 6.80594E-05 | 41.016 |
| TB-BMD-4 | GERD | rs9940128 | 16 | 53800754 | A | G | 0.033 | 0.005 | 8.05749E-12 |  | A | G | -0.031 | 0.023 | 0.180 | 7.75772E-05 | 46.752 |
| TB-BMD-5 | GERD | rs10010963 | 4 | 159839313 | T | C | -0.027 | 0.005 | 4.91779E-08 |  | T | C | -0.009 | 0.013 | 0.485 | 4.93648E-05 | 29.749 |
| TB-BMD-5 | GERD | rs1011407 | 2 | 60665768 | G | A | -0.042 | 0.007 | 1.09343E-08 |  | G | A | 0.040 | 0.020 | 0.050 | 5.42079E-05 | 32.668 |
| TB-BMD-5 | GERD | rs10133111 | 14 | 103377321 | A | G | 0.042 | 0.007 | 1.35338E-10 |  | A | G | -0.004 | 0.017 | 0.821 | 6.84149E-05 | 41.230 |
| TB-BMD-5 | GERD | rs1021363 | 10 | 106610839 | G | A | -0.031 | 0.005 | 5.09976E-10 |  | G | A | 0.001 | 0.014 | 0.929 | 6.41154E-05 | 38.639 |
| TB-BMD-5 | GERD | rs10837002 | 11 | 38565727 | G | C | 0.028 | 0.005 | 4.02893E-08 |  | G | C | 0.003 | 0.014 | 0.850 | 5.00063E-05 | 30.135 |
| TB-BMD-5 | GERD | rs11762636 | 7 | 2061111 | A | C | -0.051 | 0.006 | 1.88235E-16 |  | A | C | 0.026 | 0.016 | 0.120 | 0.00011237 | 67.722 |
| TB-BMD-5 | GERD | rs11953061 | 5 | 120144025 | T | C | 0.028 | 0.005 | 3.09949E-08 |  | T | C | 0.000 | 0.014 | 0.976 | 5.08505E-05 | 30.644 |
| TB-BMD-5 | GERD | rs12204714 | 6 | 152235339 | T | C | -0.029 | 0.005 | 7.9241E-09 |  | T | C | -0.021 | 0.013 | 0.117 | 5.52467E-05 | 33.294 |
| TB-BMD-5 | GERD | rs12357321 | 10 | 21790476 | A | G | 0.032 | 0.005 | 1.33325E-09 |  | A | G | -0.004 | 0.014 | 0.779 | 6.10048E-05 | 36.764 |
| TB-BMD-5 | GERD | rs12453010 | 17 | 50316131 | T | C | 0.030 | 0.005 | 1.74606E-09 |  | T | C | -0.006 | 0.013 | 0.659 | 6.01325E-05 | 36.238 |
| TB-BMD-5 | GERD | rs12598916 | 16 | 60658751 | G | C | -0.033 | 0.005 | 6.8729E-10 |  | G | C | -0.003 | 0.014 | 0.841 | 6.31493E-05 | 38.056 |
| TB-BMD-5 | GERD | rs12967855 | 18 | 35138245 | G | A | -0.037 | 0.005 | 1.08793E-12 |  | G | A | 0.014 | 0.013 | 0.299 | 8.40922E-05 | 50.678 |
| TB-BMD-5 | GERD | rs12997558 | 2 | 41704580 | A | G | 0.028 | 0.005 | 3.03941E-08 |  | A | G | -0.006 | 0.014 | 0.661 | 5.09135E-05 | 30.682 |
| TB-BMD-5 | GERD | rs13107325 | 4 | 103188709 | T | C | 0.070 | 0.009 | 2.19938E-14 |  | T | C | 0.028 | 0.028 | 0.316 | 9.68122E-05 | 58.345 |
| TB-BMD-5 | GERD | rs1334297 | 13 | 58335375 | A | G | -0.039 | 0.005 | 1.1413E-12 |  | A | G | -0.002 | 0.015 | 0.878 | 8.39364E-05 | 50.585 |
| TB-BMD-5 | GERD | rs13409451 | 2 | 144257639 | G | A | -0.028 | 0.005 | 1.92943E-08 |  | G | A | 0.015 | 0.013 | 0.277 | 5.23772E-05 | 31.564 |
| TB-BMD-5 | GERD | rs1431196 | 18 | 50832102 | G | A | 0.032 | 0.005 | 2.65461E-11 |  | G | A | 0.028 | 0.013 | 0.032 | 7.37023E-05 | 44.416 |
| TB-BMD-5 | GERD | rs1479405 | 12 | 15387519 | T | C | 0.031 | 0.005 | 9.85213E-10 |  | T | C | 0.033 | 0.014 | 0.015 | 6.19839E-05 | 37.354 |
| TB-BMD-5 | GERD | rs1510719 | 4 | 140938116 | C | T | -0.039 | 0.005 | 3.83619E-15 |  | C | T | -0.006 | 0.014 | 0.682 | 0.000102515 | 61.782 |
| TB-BMD-5 | GERD | rs1592757 | 5 | 103889998 | C | G | 0.031 | 0.005 | 5.9975E-10 |  | C | G | 0.006 | 0.014 | 0.673 | 6.35905E-05 | 38.322 |
| TB-BMD-5 | GERD | rs1596747 | 2 | 193802478 | G | A | 0.031 | 0.005 | 1.00344E-10 |  | G | A | 0.025 | 0.013 | 0.056 | 6.93855E-05 | 41.815 |
| TB-BMD-5 | GERD | rs1716171 | 12 | 123716376 | T | C | 0.038 | 0.006 | 7.82528E-11 |  | T | C | 0.028 | 0.016 | 0.069 | 7.01925E-05 | 42.301 |
| TB-BMD-5 | GERD | rs17379561 | 1 | 98340139 | T | A | 0.053 | 0.007 | 1.07622E-14 |  | T | A | 0.002 | 0.019 | 0.898 | 9.91454E-05 | 59.751 |
| TB-BMD-5 | GERD | rs1883842 | 20 | 41223062 | G | T | 0.031 | 0.005 | 9.26766E-09 |  | G | T | 0.014 | 0.015 | 0.336 | 5.47412E-05 | 32.989 |
| TB-BMD-5 | GERD | rs1937450 | 1 | 66478840 | G | T | 0.032 | 0.005 | 7.06806E-11 |  | G | T | -0.018 | 0.013 | 0.181 | 7.05223E-05 | 42.500 |
| TB-BMD-5 | GERD | rs2016933 | 3 | 65653157 | G | C | -0.031 | 0.005 | 1.04275E-08 |  | G | C | 0.004 | 0.014 | 0.778 | 5.43609E-05 | 32.760 |
| TB-BMD-5 | GERD | rs2023878 | 19 | 18834124 | T | C | -0.036 | 0.006 | 3.03501E-09 |  | T | C | 0.003 | 0.016 | 0.861 | 5.83456E-05 | 35.161 |
| TB-BMD-5 | GERD | rs2043539 | 7 | 12253880 | A | G | 0.027 | 0.005 | 2.24042E-08 |  | A | G | 0.037 | 0.013 | 0.004 | 5.18955E-05 | 31.274 |
| TB-BMD-5 | GERD | rs2106353 | 7 | 126506598 | T | G | 0.037 | 0.006 | 1.37177E-10 |  | T | G | -0.010 | 0.016 | 0.543 | 6.83712E-05 | 41.203 |
| TB-BMD-5 | GERD | rs215614 | 7 | 32347335 | A | G | -0.033 | 0.005 | 4.08413E-11 |  | A | G | -0.008 | 0.014 | 0.583 | 7.23027E-05 | 43.573 |
| TB-BMD-5 | GERD | rs2164300 | 4 | 67813017 | T | C | -0.026 | 0.005 | 4.13352E-08 |  | T | C | 0.007 | 0.013 | 0.587 | 4.99239E-05 | 30.086 |
| TB-BMD-5 | GERD | rs2240326 | 3 | 50128386 | A | G | -0.047 | 0.005 | 1.13214E-22 |  | A | G | -0.012 | 0.013 | 0.354 | 0.000159331 | 96.029 |
| TB-BMD-5 | GERD | rs2396133 | 7 | 109197067 | G | A | 0.029 | 0.005 | 1.10889E-09 |  | G | A | 0.003 | 0.013 | 0.814 | 6.16008E-05 | 37.123 |
| TB-BMD-5 | GERD | rs2396766 | 7 | 114318071 | A | G | 0.032 | 0.005 | 2.33507E-11 |  | A | G | -0.001 | 0.013 | 0.968 | 7.41186E-05 | 44.667 |
| TB-BMD-5 | GERD | rs2734839 | 11 | 113286490 | T | C | -0.028 | 0.005 | 8.78537E-09 |  | T | C | 0.001 | 0.013 | 0.957 | 5.49136E-05 | 33.093 |
| TB-BMD-5 | GERD | rs2744961 | 6 | 34655000 | T | C | 0.029 | 0.005 | 5.81126E-09 |  | T | C | -0.019 | 0.013 | 0.154 | 5.62475E-05 | 33.897 |
| TB-BMD-5 | GERD | rs2782641 | 1 | 44013355 | A | G | 0.027 | 0.005 | 4.32643E-08 |  | A | G | 0.006 | 0.014 | 0.656 | 4.9777E-05 | 29.997 |
| TB-BMD-5 | GERD | rs2815749 | 1 | 72814783 | G | A | 0.039 | 0.006 | 1.07359E-10 |  | G | A | -0.001 | 0.017 | 0.970 | 6.9166E-05 | 41.682 |
| TB-BMD-5 | GERD | rs2834005 | 21 | 34291708 | C | T | 0.030 | 0.005 | 9.42041E-09 |  | C | T | 0.008 | 0.014 | 0.582 | 5.46885E-05 | 32.957 |
| TB-BMD-5 | GERD | rs2838771 | 21 | 46501576 | C | G | -0.028 | 0.005 | 2.90964E-08 |  | C | G | 0.021 | 0.014 | 0.128 | 5.10538E-05 | 30.767 |
| TB-BMD-5 | GERD | rs324769 | 12 | 83969240 | T | C | -0.027 | 0.005 | 3.04775E-08 |  | T | C | 0.001 | 0.013 | 0.926 | 5.09044E-05 | 30.677 |
| TB-BMD-5 | GERD | rs329122 | 5 | 133864599 | A | G | -0.029 | 0.005 | 3.05485E-09 |  | A | G | -0.009 | 0.013 | 0.514 | 5.83244E-05 | 35.148 |
| TB-BMD-5 | GERD | rs3766823 | 1 | 32197257 | A | G | 0.039 | 0.006 | 7.09316E-10 |  | A | G | 0.009 | 0.017 | 0.611 | 6.30472E-05 | 37.995 |
| TB-BMD-5 | GERD | rs3793577 | 9 | 23737627 | G | A | 0.027 | 0.005 | 2.49436E-08 |  | G | A | 0.013 | 0.013 | 0.332 | 5.15497E-05 | 31.066 |
| TB-BMD-5 | GERD | rs3828917 | 6 | 31465917 | T | G | 0.067 | 0.012 | 2.26945E-08 |  | T | G | 0.024 | 0.034 | 0.482 | 5.18541E-05 | 31.249 |
| TB-BMD-5 | GERD | rs3863241 | 8 | 73890335 | T | C | 0.032 | 0.005 | 1.48799E-11 |  | T | C | 0.015 | 0.013 | 0.249 | 7.55829E-05 | 45.550 |
| TB-BMD-5 | GERD | rs4300861 | 2 | 22549441 | T | C | 0.031 | 0.005 | 5.431E-10 |  | T | C | 0.001 | 0.013 | 0.961 | 6.39117E-05 | 38.516 |
| TB-BMD-5 | GERD | rs4382592 | 9 | 134870755 | G | T | -0.030 | 0.005 | 8.19917E-09 |  | G | T | 0.023 | 0.014 | 0.088 | 5.51365E-05 | 33.227 |
| TB-BMD-5 | GERD | rs4713692 | 6 | 33807638 | T | C | -0.028 | 0.005 | 3.06507E-08 |  | T | C | -0.006 | 0.014 | 0.679 | 5.08866E-05 | 30.666 |
| TB-BMD-5 | GERD | rs569356 | 1 | 29136686 | G | A | -0.038 | 0.007 | 4.07005E-08 |  | G | A | -0.031 | 0.019 | 0.107 | 4.99736E-05 | 30.116 |
| TB-BMD-5 | GERD | rs6711584 | 2 | 104421692 | A | G | 0.032 | 0.005 | 2.65583E-11 |  | A | G | -0.003 | 0.013 | 0.822 | 7.37009E-05 | 44.416 |
| TB-BMD-5 | GERD | rs6722661 | 2 | 100806588 | A | G | -0.032 | 0.005 | 1.14623E-10 |  | A | G | -0.019 | 0.014 | 0.165 | 6.89539E-05 | 41.555 |
| TB-BMD-5 | GERD | rs6780459 | 3 | 104624105 | T | A | 0.031 | 0.006 | 3.13993E-08 |  | T | A | -0.027 | 0.015 | 0.063 | 5.08086E-05 | 30.619 |
| TB-BMD-5 | GERD | rs7032155 | 9 | 122672771 | A | C | 0.028 | 0.005 | 1.62686E-08 |  | A | C | 0.005 | 0.013 | 0.721 | 5.29267E-05 | 31.895 |
| TB-BMD-5 | GERD | rs7206608 | 16 | 82872628 | G | C | 0.029 | 0.005 | 1.45761E-08 |  | G | C | -0.010 | 0.014 | 0.494 | 5.32811E-05 | 32.109 |
| TB-BMD-5 | GERD | rs7241572 | 18 | 77580712 | A | G | 0.037 | 0.006 | 9.49336E-10 |  | A | G | -0.012 | 0.017 | 0.478 | 6.21038E-05 | 37.426 |
| TB-BMD-5 | GERD | rs7527682 | 1 | 189172684 | G | A | -0.027 | 0.005 | 3.12831E-08 |  | G | A | 0.002 | 0.013 | 0.885 | 5.08206E-05 | 30.626 |
| TB-BMD-5 | GERD | rs7541875 | 1 | 190957589 | G | A | 0.027 | 0.005 | 1.61213E-08 |  | G | A | 0.004 | 0.013 | 0.737 | 5.29562E-05 | 31.913 |
| TB-BMD-5 | GERD | rs7600261 | 2 | 212622818 | T | C | 0.034 | 0.005 | 9.47327E-11 |  | T | C | -0.011 | 0.014 | 0.436 | 6.95717E-05 | 41.927 |
| TB-BMD-5 | GERD | rs7612999 | 3 | 35678337 | A | G | 0.031 | 0.006 | 4.89824E-08 |  | A | G | 0.018 | 0.015 | 0.234 | 4.93779E-05 | 29.757 |
| TB-BMD-5 | GERD | rs761777 | 10 | 134938075 | G | A | 0.035 | 0.006 | 4.71455E-10 |  | G | A | -0.022 | 0.017 | 0.187 | 6.43697E-05 | 38.792 |
| TB-BMD-5 | GERD | rs7675588 | 4 | 80734978 | A | C | -0.034 | 0.006 | 1.80264E-08 |  | A | C | -0.013 | 0.015 | 0.416 | 5.2596E-05 | 31.696 |
| TB-BMD-5 | GERD | rs7685686 | 4 | 3207142 | G | A | -0.028 | 0.005 | 1.14427E-08 |  | G | A | 0.010 | 0.013 | 0.465 | 5.40613E-05 | 32.579 |
| TB-BMD-5 | GERD | rs773109 | 12 | 56374695 | A | G | -0.038 | 0.005 | 8.71365E-14 |  | A | G | 0.015 | 0.014 | 0.295 | 9.23206E-05 | 55.638 |
| TB-BMD-5 | GERD | rs7942368 | 11 | 76465362 | T | C | -0.034 | 0.006 | 9.54135E-09 |  | T | C | -0.002 | 0.015 | 0.914 | 5.46475E-05 | 32.932 |
| TB-BMD-5 | GERD | rs903678 | 1 | 201809918 | A | G | 0.028 | 0.005 | 4.89091E-08 |  | A | G | -0.004 | 0.014 | 0.760 | 4.93824E-05 | 29.759 |
| TB-BMD-5 | GERD | rs903959 | 8 | 142630782 | A | T | 0.029 | 0.005 | 2.9893E-09 |  | A | T | 0.011 | 0.013 | 0.396 | 5.83945E-05 | 35.191 |
| TB-BMD-5 | GERD | rs9372625 | 6 | 98344031 | A | G | -0.038 | 0.005 | 2.61818E-14 |  | A | G | 0.015 | 0.014 | 0.274 | 9.62435E-05 | 58.002 |
| TB-BMD-5 | GERD | rs9373363 | 6 | 143150043 | G | A | -0.033 | 0.006 | 4.13457E-09 |  | G | A | -0.024 | 0.015 | 0.104 | 5.73464E-05 | 34.559 |
| TB-BMD-5 | GERD | rs9396740 | 6 | 17023108 | A | G | -0.031 | 0.006 | 1.46562E-08 |  | A | G | 0.018 | 0.015 | 0.222 | 5.32631E-05 | 32.098 |
| TB-BMD-5 | GERD | rs942065 | 14 | 94032065 | A | G | 0.031 | 0.005 | 8.44559E-10 |  | A | G | 0.009 | 0.013 | 0.506 | 6.24824E-05 | 37.654 |
| TB-BMD-5 | GERD | rs9517313 | 13 | 99105892 | C | G | 0.033 | 0.005 | 2.04786E-11 |  | C | G | -0.017 | 0.013 | 0.200 | 7.45453E-05 | 44.925 |
| TB-BMD-5 | GERD | rs9529055 | 13 | 66957533 | A | G | 0.027 | 0.005 | 3.10549E-08 |  | A | G | 0.022 | 0.013 | 0.091 | 5.0844E-05 | 30.640 |
| TB-BMD-5 | GERD | rs9542729 | 13 | 31833578 | G | C | -0.036 | 0.006 | 1.41205E-09 |  | G | C | -0.016 | 0.016 | 0.323 | 6.08192E-05 | 36.652 |
| TB-BMD-5 | GERD | rs9615905 | 22 | 48875699 | T | C | 0.028 | 0.005 | 1.21233E-08 |  | T | C | -0.009 | 0.013 | 0.491 | 5.38749E-05 | 32.467 |
| TB-BMD-5 | GERD | rs9636202 | 19 | 18449238 | A | G | -0.035 | 0.005 | 1.51022E-10 |  | A | G | 0.002 | 0.015 | 0.879 | 6.80594E-05 | 41.016 |
| TB-BMD-5 | GERD | rs9940128 | 16 | 53800754 | A | G | 0.033 | 0.005 | 8.05749E-12 |  | A | G | 0.003 | 0.013 | 0.816 | 7.75772E-05 | 46.752 |
| FN-BMD | GERD | rs10010963 | 4 | 159839313 | T | C | -0.027 | 0.005 | 4.91779E-08 |  | T | C | -0.010 | 0.008 | 0.187 | 4.93648E-05 | 29.749 |
| FN-BMD | GERD | rs1011407 | 2 | 60665768 | G | A | -0.042 | 0.007 | 1.09343E-08 |  | G | A | -0.016 | 0.011 | 0.182 | 5.42079E-05 | 32.668 |
| FN-BMD | GERD | rs10133111 | 14 | 103377321 | A | G | 0.042 | 0.007 | 1.35338E-10 |  | A | G | -0.002 | 0.010 | 0.825 | 6.84149E-05 | 41.230 |
| FN-BMD | GERD | rs1021363 | 10 | 106610839 | G | A | -0.031 | 0.005 | 5.09976E-10 |  | G | A | 0.007 | 0.008 | 0.400 | 6.41154E-05 | 38.639 |
| FN-BMD | GERD | rs10837002 | 11 | 38565727 | G | C | 0.028 | 0.005 | 4.02893E-08 |  | G | C | -0.005 | 0.008 | 0.534 | 5.00063E-05 | 30.135 |
| FN-BMD | GERD | rs11762636 | 7 | 2061111 | A | C | -0.051 | 0.006 | 1.88235E-16 |  | A | C | 0.002 | 0.009 | 0.820 | 0.00011237 | 67.722 |
| FN-BMD | GERD | rs11953061 | 5 | 120144025 | T | C | 0.028 | 0.005 | 3.09949E-08 |  | T | C | -0.006 | 0.008 | 0.450 | 5.08505E-05 | 30.644 |
| FN-BMD | GERD | rs12204714 | 6 | 152235339 | T | C | -0.029 | 0.005 | 7.9241E-09 |  | T | C | 0.006 | 0.008 | 0.423 | 5.52467E-05 | 33.294 |
| FN-BMD | GERD | rs12357321 | 10 | 21790476 | A | G | 0.032 | 0.005 | 1.33325E-09 |  | A | G | -0.010 | 0.008 | 0.219 | 6.10048E-05 | 36.764 |
| FN-BMD | GERD | rs12453010 | 17 | 50316131 | T | C | 0.030 | 0.005 | 1.74606E-09 |  | T | C | -0.003 | 0.008 | 0.671 | 6.01325E-05 | 36.238 |
| FN-BMD | GERD | rs12598916 | 16 | 60658751 | G | C | -0.033 | 0.005 | 6.8729E-10 |  | G | C | -0.003 | 0.008 | 0.746 | 6.31493E-05 | 38.056 |
| FN-BMD | GERD | rs12967855 | 18 | 35138245 | G | A | -0.037 | 0.005 | 1.08793E-12 |  | G | A | -0.001 | 0.008 | 0.879 | 8.40922E-05 | 50.678 |
| FN-BMD | GERD | rs12997558 | 2 | 41704580 | A | G | 0.028 | 0.005 | 3.03941E-08 |  | A | G | -0.008 | 0.008 | 0.330 | 5.09135E-05 | 30.682 |
| FN-BMD | GERD | rs13107325 | 4 | 103198082 | T | C | 0.070 | 0.009 | 2.19938E-14 |  | T | C | 0.018 | 0.014 | 0.208 | 9.68122E-05 | 58.345 |
| FN-BMD | GERD | rs1334297 | 13 | 58335375 | A | G | -0.039 | 0.005 | 1.1413E-12 |  | A | G | 0.005 | 0.009 | 0.588 | 8.39364E-05 | 50.585 |
| FN-BMD | GERD | rs13409451 | 2 | 144257639 | G | A | -0.028 | 0.005 | 1.92943E-08 |  | G | A | -0.007 | 0.008 | 0.397 | 5.23772E-05 | 31.564 |
| FN-BMD | GERD | rs1431196 | 18 | 50845830 | G | A | 0.032 | 0.005 | 2.65461E-11 |  | G | A | 0.006 | 0.008 | 0.457 | 7.37023E-05 | 44.416 |
| FN-BMD | GERD | rs1479405 | 12 | 15387519 | T | C | 0.031 | 0.005 | 9.85213E-10 |  | T | C | 0.009 | 0.008 | 0.248 | 6.19839E-05 | 37.354 |
| FN-BMD | GERD | rs1510719 | 4 | 140938116 | C | T | -0.039 | 0.005 | 3.83619E-15 |  | C | T | 0.021 | 0.008 | 0.008 | 0.000102515 | 61.782 |
| FN-BMD | GERD | rs1592757 | 5 | 103889998 | C | G | 0.031 | 0.005 | 5.9975E-10 |  | C | G | -0.010 | 0.008 | 0.218 | 6.35905E-05 | 38.322 |
| FN-BMD | GERD | rs1596747 | 2 | 193802478 | G | A | 0.031 | 0.005 | 1.00344E-10 |  | G | A | 0.003 | 0.008 | 0.756 | 6.93855E-05 | 41.815 |
| FN-BMD | GERD | rs1716171 | 12 | 123716376 | T | C | 0.038 | 0.006 | 7.82528E-11 |  | T | C | -0.005 | 0.010 | 0.613 | 7.01925E-05 | 42.301 |
| FN-BMD | GERD | rs17379561 | 1 | 98340139 | T | A | 0.053 | 0.007 | 1.07622E-14 |  | T | A | -0.009 | 0.011 | 0.435 | 9.91454E-05 | 59.751 |
| FN-BMD | GERD | rs1883842 | 20 | 41223062 | G | T | 0.031 | 0.005 | 9.26766E-09 |  | G | T | -0.009 | 0.009 | 0.305 | 5.47412E-05 | 32.989 |
| FN-BMD | GERD | rs1937450 | 1 | 66478840 | G | T | 0.032 | 0.005 | 7.06806E-11 |  | G | T | 0.004 | 0.008 | 0.640 | 7.05223E-05 | 42.500 |
| FN-BMD | GERD | rs2016933 | 3 | 65653157 | G | C | -0.031 | 0.005 | 1.04275E-08 |  | G | C | -0.004 | 0.008 | 0.615 | 5.43609E-05 | 32.760 |
| FN-BMD | GERD | rs2023878 | 19 | 18834124 | T | C | -0.036 | 0.006 | 3.03501E-09 |  | T | C | -0.014 | 0.010 | 0.154 | 5.83456E-05 | 35.161 |
| FN-BMD | GERD | rs2043539 | 7 | 12253880 | A | G | 0.027 | 0.005 | 2.24042E-08 |  | A | G | 0.007 | 0.008 | 0.343 | 5.18955E-05 | 31.274 |
| FN-BMD | GERD | rs2106353 | 7 | 126506598 | T | G | 0.037 | 0.006 | 1.37177E-10 |  | T | G | 0.004 | 0.009 | 0.637 | 6.83712E-05 | 41.203 |
| FN-BMD | GERD | rs215614 | 7 | 32347335 | A | G | -0.033 | 0.005 | 4.08413E-11 |  | A | G | 0.013 | 0.008 | 0.102 | 7.23027E-05 | 43.573 |
| FN-BMD | GERD | rs2164300 | 4 | 67817416 | T | C | -0.026 | 0.005 | 4.13352E-08 |  | T | C | 0.003 | 0.008 | 0.711 | 4.99239E-05 | 30.086 |
| FN-BMD | GERD | rs2240326 | 3 | 50128386 | A | G | -0.047 | 0.005 | 1.13214E-22 |  | A | G | 0.002 | 0.008 | 0.826 | 0.000159331 | 96.029 |
| FN-BMD | GERD | rs2396133 | 7 | 109197067 | G | A | 0.029 | 0.005 | 1.10889E-09 |  | G | A | 0.008 | 0.008 | 0.321 | 6.16008E-05 | 37.123 |
| FN-BMD | GERD | rs2396766 | 7 | 114318071 | A | G | 0.032 | 0.005 | 2.33507E-11 |  | A | G | 0.001 | 0.008 | 0.886 | 7.41186E-05 | 44.667 |
| FN-BMD | GERD | rs2734839 | 11 | 113286490 | T | C | -0.028 | 0.005 | 8.78537E-09 |  | T | C | -0.011 | 0.008 | 0.179 | 5.49136E-05 | 33.093 |
| FN-BMD | GERD | rs2744961 | 6 | 34655000 | T | C | 0.029 | 0.005 | 5.81126E-09 |  | T | C | 0.005 | 0.008 | 0.564 | 5.62475E-05 | 33.897 |
| FN-BMD | GERD | rs2782641 | 1 | 44013355 | A | G | 0.027 | 0.005 | 4.32643E-08 |  | A | G | -0.008 | 0.008 | 0.339 | 4.9777E-05 | 29.997 |
| FN-BMD | GERD | rs2815749 | 1 | 72814783 | G | A | 0.039 | 0.006 | 1.07359E-10 |  | G | A | 0.004 | 0.010 | 0.657 | 6.9166E-05 | 41.682 |
| FN-BMD | GERD | rs2834005 | 21 | 34291708 | C | T | 0.030 | 0.005 | 9.42041E-09 |  | C | T | -0.002 | 0.008 | 0.797 | 5.46885E-05 | 32.957 |
| FN-BMD | GERD | rs2838771 | 21 | 46501576 | C | G | -0.028 | 0.005 | 2.90964E-08 |  | C | G | 0.000 | 0.008 | 0.971 | 5.10538E-05 | 30.767 |
| FN-BMD | GERD | rs324769 | 12 | 83969240 | T | C | -0.027 | 0.005 | 3.04775E-08 |  | T | C | 0.008 | 0.008 | 0.332 | 5.09044E-05 | 30.677 |
| FN-BMD | GERD | rs329122 | 5 | 133865452 | A | G | -0.029 | 0.005 | 3.05485E-09 |  | A | G | -0.004 | 0.008 | 0.604 | 5.83244E-05 | 35.148 |
| FN-BMD | GERD | rs3766823 | 1 | 32197257 | A | G | 0.039 | 0.006 | 7.09316E-10 |  | A | G | 0.006 | 0.010 | 0.548 | 6.30472E-05 | 37.995 |
| FN-BMD | GERD | rs3793577 | 9 | 23737627 | G | A | 0.027 | 0.005 | 2.49436E-08 |  | G | A | -0.003 | 0.008 | 0.692 | 5.15497E-05 | 31.066 |
| FN-BMD | GERD | rs3828917 | 6 | 31468404 | T | G | 0.067 | 0.012 | 2.26945E-08 |  | T | G | 0.048 | 0.023 | 0.036 | 5.18541E-05 | 31.249 |
| FN-BMD | GERD | rs3863241 | 8 | 73890335 | T | C | 0.032 | 0.005 | 1.48799E-11 |  | T | C | -0.003 | 0.007 | 0.649 | 7.55829E-05 | 45.550 |
| FN-BMD | GERD | rs4300861 | 2 | 22549441 | T | C | 0.031 | 0.005 | 5.431E-10 |  | T | C | 0.008 | 0.008 | 0.343 | 6.39117E-05 | 38.516 |
| FN-BMD | GERD | rs4382592 | 9 | 134870755 | G | T | -0.030 | 0.005 | 8.19917E-09 |  | G | T | -0.004 | 0.008 | 0.632 | 5.51365E-05 | 33.227 |
| FN-BMD | GERD | rs4713692 | 6 | 33810005 | T | C | -0.028 | 0.005 | 3.06507E-08 |  | T | C | -0.002 | 0.008 | 0.790 | 5.08866E-05 | 30.666 |
| FN-BMD | GERD | rs569356 | 1 | 29136686 | G | A | -0.038 | 0.007 | 4.07005E-08 |  | G | A | -0.006 | 0.011 | 0.621 | 4.99736E-05 | 30.116 |
| FN-BMD | GERD | rs6711584 | 2 | 104421692 | A | G | 0.032 | 0.005 | 2.65583E-11 |  | A | G | 0.011 | 0.008 | 0.175 | 7.37009E-05 | 44.416 |
| FN-BMD | GERD | rs6722661 | 2 | 100806588 | A | G | -0.032 | 0.005 | 1.14623E-10 |  | A | G | 0.006 | 0.008 | 0.454 | 6.89539E-05 | 41.555 |
| FN-BMD | GERD | rs6780459 | 3 | 104624105 | T | A | 0.031 | 0.006 | 3.13993E-08 |  | T | A | -0.008 | 0.009 | 0.354 | 5.08086E-05 | 30.619 |
| FN-BMD | GERD | rs7032155 | 9 | 122672771 | A | C | 0.028 | 0.005 | 1.62686E-08 |  | A | C | -0.007 | 0.008 | 0.353 | 5.29267E-05 | 31.895 |
| FN-BMD | GERD | rs7206608 | 16 | 82872628 | G | C | 0.029 | 0.005 | 1.45761E-08 |  | G | C | 0.010 | 0.008 | 0.212 | 5.32811E-05 | 32.109 |
| FN-BMD | GERD | rs7241572 | 18 | 77580712 | A | G | 0.037 | 0.006 | 9.49336E-10 |  | A | G | 0.016 | 0.010 | 0.105 | 6.21038E-05 | 37.426 |
| FN-BMD | GERD | rs7527682 | 1 | 189172684 | G | A | -0.027 | 0.005 | 3.12831E-08 |  | G | A | -0.028 | 0.008 | 0.000 | 5.08206E-05 | 30.626 |
| FN-BMD | GERD | rs7541875 | 1 | 190957589 | G | A | 0.027 | 0.005 | 1.61213E-08 |  | G | A | -0.002 | 0.008 | 0.813 | 5.29562E-05 | 31.913 |
| FN-BMD | GERD | rs7600261 | 2 | 212622818 | T | C | 0.034 | 0.005 | 9.47327E-11 |  | T | C | 0.008 | 0.009 | 0.347 | 6.95717E-05 | 41.927 |
| FN-BMD | GERD | rs7612999 | 3 | 35678337 | A | G | 0.031 | 0.006 | 4.89824E-08 |  | A | G | 0.001 | 0.009 | 0.879 | 4.93779E-05 | 29.757 |
| FN-BMD | GERD | rs761777 | 10 | 134938075 | G | A | 0.035 | 0.006 | 4.71455E-10 |  | G | A | -0.002 | 0.009 | 0.851 | 6.43697E-05 | 38.792 |
| FN-BMD | GERD | rs7675588 | 4 | 80734978 | A | C | -0.034 | 0.006 | 1.80264E-08 |  | A | C | -0.003 | 0.009 | 0.747 | 5.2596E-05 | 31.696 |
| FN-BMD | GERD | rs7685686 | 4 | 3207142 | G | A | -0.028 | 0.005 | 1.14427E-08 |  | G | A | 0.006 | 0.008 | 0.462 | 5.40613E-05 | 32.579 |
| FN-BMD | GERD | rs773109 | 12 | 56390636 | A | G | -0.038 | 0.005 | 8.71365E-14 |  | A | G | -0.007 | 0.009 | 0.427 | 9.23206E-05 | 55.638 |
| FN-BMD | GERD | rs7942368 | 11 | 76465362 | T | C | -0.034 | 0.006 | 9.54135E-09 |  | T | C | 0.012 | 0.009 | 0.188 | 5.46475E-05 | 32.932 |
| FN-BMD | GERD | rs903678 | 1 | 201809918 | A | G | 0.028 | 0.005 | 4.89091E-08 |  | A | G | -0.002 | 0.008 | 0.787 | 4.93824E-05 | 29.759 |
| FN-BMD | GERD | rs903959 | 8 | 142630782 | A | T | 0.029 | 0.005 | 2.9893E-09 |  | A | T | 0.002 | 0.008 | 0.846 | 5.83945E-05 | 35.191 |
| FN-BMD | GERD | rs9372625 | 6 | 98344031 | A | G | -0.038 | 0.005 | 2.61818E-14 |  | A | G | 0.005 | 0.008 | 0.517 | 9.62435E-05 | 58.002 |
| FN-BMD | GERD | rs9373363 | 6 | 143150043 | G | A | -0.033 | 0.006 | 4.13457E-09 |  | G | A | -0.006 | 0.009 | 0.546 | 5.73464E-05 | 34.559 |
| FN-BMD | GERD | rs9396740 | 6 | 17023108 | A | G | -0.031 | 0.006 | 1.46562E-08 |  | A | G | 0.011 | 0.009 | 0.216 | 5.32631E-05 | 32.098 |
| FN-BMD | GERD | rs942065 | 14 | 94032065 | A | G | 0.031 | 0.005 | 8.44559E-10 |  | A | G | 0.003 | 0.008 | 0.678 | 6.24824E-05 | 37.654 |
| FN-BMD | GERD | rs9517313 | 13 | 99105892 | C | G | 0.033 | 0.005 | 2.04786E-11 |  | C | G | -0.013 | 0.008 | 0.123 | 7.45453E-05 | 44.925 |
| FN-BMD | GERD | rs9529055 | 13 | 66957533 | A | G | 0.027 | 0.005 | 3.10549E-08 |  | A | G | 0.001 | 0.007 | 0.928 | 5.0844E-05 | 30.640 |
| FN-BMD | GERD | rs9542729 | 13 | 31833578 | G | C | -0.036 | 0.006 | 1.41205E-09 |  | G | C | 0.008 | 0.009 | 0.417 | 6.08192E-05 | 36.652 |
| FN-BMD | GERD | rs9615905 | 22 | 48875699 | T | C | 0.028 | 0.005 | 1.21233E-08 |  | T | C | -0.010 | 0.009 | 0.280 | 5.38749E-05 | 32.467 |
| FN-BMD | GERD | rs9636202 | 19 | 18449238 | A | G | -0.035 | 0.005 | 1.51022E-10 |  | A | G | 0.002 | 0.008 | 0.798 | 6.80594E-05 | 41.016 |
| FN-BMD | GERD | rs9940128 | 16 | 53800754 | A | G | 0.033 | 0.005 | 8.05749E-12 |  | A | G | -0.012 | 0.008 | 0.127 | 7.75772E-05 | 46.752 |
| LS-BMD | GERD | rs10010963 | 4 | 159839313 | T | C | -0.027 | 0.005 | 4.91779E-08 |  | T | C | -0.015 | 0.009 | 0.117 | 4.93648E-05 | 29.749 |
| LS-BMD | GERD | rs1011407 | 2 | 60665768 | G | A | -0.042 | 0.007 | 1.09343E-08 |  | G | A | 0.017 | 0.013 | 0.223 | 5.42079E-05 | 32.668 |
| LS-BMD | GERD | rs10133111 | 14 | 103377321 | A | G | 0.042 | 0.007 | 1.35338E-10 |  | A | G | -0.027 | 0.012 | 0.025 | 6.84149E-05 | 41.230 |
| LS-BMD | GERD | rs1021363 | 10 | 106610839 | G | A | -0.031 | 0.005 | 5.09976E-10 |  | G | A | 0.002 | 0.009 | 0.832 | 6.41154E-05 | 38.639 |
| LS-BMD | GERD | rs10837002 | 11 | 38565727 | G | C | 0.028 | 0.005 | 4.02893E-08 |  | G | C | 0.015 | 0.009 | 0.114 | 5.00063E-05 | 30.135 |
| LS-BMD | GERD | rs11762636 | 7 | 2061111 | A | C | -0.051 | 0.006 | 1.88235E-16 |  | A | C | 0.000 | 0.011 | 0.991 | 0.00011237 | 67.722 |
| LS-BMD | GERD | rs11953061 | 5 | 120144025 | T | C | 0.028 | 0.005 | 3.09949E-08 |  | T | C | -0.011 | 0.009 | 0.239 | 5.08505E-05 | 30.644 |
| LS-BMD | GERD | rs12204714 | 6 | 152235339 | T | C | -0.029 | 0.005 | 7.9241E-09 |  | T | C | -0.005 | 0.009 | 0.626 | 5.52467E-05 | 33.294 |
| LS-BMD | GERD | rs12357321 | 10 | 21790476 | A | G | 0.032 | 0.005 | 1.33325E-09 |  | A | G | 0.004 | 0.010 | 0.654 | 6.10048E-05 | 36.764 |
| LS-BMD | GERD | rs12453010 | 17 | 50316131 | T | C | 0.030 | 0.005 | 1.74606E-09 |  | T | C | -0.011 | 0.009 | 0.244 | 6.01325E-05 | 36.238 |
| LS-BMD | GERD | rs12598916 | 16 | 60658751 | G | C | -0.033 | 0.005 | 6.8729E-10 |  | G | C | 0.017 | 0.010 | 0.095 | 6.31493E-05 | 38.056 |
| LS-BMD | GERD | rs12967855 | 18 | 35138245 | G | A | -0.037 | 0.005 | 1.08793E-12 |  | G | A | 0.017 | 0.009 | 0.082 | 8.40922E-05 | 50.678 |
| LS-BMD | GERD | rs12997558 | 2 | 41704580 | A | G | 0.028 | 0.005 | 3.03941E-08 |  | A | G | 0.002 | 0.009 | 0.803 | 5.09135E-05 | 30.682 |
| LS-BMD | GERD | rs13107325 | 4 | 103198082 | T | C | 0.070 | 0.009 | 2.19938E-14 |  | T | C | 0.025 | 0.016 | 0.134 | 9.68122E-05 | 58.345 |
| LS-BMD | GERD | rs1334297 | 13 | 58335375 | A | G | -0.039 | 0.005 | 1.1413E-12 |  | A | G | 0.010 | 0.010 | 0.336 | 8.39364E-05 | 50.585 |
| LS-BMD | GERD | rs13409451 | 2 | 144257639 | G | A | -0.028 | 0.005 | 1.92943E-08 |  | G | A | -0.007 | 0.009 | 0.458 | 5.23772E-05 | 31.564 |
| LS-BMD | GERD | rs1431196 | 18 | 50845830 | G | A | 0.032 | 0.005 | 2.65461E-11 |  | G | A | -0.006 | 0.009 | 0.509 | 7.37023E-05 | 44.416 |
| LS-BMD | GERD | rs1479405 | 12 | 15387519 | T | C | 0.031 | 0.005 | 9.85213E-10 |  | T | C | 0.024 | 0.009 | 0.012 | 6.19839E-05 | 37.354 |
| LS-BMD | GERD | rs1510719 | 4 | 140938116 | C | T | -0.039 | 0.005 | 3.83619E-15 |  | C | T | 0.014 | 0.009 | 0.140 | 0.000102515 | 61.782 |
| LS-BMD | GERD | rs1592757 | 5 | 103889998 | C | G | 0.031 | 0.005 | 5.9975E-10 |  | C | G | -0.004 | 0.009 | 0.650 | 6.35905E-05 | 38.322 |
| LS-BMD | GERD | rs1596747 | 2 | 193802478 | G | A | 0.031 | 0.005 | 1.00344E-10 |  | G | A | 0.000 | 0.009 | 0.993 | 6.93855E-05 | 41.815 |
| LS-BMD | GERD | rs1716171 | 12 | 123716376 | T | C | 0.038 | 0.006 | 7.82528E-11 |  | T | C | 0.008 | 0.012 | 0.510 | 7.01925E-05 | 42.301 |
| LS-BMD | GERD | rs17379561 | 1 | 98340139 | T | A | 0.053 | 0.007 | 1.07622E-14 |  | T | A | -0.011 | 0.013 | 0.372 | 9.91454E-05 | 59.751 |
| LS-BMD | GERD | rs1883842 | 20 | 41223062 | G | T | 0.031 | 0.005 | 9.26766E-09 |  | G | T | 0.000 | 0.010 | 0.974 | 5.47412E-05 | 32.989 |
| LS-BMD | GERD | rs1937450 | 1 | 66478840 | G | T | 0.032 | 0.005 | 7.06806E-11 |  | G | T | 0.005 | 0.009 | 0.550 | 7.05223E-05 | 42.500 |
| LS-BMD | GERD | rs2016933 | 3 | 65653157 | G | C | -0.031 | 0.005 | 1.04275E-08 |  | G | C | -0.010 | 0.010 | 0.291 | 5.43609E-05 | 32.760 |
| LS-BMD | GERD | rs2023878 | 19 | 18834124 | T | C | -0.036 | 0.006 | 3.03501E-09 |  | T | C | -0.012 | 0.011 | 0.308 | 5.83456E-05 | 35.161 |
| LS-BMD | GERD | rs2043539 | 7 | 12253880 | A | G | 0.027 | 0.005 | 2.24042E-08 |  | A | G | 0.016 | 0.009 | 0.082 | 5.18955E-05 | 31.274 |
| LS-BMD | GERD | rs2106353 | 7 | 126506598 | T | G | 0.037 | 0.006 | 1.37177E-10 |  | T | G | 0.011 | 0.010 | 0.311 | 6.83712E-05 | 41.203 |
| LS-BMD | GERD | rs215614 | 7 | 32347335 | A | G | -0.033 | 0.005 | 4.08413E-11 |  | A | G | 0.006 | 0.009 | 0.515 | 7.23027E-05 | 43.573 |
| LS-BMD | GERD | rs2164300 | 4 | 67817416 | T | C | -0.026 | 0.005 | 4.13352E-08 |  | T | C | 0.000 | 0.009 | 0.960 | 4.99239E-05 | 30.086 |
| LS-BMD | GERD | rs2240326 | 3 | 50128386 | A | G | -0.047 | 0.005 | 1.13214E-22 |  | A | G | 0.004 | 0.009 | 0.670 | 0.000159331 | 96.029 |
| LS-BMD | GERD | rs2396133 | 7 | 109197067 | G | A | 0.029 | 0.005 | 1.10889E-09 |  | G | A | 0.007 | 0.009 | 0.416 | 6.16008E-05 | 37.123 |
| LS-BMD | GERD | rs2396766 | 7 | 114318071 | A | G | 0.032 | 0.005 | 2.33507E-11 |  | A | G | -0.003 | 0.009 | 0.764 | 7.41186E-05 | 44.667 |
| LS-BMD | GERD | rs2734839 | 11 | 113286490 | T | C | -0.028 | 0.005 | 8.78537E-09 |  | T | C | -0.010 | 0.009 | 0.262 | 5.49136E-05 | 33.093 |
| LS-BMD | GERD | rs2744961 | 6 | 34655000 | T | C | 0.029 | 0.005 | 5.81126E-09 |  | T | C | 0.000 | 0.009 | 0.987 | 5.62475E-05 | 33.897 |
| LS-BMD | GERD | rs2782641 | 1 | 44013355 | A | G | 0.027 | 0.005 | 4.32643E-08 |  | A | G | -0.001 | 0.009 | 0.909 | 4.9777E-05 | 29.997 |
| LS-BMD | GERD | rs2815749 | 1 | 72814783 | G | A | 0.039 | 0.006 | 1.07359E-10 |  | G | A | 0.007 | 0.011 | 0.534 | 6.9166E-05 | 41.682 |
| LS-BMD | GERD | rs2834005 | 21 | 34291708 | C | T | 0.030 | 0.005 | 9.42041E-09 |  | C | T | 0.011 | 0.009 | 0.272 | 5.46885E-05 | 32.957 |
| LS-BMD | GERD | rs2838771 | 21 | 46501576 | C | G | -0.028 | 0.005 | 2.90964E-08 |  | C | G | 0.006 | 0.009 | 0.527 | 5.10538E-05 | 30.767 |
| LS-BMD | GERD | rs324769 | 12 | 83969240 | T | C | -0.027 | 0.005 | 3.04775E-08 |  | T | C | 0.013 | 0.009 | 0.146 | 5.09044E-05 | 30.677 |
| LS-BMD | GERD | rs329122 | 5 | 133865452 | A | G | -0.029 | 0.005 | 3.05485E-09 |  | A | G | -0.008 | 0.009 | 0.371 | 5.83244E-05 | 35.148 |
| LS-BMD | GERD | rs3766823 | 1 | 32197257 | A | G | 0.039 | 0.006 | 7.09316E-10 |  | A | G | 0.003 | 0.012 | 0.821 | 6.30472E-05 | 37.995 |
| LS-BMD | GERD | rs3793577 | 9 | 23737627 | G | A | 0.027 | 0.005 | 2.49436E-08 |  | G | A | 0.007 | 0.009 | 0.458 | 5.15497E-05 | 31.066 |
| LS-BMD | GERD | rs3828917 | 6 | 31468404 | T | G | 0.067 | 0.012 | 2.26945E-08 |  | T | G | 0.027 | 0.028 | 0.359 | 5.18541E-05 | 31.249 |
| LS-BMD | GERD | rs3863241 | 8 | 73890335 | T | C | 0.032 | 0.005 | 1.48799E-11 |  | T | C | -0.011 | 0.009 | 0.222 | 7.55829E-05 | 45.550 |
| LS-BMD | GERD | rs4300861 | 2 | 22549441 | T | C | 0.031 | 0.005 | 5.431E-10 |  | T | C | 0.007 | 0.009 | 0.455 | 6.39117E-05 | 38.516 |
| LS-BMD | GERD | rs4382592 | 9 | 134870755 | G | T | -0.030 | 0.005 | 8.19917E-09 |  | G | T | -0.011 | 0.010 | 0.259 | 5.51365E-05 | 33.227 |
| LS-BMD | GERD | rs4713692 | 6 | 33810005 | T | C | -0.028 | 0.005 | 3.06507E-08 |  | T | C | 0.004 | 0.009 | 0.670 | 5.08866E-05 | 30.666 |
| LS-BMD | GERD | rs569356 | 1 | 29136686 | G | A | -0.038 | 0.007 | 4.07005E-08 |  | G | A | -0.007 | 0.013 | 0.579 | 4.99736E-05 | 30.116 |
| LS-BMD | GERD | rs6711584 | 2 | 104421692 | A | G | 0.032 | 0.005 | 2.65583E-11 |  | A | G | -0.001 | 0.009 | 0.874 | 7.37009E-05 | 44.416 |
| LS-BMD | GERD | rs6722661 | 2 | 100806588 | A | G | -0.032 | 0.005 | 1.14623E-10 |  | A | G | 0.007 | 0.009 | 0.481 | 6.89539E-05 | 41.555 |
| LS-BMD | GERD | rs6780459 | 3 | 104624105 | T | A | 0.031 | 0.006 | 3.13993E-08 |  | T | A | -0.008 | 0.010 | 0.449 | 5.08086E-05 | 30.619 |
| LS-BMD | GERD | rs7032155 | 9 | 122672771 | A | C | 0.028 | 0.005 | 1.62686E-08 |  | A | C | -0.008 | 0.009 | 0.377 | 5.29267E-05 | 31.895 |
| LS-BMD | GERD | rs7206608 | 16 | 82872628 | G | C | 0.029 | 0.005 | 1.45761E-08 |  | G | C | 0.006 | 0.009 | 0.505 | 5.32811E-05 | 32.109 |
| LS-BMD | GERD | rs7241572 | 18 | 77580712 | A | G | 0.037 | 0.006 | 9.49336E-10 |  | A | G | 0.010 | 0.011 | 0.407 | 6.21038E-05 | 37.426 |
| LS-BMD | GERD | rs7527682 | 1 | 189172684 | G | A | -0.027 | 0.005 | 3.12831E-08 |  | G | A | -0.026 | 0.009 | 0.005 | 5.08206E-05 | 30.626 |
| LS-BMD | GERD | rs7541875 | 1 | 190957589 | G | A | 0.027 | 0.005 | 1.61213E-08 |  | G | A | 0.026 | 0.009 | 0.005 | 5.29562E-05 | 31.913 |
| LS-BMD | GERD | rs7600261 | 2 | 212622818 | T | C | 0.034 | 0.005 | 9.47327E-11 |  | T | C | 0.008 | 0.010 | 0.429 | 6.95717E-05 | 41.927 |
| LS-BMD | GERD | rs7612999 | 3 | 35678337 | A | G | 0.031 | 0.006 | 4.89824E-08 |  | A | G | 0.008 | 0.010 | 0.464 | 4.93779E-05 | 29.757 |
| LS-BMD | GERD | rs761777 | 10 | 134938075 | G | A | 0.035 | 0.006 | 4.71455E-10 |  | G | A | 0.010 | 0.010 | 0.336 | 6.43697E-05 | 38.792 |
| LS-BMD | GERD | rs7675588 | 4 | 80734978 | A | C | -0.034 | 0.006 | 1.80264E-08 |  | A | C | 0.003 | 0.011 | 0.773 | 5.2596E-05 | 31.696 |
| LS-BMD | GERD | rs7685686 | 4 | 3207142 | G | A | -0.028 | 0.005 | 1.14427E-08 |  | G | A | 0.002 | 0.009 | 0.849 | 5.40613E-05 | 32.579 |
| LS-BMD | GERD | rs773109 | 12 | 56390636 | A | G | -0.038 | 0.005 | 8.71365E-14 |  | A | G | -0.013 | 0.011 | 0.264 | 9.23206E-05 | 55.638 |
| LS-BMD | GERD | rs7942368 | 11 | 76465362 | T | C | -0.034 | 0.006 | 9.54135E-09 |  | T | C | 0.007 | 0.010 | 0.489 | 5.46475E-05 | 32.932 |
| LS-BMD | GERD | rs903678 | 1 | 201809918 | A | G | 0.028 | 0.005 | 4.89091E-08 |  | A | G | -0.011 | 0.009 | 0.258 | 4.93824E-05 | 29.759 |
| LS-BMD | GERD | rs903959 | 8 | 142630782 | A | T | 0.029 | 0.005 | 2.9893E-09 |  | A | T | 0.004 | 0.009 | 0.681 | 5.83945E-05 | 35.191 |
| LS-BMD | GERD | rs9372625 | 6 | 98344031 | A | G | -0.038 | 0.005 | 2.61818E-14 |  | A | G | 0.006 | 0.009 | 0.498 | 9.62435E-05 | 58.002 |
| LS-BMD | GERD | rs9373363 | 6 | 143150043 | G | A | -0.033 | 0.006 | 4.13457E-09 |  | G | A | 0.011 | 0.010 | 0.306 | 5.73464E-05 | 34.559 |
| LS-BMD | GERD | rs9396740 | 6 | 17023108 | A | G | -0.031 | 0.006 | 1.46562E-08 |  | A | G | 0.011 | 0.010 | 0.297 | 5.32631E-05 | 32.098 |
| LS-BMD | GERD | rs942065 | 14 | 94032065 | A | G | 0.031 | 0.005 | 8.44559E-10 |  | A | G | -0.011 | 0.009 | 0.246 | 6.24824E-05 | 37.654 |
| LS-BMD | GERD | rs9517313 | 13 | 99105892 | C | G | 0.033 | 0.005 | 2.04786E-11 |  | C | G | -0.003 | 0.009 | 0.787 | 7.45453E-05 | 44.925 |
| LS-BMD | GERD | rs9529055 | 13 | 66957533 | A | G | 0.027 | 0.005 | 3.10549E-08 |  | A | G | -0.006 | 0.009 | 0.530 | 5.0844E-05 | 30.640 |
| LS-BMD | GERD | rs9542729 | 13 | 31833578 | G | C | -0.036 | 0.006 | 1.41205E-09 |  | G | C | 0.009 | 0.011 | 0.454 | 6.08192E-05 | 36.652 |
| LS-BMD | GERD | rs9615905 | 22 | 48875699 | T | C | 0.028 | 0.005 | 1.21233E-08 |  | T | C | -0.024 | 0.010 | 0.017 | 5.38749E-05 | 32.467 |
| LS-BMD | GERD | rs9636202 | 19 | 18449238 | A | G | -0.035 | 0.005 | 1.51022E-10 |  | A | G | 0.015 | 0.010 | 0.141 | 6.80594E-05 | 41.016 |
| LS-BMD | GERD | rs9940128 | 16 | 53800754 | A | G | 0.033 | 0.005 | 8.05749E-12 |  | A | G | 0.008 | 0.009 | 0.385 | 7.75772E-05 | 46.752 |
| H-BMD | GERD | rs10010963 | 4 | 159839313 | T | C | -0.027 | 0.005 | 4.91779E-08 |  | T | C | -0.003 | 0.002 | 0.087 | 4.93648E-05 | 29.749 |
| H-BMD | GERD | rs1011407 | 2 | 60665768 | G | A | -0.042 | 0.007 | 1.09343E-08 |  | G | A | 0.007 | 0.003 | 0.015 | 5.42079E-05 | 32.668 |
| H-BMD | GERD | rs10133111 | 14 | 103377321 | A | G | 0.042 | 0.007 | 1.35338E-10 |  | A | G | 0.005 | 0.003 | 0.086 | 6.84149E-05 | 41.230 |
| H-BMD | GERD | rs1021363 | 10 | 106610839 | G | A | -0.031 | 0.005 | 5.09976E-10 |  | G | A | 0.003 | 0.002 | 0.200 | 6.41154E-05 | 38.639 |
| H-BMD | GERD | rs10837002 | 11 | 38565727 | G | C | 0.028 | 0.005 | 4.02893E-08 |  | G | C | -0.004 | 0.002 | 0.110 | 5.00063E-05 | 30.135 |
| H-BMD | GERD | rs11762636 | 7 | 2061111 | A | C | -0.051 | 0.006 | 1.88235E-16 |  | A | C | 0.007 | 0.002 | 0.005 | 0.00011237 | 67.722 |
| H-BMD | GERD | rs11953061 | 5 | 120144025 | T | C | 0.028 | 0.005 | 3.09949E-08 |  | T | C | -0.003 | 0.002 | 0.018 | 5.08505E-05 | 30.644 |
| H-BMD | GERD | rs12357321 | 10 | 21790476 | A | G | 0.032 | 0.005 | 1.33325E-09 |  | A | G | -0.002 | 0.002 | 0.330 | 6.10048E-05 | 36.764 |
| H-BMD | GERD | rs12453010 | 17 | 50316131 | T | C | 0.030 | 0.005 | 1.74606E-09 |  | T | C | -0.002 | 0.002 | 0.270 | 6.01325E-05 | 36.238 |
| H-BMD | GERD | rs12598916 | 16 | 60658751 | G | C | -0.033 | 0.005 | 6.8729E-10 |  | G | C | -0.002 | 0.002 | 0.160 | 6.31493E-05 | 38.056 |
| H-BMD | GERD | rs12967855 | 18 | 35138245 | G | A | -0.037 | 0.005 | 1.08793E-12 |  | G | A | 0.005 | 0.002 | 0.013 | 8.40922E-05 | 50.678 |
| H-BMD | GERD | rs12997558 | 2 | 41704580 | A | G | 0.028 | 0.005 | 3.03941E-08 |  | A | G | 0.001 | 0.002 | 0.820 | 5.09135E-05 | 30.682 |
| H-BMD | GERD | rs1334297 | 13 | 58335375 | A | G | -0.039 | 0.005 | 1.1413E-12 |  | A | G | -0.001 | 0.002 | 0.430 | 8.39364E-05 | 50.585 |
| H-BMD | GERD | rs13409451 | 2 | 144257639 | G | A | -0.028 | 0.005 | 1.92943E-08 |  | G | A | -0.005 | 0.002 | 0.027 | 5.23772E-05 | 31.564 |
| H-BMD | GERD | rs1431196 | 18 | 50832102 | G | A | 0.032 | 0.005 | 2.65461E-11 |  | G | A | -0.003 | 0.002 | 0.190 | 7.37023E-05 | 44.416 |
| H-BMD | GERD | rs1479405 | 12 | 15387519 | T | C | 0.031 | 0.005 | 9.85213E-10 |  | T | C | 0.006 | 0.002 | 0.035 | 6.19839E-05 | 37.354 |
| H-BMD | GERD | rs1510719 | 4 | 140938116 | C | T | -0.039 | 0.005 | 3.83619E-15 |  | C | T | -0.002 | 0.002 | 0.500 | 0.000102515 | 61.782 |
| H-BMD | GERD | rs1592757 | 5 | 103889998 | C | G | 0.031 | 0.005 | 5.9975E-10 |  | C | G | -0.004 | 0.002 | 0.006 | 6.35905E-05 | 38.322 |
| H-BMD | GERD | rs1596747 | 2 | 193802478 | G | A | 0.031 | 0.005 | 1.00344E-10 |  | G | A | 0.002 | 0.002 | 0.500 | 6.93855E-05 | 41.815 |
| H-BMD | GERD | rs1883842 | 20 | 41223062 | G | T | 0.031 | 0.005 | 9.26766E-09 |  | G | T | 0.001 | 0.002 | 0.690 | 5.47412E-05 | 32.989 |
| H-BMD | GERD | rs1937450 | 1 | 66478840 | G | T | 0.032 | 0.005 | 7.06806E-11 |  | G | T | 0.002 | 0.002 | 0.680 | 7.05223E-05 | 42.500 |
| H-BMD | GERD | rs2016933 | 3 | 65653157 | G | C | -0.031 | 0.005 | 1.04275E-08 |  | G | C | 0.003 | 0.002 | 0.100 | 5.43609E-05 | 32.760 |
| H-BMD | GERD | rs2023878 | 19 | 18834124 | T | C | -0.036 | 0.006 | 3.03501E-09 |  | T | C | 0.002 | 0.002 | 0.240 | 5.83456E-05 | 35.161 |
| H-BMD | GERD | rs2043539 | 7 | 12253880 | A | G | 0.027 | 0.005 | 2.24042E-08 |  | A | G | 0.005 | 0.002 | 0.039 | 5.18955E-05 | 31.274 |
| H-BMD | GERD | rs2106353 | 7 | 126506598 | T | G | 0.037 | 0.006 | 1.37177E-10 |  | T | G | -0.004 | 0.002 | 0.024 | 6.83712E-05 | 41.203 |
| H-BMD | GERD | rs215614 | 7 | 32347335 | A | G | -0.033 | 0.005 | 4.08413E-11 |  | A | G | -0.002 | 0.002 | 0.170 | 7.23027E-05 | 43.573 |
| H-BMD | GERD | rs2164300 | 4 | 67813017 | T | C | -0.026 | 0.005 | 4.13352E-08 |  | T | C | 0.000 | 0.002 | 0.390 | 4.99239E-05 | 30.086 |
| H-BMD | GERD | rs2396133 | 7 | 109197067 | G | A | 0.029 | 0.005 | 1.10889E-09 |  | G | A | 0.005 | 0.002 | 0.013 | 6.16008E-05 | 37.123 |
| H-BMD | GERD | rs2396766 | 7 | 114318071 | A | G | 0.032 | 0.005 | 2.33507E-11 |  | A | G | 0.005 | 0.002 | 0.059 | 7.41186E-05 | 44.667 |
| H-BMD | GERD | rs2734839 | 11 | 113286490 | T | C | -0.028 | 0.005 | 8.78537E-09 |  | T | C | 0.000 | 0.002 | 0.750 | 5.49136E-05 | 33.093 |
| H-BMD | GERD | rs2744961 | 6 | 34655000 | T | C | 0.029 | 0.005 | 5.81126E-09 |  | T | C | 0.001 | 0.002 | 0.480 | 5.62475E-05 | 33.897 |
| H-BMD | GERD | rs2782641 | 1 | 44013355 | A | G | 0.027 | 0.005 | 4.32643E-08 |  | A | G | 0.004 | 0.002 | 0.180 | 4.9777E-05 | 29.997 |
| H-BMD | GERD | rs2834005 | 21 | 34291708 | C | T | 0.030 | 0.005 | 9.42041E-09 |  | C | T | 0.002 | 0.002 | 0.580 | 5.46885E-05 | 32.957 |
| H-BMD | GERD | rs2838771 | 21 | 46501576 | C | G | -0.028 | 0.005 | 2.90964E-08 |  | C | G | -0.004 | 0.002 | 0.076 | 5.10538E-05 | 30.767 |
| H-BMD | GERD | rs324769 | 12 | 83969240 | T | C | -0.027 | 0.005 | 3.04775E-08 |  | T | C | -0.001 | 0.002 | 0.410 | 5.09044E-05 | 30.677 |
| H-BMD | GERD | rs329122 | 5 | 133864599 | A | G | -0.029 | 0.005 | 3.05485E-09 |  | A | G | -0.003 | 0.002 | 0.220 | 5.83244E-05 | 35.148 |
| H-BMD | GERD | rs3766823 | 1 | 32197257 | A | G | 0.039 | 0.006 | 7.09316E-10 |  | A | G | 0.002 | 0.002 | 0.120 | 6.30472E-05 | 37.995 |
| H-BMD | GERD | rs3793577 | 9 | 23737627 | G | A | 0.027 | 0.005 | 2.49436E-08 |  | G | A | -0.003 | 0.002 | 0.180 | 5.15497E-05 | 31.066 |
| H-BMD | GERD | rs3863241 | 8 | 73890335 | T | C | 0.032 | 0.005 | 1.48799E-11 |  | T | C | -0.001 | 0.002 | 0.680 | 7.55829E-05 | 45.550 |
| H-BMD | GERD | rs4300861 | 2 | 22549441 | T | C | 0.031 | 0.005 | 5.431E-10 |  | T | C | -0.003 | 0.002 | 0.250 | 6.39117E-05 | 38.516 |
| H-BMD | GERD | rs4382592 | 9 | 134870755 | G | T | -0.030 | 0.005 | 8.19917E-09 |  | G | T | -0.002 | 0.002 | 0.590 | 5.51365E-05 | 33.227 |
| H-BMD | GERD | rs4713692 | 6 | 33807638 | T | C | -0.028 | 0.005 | 3.06507E-08 |  | T | C | -0.001 | 0.002 | 0.910 | 5.08866E-05 | 30.666 |
| H-BMD | GERD | rs569356 | 1 | 29136686 | G | A | -0.038 | 0.007 | 4.07005E-08 |  | G | A | -0.004 | 0.003 | 0.037 | 4.99736E-05 | 30.116 |
| H-BMD | GERD | rs6711584 | 2 | 104421692 | A | G | 0.032 | 0.005 | 2.65583E-11 |  | A | G | 0.000 | 0.002 | 0.880 | 7.37009E-05 | 44.416 |
| H-BMD | GERD | rs6722661 | 2 | 100806588 | A | G | -0.032 | 0.005 | 1.14623E-10 |  | A | G | 0.002 | 0.002 | 0.036 | 6.89539E-05 | 41.555 |
| H-BMD | GERD | rs6780459 | 3 | 104624105 | T | A | 0.031 | 0.006 | 3.13993E-08 |  | T | A | -0.002 | 0.002 | 0.400 | 5.08086E-05 | 30.619 |
| H-BMD | GERD | rs7032155 | 9 | 122672771 | A | C | 0.028 | 0.005 | 1.62686E-08 |  | A | C | 0.001 | 0.002 | 0.990 | 5.29267E-05 | 31.895 |
| H-BMD | GERD | rs7206608 | 16 | 82872628 | G | C | 0.029 | 0.005 | 1.45761E-08 |  | G | C | 0.000 | 0.002 | 0.710 | 5.32811E-05 | 32.109 |
| H-BMD | GERD | rs7241572 | 18 | 77580712 | A | G | 0.037 | 0.006 | 9.49336E-10 |  | A | G | 0.006 | 0.002 | 0.002 | 6.21038E-05 | 37.426 |
| H-BMD | GERD | rs7527682 | 1 | 189172684 | G | A | -0.027 | 0.005 | 3.12831E-08 |  | G | A | 0.002 | 0.002 | 0.054 | 5.08206E-05 | 30.626 |
| H-BMD | GERD | rs7541875 | 1 | 190957589 | G | A | 0.027 | 0.005 | 1.61213E-08 |  | G | A | 0.000 | 0.002 | 0.690 | 5.29562E-05 | 31.913 |
| H-BMD | GERD | rs7600261 | 2 | 212622818 | T | C | 0.034 | 0.005 | 9.47327E-11 |  | T | C | -0.002 | 0.002 | 0.130 | 6.95717E-05 | 41.927 |
| H-BMD | GERD | rs7612999 | 3 | 35678337 | A | G | 0.031 | 0.006 | 4.89824E-08 |  | A | G | -0.004 | 0.002 | 0.210 | 4.93779E-05 | 29.757 |
| H-BMD | GERD | rs761777 | 10 | 134938075 | G | A | 0.035 | 0.006 | 4.71455E-10 |  | G | A | -0.001 | 0.002 | 0.390 | 6.43697E-05 | 38.792 |
| H-BMD | GERD | rs7675588 | 4 | 80734978 | A | C | -0.034 | 0.006 | 1.80264E-08 |  | A | C | 0.003 | 0.002 | 0.180 | 5.2596E-05 | 31.696 |
| H-BMD | GERD | rs7685686 | 4 | 3207142 | G | A | -0.028 | 0.005 | 1.14427E-08 |  | G | A | 0.001 | 0.002 | 0.980 | 5.40613E-05 | 32.579 |
| H-BMD | GERD | rs7942368 | 11 | 76465362 | T | C | -0.034 | 0.006 | 9.54135E-09 |  | T | C | -0.004 | 0.002 | 0.130 | 5.46475E-05 | 32.932 |
| H-BMD | GERD | rs903678 | 1 | 201809918 | A | G | 0.028 | 0.005 | 4.89091E-08 |  | A | G | -0.002 | 0.002 | 0.230 | 4.93824E-05 | 29.759 |
| H-BMD | GERD | rs903959 | 8 | 142630782 | A | T | 0.029 | 0.005 | 2.9893E-09 |  | A | T | 0.003 | 0.002 | 0.310 | 5.83945E-05 | 35.191 |
| H-BMD | GERD | rs9372625 | 6 | 98344031 | A | G | -0.038 | 0.005 | 2.61818E-14 |  | A | G | 0.000 | 0.002 | 0.890 | 9.62435E-05 | 58.002 |
| H-BMD | GERD | rs9373363 | 6 | 143150043 | G | A | -0.033 | 0.006 | 4.13457E-09 |  | G | A | -0.003 | 0.002 | 0.640 | 5.73464E-05 | 34.559 |
| H-BMD | GERD | rs9396740 | 6 | 17023108 | A | G | -0.031 | 0.006 | 1.46562E-08 |  | A | G | -0.002 | 0.002 | 0.380 | 5.32631E-05 | 32.098 |
| H-BMD | GERD | rs942065 | 14 | 94032065 | A | G | 0.031 | 0.005 | 8.44559E-10 |  | A | G | -0.003 | 0.002 | 0.760 | 6.24824E-05 | 37.654 |
| H-BMD | GERD | rs9517313 | 13 | 99105892 | C | G | 0.033 | 0.005 | 2.04786E-11 |  | C | G | -0.004 | 0.002 | 0.120 | 7.45453E-05 | 44.925 |
| H-BMD | GERD | rs9529055 | 13 | 66957533 | A | G | 0.027 | 0.005 | 3.10549E-08 |  | A | G | 0.002 | 0.002 | 0.240 | 5.0844E-05 | 30.640 |
| H-BMD | GERD | rs9615905 | 22 | 48875699 | T | C | 0.028 | 0.005 | 1.21233E-08 |  | T | C | -0.001 | 0.002 | 0.350 | 5.38749E-05 | 32.467 |
| H-BMD | GERD | rs9636202 | 19 | 18449238 | A | G | -0.035 | 0.005 | 1.51022E-10 |  | A | G | 0.000 | 0.002 | 0.640 | 6.80594E-05 | 41.016 |
| UF-BMD | GERD | rs10010963 | 4 | 159839313 | T | C | -0.027 | 0.005 | 4.91779E-08 |  | T | C | -0.006 | 0.011 | 0.567 | 4.93648E-05 | 29.749 |
| UF-BMD | GERD | rs1011407 | 2 | 60665768 | G | A | -0.042 | 0.007 | 1.09343E-08 |  | G | A | -0.012 | 0.016 | 0.465 | 5.42079E-05 | 32.668 |
| UF-BMD | GERD | rs10133111 | 14 | 103377321 | A | G | 0.042 | 0.007 | 1.35338E-10 |  | A | G | 0.014 | 0.015 | 0.349 | 6.84149E-05 | 41.230 |
| UF-BMD | GERD | rs1021363 | 10 | 106610839 | G | A | -0.031 | 0.005 | 5.09976E-10 |  | G | A | -0.014 | 0.011 | 0.219 | 6.41154E-05 | 38.639 |
| UF-BMD | GERD | rs10837002 | 11 | 38565727 | G | C | 0.028 | 0.005 | 4.02893E-08 |  | G | C | -0.005 | 0.011 | 0.650 | 5.00063E-05 | 30.135 |
| UF-BMD | GERD | rs11762636 | 7 | 2061111 | A | C | -0.051 | 0.006 | 1.88235E-16 |  | A | C | -0.007 | 0.014 | 0.633 | 0.00011237 | 67.722 |
| UF-BMD | GERD | rs11953061 | 5 | 120144025 | T | C | 0.028 | 0.005 | 3.09949E-08 |  | T | C | 0.013 | 0.011 | 0.247 | 5.08505E-05 | 30.644 |
| UF-BMD | GERD | rs12204714 | 6 | 152235339 | T | C | -0.029 | 0.005 | 7.9241E-09 |  | T | C | 0.011 | 0.011 | 0.316 | 5.52467E-05 | 33.294 |
| UF-BMD | GERD | rs12357321 | 10 | 21790476 | A | G | 0.032 | 0.005 | 1.33325E-09 |  | A | G | -0.006 | 0.011 | 0.626 | 6.10048E-05 | 36.764 |
| UF-BMD | GERD | rs12453010 | 17 | 50316131 | T | C | 0.030 | 0.005 | 1.74606E-09 |  | T | C | 0.003 | 0.011 | 0.784 | 6.01325E-05 | 36.238 |
| UF-BMD | GERD | rs12598916 | 16 | 60658751 | G | C | -0.033 | 0.005 | 6.8729E-10 |  | G | C | -0.002 | 0.012 | 0.840 | 6.31493E-05 | 38.056 |
| UF-BMD | GERD | rs12967855 | 18 | 35138245 | G | A | -0.037 | 0.005 | 1.08793E-12 |  | G | A | 0.015 | 0.011 | 0.189 | 8.40922E-05 | 50.678 |
| UF-BMD | GERD | rs12997558 | 2 | 41704580 | A | G | 0.028 | 0.005 | 3.03941E-08 |  | A | G | 0.016 | 0.011 | 0.150 | 5.09135E-05 | 30.682 |
| UF-BMD | GERD | rs13107325 | 4 | 103188709 | T | C | 0.070 | 0.009 | 2.19938E-14 |  | T | C | 0.013 | 0.023 | 0.574 | 9.68122E-05 | 58.345 |
| UF-BMD | GERD | rs1334297 | 13 | 58335375 | A | G | -0.039 | 0.005 | 1.1413E-12 |  | A | G | 0.007 | 0.012 | 0.568 | 8.39364E-05 | 50.585 |
| UF-BMD | GERD | rs13409451 | 2 | 144257639 | G | A | -0.028 | 0.005 | 1.92943E-08 |  | G | A | -0.014 | 0.011 | 0.216 | 5.23772E-05 | 31.564 |
| UF-BMD | GERD | rs1431196 | 18 | 50832102 | G | A | 0.032 | 0.005 | 2.65461E-11 |  | G | A | 0.003 | 0.011 | 0.791 | 7.37023E-05 | 44.416 |
| UF-BMD | GERD | rs1479405 | 12 | 15387519 | T | C | 0.031 | 0.005 | 9.85213E-10 |  | T | C | 0.019 | 0.011 | 0.085 | 6.19839E-05 | 37.354 |
| UF-BMD | GERD | rs1510719 | 4 | 140938116 | C | T | -0.039 | 0.005 | 3.83619E-15 |  | C | T | -0.006 | 0.011 | 0.602 | 0.000102515 | 61.782 |
| UF-BMD | GERD | rs1592757 | 5 | 103889998 | C | G | 0.031 | 0.005 | 5.9975E-10 |  | C | G | -0.016 | 0.011 | 0.150 | 6.35905E-05 | 38.322 |
| UF-BMD | GERD | rs1596747 | 2 | 193802478 | G | A | 0.031 | 0.005 | 1.00344E-10 |  | G | A | -0.017 | 0.011 | 0.114 | 6.93855E-05 | 41.815 |
| UF-BMD | GERD | rs1716171 | 12 | 123716376 | T | C | 0.038 | 0.006 | 7.82528E-11 |  | T | C | 0.002 | 0.013 | 0.910 | 7.01925E-05 | 42.301 |
| UF-BMD | GERD | rs17379561 | 1 | 98340139 | T | A | 0.053 | 0.007 | 1.07622E-14 |  | T | A | -0.023 | 0.014 | 0.107 | 9.91454E-05 | 59.751 |
| UF-BMD | GERD | rs1883842 | 20 | 41223062 | G | T | 0.031 | 0.005 | 9.26766E-09 |  | G | T | -0.003 | 0.012 | 0.787 | 5.47412E-05 | 32.989 |
| UF-BMD | GERD | rs1937450 | 1 | 66478840 | G | T | 0.032 | 0.005 | 7.06806E-11 |  | G | T | 0.001 | 0.011 | 0.952 | 7.05223E-05 | 42.500 |
| UF-BMD | GERD | rs2016933 | 3 | 65653157 | G | C | -0.031 | 0.005 | 1.04275E-08 |  | G | C | 0.009 | 0.012 | 0.428 | 5.43609E-05 | 32.760 |
| UF-BMD | GERD | rs2023878 | 19 | 18834124 | T | C | -0.036 | 0.006 | 3.03501E-09 |  | T | C | 0.009 | 0.014 | 0.521 | 5.83456E-05 | 35.161 |
| UF-BMD | GERD | rs2043539 | 7 | 12253880 | A | G | 0.027 | 0.005 | 2.24042E-08 |  | A | G | -0.003 | 0.011 | 0.810 | 5.18955E-05 | 31.274 |
| UF-BMD | GERD | rs2106353 | 7 | 126506598 | T | G | 0.037 | 0.006 | 1.37177E-10 |  | T | G | -0.012 | 0.013 | 0.348 | 6.83712E-05 | 41.203 |
| UF-BMD | GERD | rs215614 | 7 | 32347335 | A | G | -0.033 | 0.005 | 4.08413E-11 |  | A | G | 0.006 | 0.011 | 0.616 | 7.23027E-05 | 43.573 |
| UF-BMD | GERD | rs2164300 | 4 | 67813017 | T | C | -0.026 | 0.005 | 4.13352E-08 |  | T | C | 0.014 | 0.011 | 0.201 | 4.99239E-05 | 30.086 |
| UF-BMD | GERD | rs2240326 | 3 | 50128386 | A | G | -0.047 | 0.005 | 1.13214E-22 |  | A | G | -0.012 | 0.011 | 0.273 | 0.000159331 | 96.029 |
| UF-BMD | GERD | rs2396133 | 7 | 109197067 | G | A | 0.029 | 0.005 | 1.10889E-09 |  | G | A | 0.011 | 0.011 | 0.318 | 6.16008E-05 | 37.123 |
| UF-BMD | GERD | rs2396766 | 7 | 114318071 | A | G | 0.032 | 0.005 | 2.33507E-11 |  | A | G | -0.003 | 0.011 | 0.758 | 7.41186E-05 | 44.667 |
| UF-BMD | GERD | rs2734839 | 11 | 113286490 | T | C | -0.028 | 0.005 | 8.78537E-09 |  | T | C | 0.006 | 0.011 | 0.574 | 5.49136E-05 | 33.093 |
| UF-BMD | GERD | rs2744961 | 6 | 34655000 | T | C | 0.029 | 0.005 | 5.81126E-09 |  | T | C | -0.007 | 0.011 | 0.556 | 5.62475E-05 | 33.897 |
| UF-BMD | GERD | rs2782641 | 1 | 44013355 | A | G | 0.027 | 0.005 | 4.32643E-08 |  | A | G | 0.011 | 0.011 | 0.328 | 4.9777E-05 | 29.997 |
| UF-BMD | GERD | rs2815749 | 1 | 72814783 | G | A | 0.039 | 0.006 | 1.07359E-10 |  | G | A | 0.016 | 0.013 | 0.227 | 6.9166E-05 | 41.682 |
| UF-BMD | GERD | rs2834005 | 21 | 34291708 | C | T | 0.030 | 0.005 | 9.42041E-09 |  | C | T | 0.003 | 0.012 | 0.772 | 5.46885E-05 | 32.957 |
| UF-BMD | GERD | rs2838771 | 21 | 46501576 | C | G | -0.028 | 0.005 | 2.90964E-08 |  | C | G | 0.016 | 0.011 | 0.146 | 5.10538E-05 | 30.767 |
| UF-BMD | GERD | rs324769 | 12 | 83969240 | T | C | -0.027 | 0.005 | 3.04775E-08 |  | T | C | 0.001 | 0.011 | 0.925 | 5.09044E-05 | 30.677 |
| UF-BMD | GERD | rs329122 | 5 | 133864599 | A | G | -0.029 | 0.005 | 3.05485E-09 |  | A | G | 0.010 | 0.011 | 0.344 | 5.83244E-05 | 35.148 |
| UF-BMD | GERD | rs3766823 | 1 | 32197257 | A | G | 0.039 | 0.006 | 7.09316E-10 |  | A | G | 0.017 | 0.014 | 0.217 | 6.30472E-05 | 37.995 |
| UF-BMD | GERD | rs3793577 | 9 | 23737627 | G | A | 0.027 | 0.005 | 2.49436E-08 |  | G | A | 0.007 | 0.011 | 0.494 | 5.15497E-05 | 31.066 |
| UF-BMD | GERD | rs3863241 | 8 | 73890335 | T | C | 0.032 | 0.005 | 1.48799E-11 |  | T | C | 0.009 | 0.011 | 0.397 | 7.55829E-05 | 45.550 |
| UF-BMD | GERD | rs4300861 | 2 | 22549441 | T | C | 0.031 | 0.005 | 5.431E-10 |  | T | C | 0.002 | 0.011 | 0.866 | 6.39117E-05 | 38.516 |
| UF-BMD | GERD | rs4382592 | 9 | 134870755 | G | T | -0.030 | 0.005 | 8.19917E-09 |  | G | T | 0.005 | 0.011 | 0.650 | 5.51365E-05 | 33.227 |
| UF-BMD | GERD | rs4713692 | 6 | 33807638 | T | C | -0.028 | 0.005 | 3.06507E-08 |  | T | C | -0.021 | 0.011 | 0.055 | 5.08866E-05 | 30.666 |
| UF-BMD | GERD | rs569356 | 1 | 29136686 | G | A | -0.038 | 0.007 | 4.07005E-08 |  | G | A | 0.007 | 0.016 | 0.653 | 4.99736E-05 | 30.116 |
| UF-BMD | GERD | rs6711584 | 2 | 104421692 | A | G | 0.032 | 0.005 | 2.65583E-11 |  | A | G | 0.009 | 0.011 | 0.398 | 7.37009E-05 | 44.416 |
| UF-BMD | GERD | rs6722661 | 2 | 100806588 | A | G | -0.032 | 0.005 | 1.14623E-10 |  | A | G | -0.005 | 0.011 | 0.632 | 6.89539E-05 | 41.555 |
| UF-BMD | GERD | rs6780459 | 3 | 104624105 | T | A | 0.031 | 0.006 | 3.13993E-08 |  | T | A | -0.001 | 0.012 | 0.952 | 5.08086E-05 | 30.619 |
| UF-BMD | GERD | rs7032155 | 9 | 122672771 | A | C | 0.028 | 0.005 | 1.62686E-08 |  | A | C | 0.009 | 0.011 | 0.406 | 5.29267E-05 | 31.895 |
| UF-BMD | GERD | rs7206608 | 16 | 82872628 | G | C | 0.029 | 0.005 | 1.45761E-08 |  | G | C | -0.002 | 0.011 | 0.873 | 5.32811E-05 | 32.109 |
| UF-BMD | GERD | rs7241572 | 18 | 77580712 | A | G | 0.037 | 0.006 | 9.49336E-10 |  | A | G | -0.021 | 0.013 | 0.110 | 6.21038E-05 | 37.426 |
| UF-BMD | GERD | rs7527682 | 1 | 189172684 | G | A | -0.027 | 0.005 | 3.12831E-08 |  | G | A | 0.003 | 0.011 | 0.767 | 5.08206E-05 | 30.626 |
| UF-BMD | GERD | rs7541875 | 1 | 190957589 | G | A | 0.027 | 0.005 | 1.61213E-08 |  | G | A | -0.009 | 0.011 | 0.415 | 5.29562E-05 | 31.913 |
| UF-BMD | GERD | rs7600261 | 2 | 212622818 | T | C | 0.034 | 0.005 | 9.47327E-11 |  | T | C | -0.009 | 0.012 | 0.452 | 6.95717E-05 | 41.927 |
| UF-BMD | GERD | rs7612999 | 3 | 35678337 | A | G | 0.031 | 0.006 | 4.89824E-08 |  | A | G | -0.001 | 0.012 | 0.913 | 4.93779E-05 | 29.757 |
| UF-BMD | GERD | rs761777 | 10 | 134938075 | G | A | 0.035 | 0.006 | 4.71455E-10 |  | G | A | -0.005 | 0.013 | 0.709 | 6.43697E-05 | 38.792 |
| UF-BMD | GERD | rs7675588 | 4 | 80734978 | A | C | -0.034 | 0.006 | 1.80264E-08 |  | A | C | 0.009 | 0.013 | 0.487 | 5.2596E-05 | 31.696 |
| UF-BMD | GERD | rs7685686 | 4 | 3207142 | G | A | -0.028 | 0.005 | 1.14427E-08 |  | G | A | -0.010 | 0.011 | 0.344 | 5.40613E-05 | 32.579 |
| UF-BMD | GERD | rs773109 | 12 | 56374695 | A | G | -0.038 | 0.005 | 8.71365E-14 |  | A | G | -0.016 | 0.011 | 0.159 | 9.23206E-05 | 55.638 |
| UF-BMD | GERD | rs7942368 | 11 | 76465362 | T | C | -0.034 | 0.006 | 9.54135E-09 |  | T | C | 0.022 | 0.013 | 0.083 | 5.46475E-05 | 32.932 |
| UF-BMD | GERD | rs903678 | 1 | 201809918 | A | G | 0.028 | 0.005 | 4.89091E-08 |  | A | G | 0.002 | 0.011 | 0.854 | 4.93824E-05 | 29.759 |
| UF-BMD | GERD | rs903959 | 8 | 142630782 | A | T | 0.029 | 0.005 | 2.9893E-09 |  | A | T | 0.014 | 0.011 | 0.206 | 5.83945E-05 | 35.191 |
| UF-BMD | GERD | rs9372625 | 6 | 98344031 | A | G | -0.038 | 0.005 | 2.61818E-14 |  | A | G | 0.017 | 0.011 | 0.117 | 9.62435E-05 | 58.002 |
| UF-BMD | GERD | rs9373363 | 6 | 143150043 | G | A | -0.033 | 0.006 | 4.13457E-09 |  | G | A | -0.005 | 0.012 | 0.679 | 5.73464E-05 | 34.559 |
| UF-BMD | GERD | rs9396740 | 6 | 17023108 | A | G | -0.031 | 0.006 | 1.46562E-08 |  | A | G | 0.005 | 0.013 | 0.689 | 5.32631E-05 | 32.098 |
| UF-BMD | GERD | rs942065 | 14 | 94032065 | A | G | 0.031 | 0.005 | 8.44559E-10 |  | A | G | -0.002 | 0.011 | 0.876 | 6.24824E-05 | 37.654 |
| UF-BMD | GERD | rs9517313 | 13 | 99105892 | C | G | 0.033 | 0.005 | 2.04786E-11 |  | C | G | -0.026 | 0.011 | 0.016 | 7.45453E-05 | 44.925 |
| UF-BMD | GERD | rs9529055 | 13 | 66957533 | A | G | 0.027 | 0.005 | 3.10549E-08 |  | A | G | -0.020 | 0.011 | 0.055 | 5.0844E-05 | 30.640 |
| UF-BMD | GERD | rs9542729 | 13 | 31833578 | G | C | -0.036 | 0.006 | 1.41205E-09 |  | G | C | -0.001 | 0.014 | 0.949 | 6.08192E-05 | 36.652 |
| UF-BMD | GERD | rs9615905 | 22 | 48875699 | T | C | 0.028 | 0.005 | 1.21233E-08 |  | T | C | -0.003 | 0.011 | 0.802 | 5.38749E-05 | 32.467 |
| UF-BMD | GERD | rs9636202 | 19 | 18449238 | A | G | -0.035 | 0.005 | 1.51022E-10 |  | A | G | 0.008 | 0.012 | 0.537 | 6.80594E-05 | 41.016 |
| UF-BMD | GERD | rs9940128 | 16 | 53800754 | A | G | 0.033 | 0.005 | 8.05749E-12 |  | A | G | 0.028 | 0.011 | 0.007 | 7.75772E-05 | 46.752 |
| GERD | TB-BMD | rs1037011 | 12 | 107367225 | C | T | 0.040 | 0.006 | 1.54099E-12 |  | C | T | -0.007 | 0.005 | 0.149 | 0.000891745 | 50.234 |
| GERD | TB-BMD | rs10490046 | 2 | 40630678 | C | A | -0.043 | 0.007 | 1.434E-10 |  | C | A | -0.002 | 0.006 | 0.686 | 0.000727887 | 40.997 |
| GERD | TB-BMD | rs10493013 | 1 | 22703035 | C | T | 0.101 | 0.007 | 4.07474E-43 |  | C | T | -0.012 | 0.006 | 0.067 | 0.003318386 | 187.387 |
| GERD | TB-BMD | rs10777212 | 12 | 90334829 | T | G | 0.045 | 0.006 | 5.05126E-14 |  | T | G | -0.001 | 0.005 | 0.916 | 0.001007284 | 56.749 |
| GERD | TB-BMD | rs10788264 | 10 | 124015986 | A | G | -0.034 | 0.006 | 2.60597E-09 |  | A | G | 0.000 | 0.005 | 0.964 | 0.000624349 | 35.162 |
| GERD | TB-BMD | rs10832520 | 11 | 15816918 | A | T | 0.112 | 0.016 | 1.00092E-12 |  | A | T | -0.005 | 0.014 | 0.736 | 0.000896749 | 50.516 |
| GERD | TB-BMD | rs10931982 | 2 | 202832130 | C | T | 0.051 | 0.009 | 1.585E-08 |  | C | T | -0.001 | 0.006 | 0.878 | 0.000565733 | 31.859 |
| GERD | TB-BMD | rs11228240 | 11 | 68254328 | T | C | -0.083 | 0.007 | 1.71791E-35 |  | T | C | 0.010 | 0.005 | 0.073 | 0.002719187 | 153.459 |
| GERD | TB-BMD | rs1159798 | 10 | 54412493 | C | A | -0.043 | 0.007 | 1.014E-09 |  | C | A | -0.003 | 0.006 | 0.657 | 0.000666874 | 37.558 |
| GERD | TB-BMD | rs11745493 | 5 | 122847622 | G | A | -0.045 | 0.007 | 7.74462E-12 |  | G | A | -0.008 | 0.006 | 0.151 | 0.000832045 | 46.868 |
| GERD | TB-BMD | rs11898505 | 2 | 54684557 | G | A | -0.034 | 0.006 | 1.28201E-08 |  | G | A | 0.004 | 0.005 | 0.418 | 0.000576918 | 32.489 |
| GERD | TB-BMD | rs11904127 | 2 | 85484818 | A | G | -0.032 | 0.006 | 1.18201E-08 |  | A | G | -0.001 | 0.005 | 0.888 | 0.000573728 | 32.309 |
| GERD | TB-BMD | rs11910328 | 21 | 40350744 | A | G | -0.043 | 0.008 | 2.99302E-08 |  | A | G | 0.008 | 0.007 | 0.252 | 0.000551199 | 31.040 |
| GERD | TB-BMD | rs11934731 | 4 | 88849669 | A | G | -0.067 | 0.006 | 8.38687E-29 |  | A | G | -0.002 | 0.005 | 0.765 | 0.002164383 | 122.080 |
| GERD | TB-BMD | rs12044944 | 1 | 240592636 | T | C | 0.055 | 0.007 | 7.54223E-14 |  | T | C | -0.011 | 0.006 | 0.078 | 0.000991223 | 55.843 |
| GERD | TB-BMD | rs12258451 | 10 | 54431330 | G | C | -0.070 | 0.009 | 2.41213E-15 |  | G | C | 0.003 | 0.008 | 0.665 | 0.001104154 | 62.213 |
| GERD | TB-BMD | rs12442242 | 15 | 38341734 | G | A | 0.051 | 0.008 | 4.94106E-10 |  | G | A | 0.008 | 0.007 | 0.238 | 0.00068411 | 38.529 |
| GERD | TB-BMD | rs12534510 | 7 | 120732120 | C | A | 0.040 | 0.006 | 3.14775E-12 |  | C | A | -0.004 | 0.005 | 0.458 | 0.00085249 | 48.021 |
| GERD | TB-BMD | rs1286150 | 14 | 91464890 | C | T | 0.055 | 0.007 | 2.44174E-14 |  | C | T | -0.002 | 0.006 | 0.723 | 0.001031921 | 58.139 |
| GERD | TB-BMD | rs13204965 | 6 | 127167072 | C | A | -0.062 | 0.007 | 1.01508E-18 |  | C | A | 0.020 | 0.005 | 0.000 | 0.001387386 | 78.193 |
| GERD | TB-BMD | rs144691710 | 17 | 41826839 | G | A | 0.102 | 0.011 | 2.23924E-19 |  | G | A | -0.007 | 0.009 | 0.397 | 0.001437062 | 80.997 |
| GERD | TB-BMD | rs1452102 | 21 | 28773868 | G | T | 0.035 | 0.006 | 1.736E-09 |  | G | T | -0.008 | 0.005 | 0.111 | 0.000650461 | 36.633 |
| GERD | TB-BMD | rs1548607 | 7 | 50901491 | G | A | -0.036 | 0.007 | 4.18398E-08 |  | G | A | 0.006 | 0.005 | 0.238 | 0.000537164 | 30.249 |
| GERD | TB-BMD | rs2252865 | 1 | 8422676 | C | T | 0.033 | 0.006 | 4.71998E-08 |  | C | T | 0.006 | 0.005 | 0.249 | 0.000530676 | 29.883 |
| GERD | TB-BMD | rs2289410 | 2 | 42284110 | T | A | -0.049 | 0.009 | 2.00198E-08 |  | T | A | -0.005 | 0.007 | 0.456 | 0.000559578 | 31.512 |
| GERD | TB-BMD | rs2350085 | 2 | 202799604 | C | T | 0.064 | 0.009 | 3.7949E-14 |  | C | T | -0.006 | 0.007 | 0.446 | 0.001015682 | 57.223 |
| GERD | TB-BMD | rs3743347 | 15 | 67547301 | A | C | 0.052 | 0.007 | 1.75186E-14 |  | A | C | -0.016 | 0.006 | 0.005 | 0.00103391 | 58.251 |
| GERD | TB-BMD | rs3801387 | 7 | 120974765 | G | A | 0.135 | 0.006 | 1.15E-100 |  | G | A | -0.014 | 0.005 | 0.012 | 0.008056678 | 457.129 |
| GERD | TB-BMD | rs4757350 | 11 | 15714623 | T | C | -0.056 | 0.007 | 3.75405E-16 |  | T | C | 0.002 | 0.006 | 0.767 | 0.001185659 | 66.810 |
| GERD | TB-BMD | rs4846580 | 1 | 219897941 | A | G | 0.035 | 0.006 | 3.21299E-09 |  | A | G | -0.003 | 0.005 | 0.551 | 0.000628238 | 35.381 |
| GERD | TB-BMD | rs55781332 | 11 | 249131 | G | A | 0.055 | 0.007 | 8.07235E-16 |  | G | A | -0.002 | 0.006 | 0.685 | 0.001135799 | 63.998 |
| GERD | TB-BMD | rs56104760 | 1 | 22492887 | G | A | -0.075 | 0.007 | 7.37734E-24 |  | G | A | -0.005 | 0.006 | 0.417 | 0.001807204 | 101.897 |
| GERD | TB-BMD | rs6029130 | 20 | 39108334 | T | C | 0.035 | 0.006 | 3.503E-08 |  | T | C | 0.000 | 0.006 | 0.936 | 0.000541823 | 30.511 |
| GERD | TB-BMD | rs6040063 | 20 | 10643850 | G | A | -0.036 | 0.006 | 1.78098E-10 |  | G | A | -0.007 | 0.005 | 0.136 | 0.000729644 | 41.096 |
| GERD | TB-BMD | rs61884327 | 11 | 46823681 | C | T | 0.080 | 0.010 | 4.63447E-16 |  | C | T | 0.002 | 0.008 | 0.767 | 0.001161729 | 65.460 |
| GERD | TB-BMD | rs634277 | 11 | 86887931 | G | A | -0.061 | 0.006 | 2.15179E-23 |  | G | A | -0.002 | 0.005 | 0.762 | 0.001756181 | 99.015 |
| GERD | TB-BMD | rs6465511 | 7 | 96134115 | G | C | 0.074 | 0.006 | 1.02802E-34 |  | G | C | -0.007 | 0.005 | 0.159 | 0.002680769 | 151.285 |
| GERD | TB-BMD | rs6960249 | 7 | 96660132 | G | T | -0.033 | 0.006 | 1.44901E-08 |  | G | T | -0.005 | 0.005 | 0.336 | 0.000577273 | 32.509 |
| GERD | TB-BMD | rs7105860 | 11 | 27309956 | C | G | -0.047 | 0.006 | 2.35885E-15 |  | C | G | 0.015 | 0.005 | 0.002 | 0.001116651 | 62.918 |
| GERD | TB-BMD | rs725670 | 11 | 121913230 | A | G | -0.032 | 0.006 | 3.61202E-08 |  | A | G | 0.005 | 0.005 | 0.330 | 0.000528924 | 29.785 |
| GERD | TB-BMD | rs73349318 | 10 | 112245608 | T | A | 0.047 | 0.009 | 2.68201E-08 |  | T | A | 0.004 | 0.006 | 0.500 | 0.000547549 | 30.834 |
| GERD | TB-BMD | rs73719807 | 7 | 121220682 | C | A | 0.093 | 0.011 | 1.14288E-16 |  | C | A | -0.003 | 0.010 | 0.802 | 0.001210421 | 68.207 |
| GERD | TB-BMD | rs7548588 | 1 | 110482335 | C | T | 0.037 | 0.006 | 2.208E-10 |  | C | T | -0.015 | 0.005 | 0.002 | 0.000710857 | 40.037 |
| GERD | TB-BMD | rs757138 | 7 | 27989768 | G | T | 0.035 | 0.006 | 3.33403E-08 |  | G | T | -0.001 | 0.006 | 0.890 | 0.000541823 | 30.511 |
| GERD | TB-BMD | rs7586085 | 2 | 166577489 | G | A | -0.053 | 0.006 | 8.63575E-21 |  | G | A | -0.004 | 0.005 | 0.366 | 0.001545315 | 87.108 |
| GERD | TB-BMD | rs7728694 | 5 | 88327782 | T | G | -0.050 | 0.006 | 1.29599E-17 |  | T | G | -0.005 | 0.005 | 0.315 | 0.001289694 | 72.680 |
| GERD | TB-BMD | rs7740042 | 6 | 151971720 | A | T | -0.049 | 0.007 | 2.70707E-12 |  | A | T | 0.011 | 0.006 | 0.051 | 0.000859367 | 48.409 |
| GERD | TB-BMD | rs7741085 | 6 | 44636919 | T | C | 0.042 | 0.006 | 1.50904E-13 |  | T | C | -0.001 | 0.005 | 0.876 | 0.00097751 | 55.070 |
| GERD | TB-BMD | rs8070128 | 17 | 17804725 | T | C | -0.039 | 0.006 | 1.98381E-11 |  | T | C | 0.010 | 0.005 | 0.045 | 0.000791698 | 44.594 |
| GERD | TB-BMD | rs818427 | 5 | 112221869 | T | C | 0.034 | 0.006 | 2.37197E-08 |  | T | C | 0.010 | 0.005 | 0.061 | 0.000558168 | 31.432 |
| GERD | TB-BMD | rs838721 | 2 | 234308782 | G | A | 0.031 | 0.006 | 4.48002E-08 |  | G | A | -0.007 | 0.005 | 0.133 | 0.000538878 | 30.345 |
| GERD | TB-BMD | rs884205 | 18 | 60054857 | C | A | 0.053 | 0.007 | 4.39036E-15 |  | C | A | 0.004 | 0.006 | 0.430 | 0.001082221 | 60.976 |
| GERD | TB-BMD | rs9594738 | 13 | 42952145 | T | C | -0.061 | 0.006 | 3.84061E-27 |  | T | C | 0.002 | 0.005 | 0.667 | 0.002057348 | 116.030 |
| GERD | TB-BMD | rs9910055 | 17 | 42283037 | T | C | 0.044 | 0.007 | 3.11889E-11 |  | T | C | 0.001 | 0.006 | 0.847 | 0.000772635 | 43.519 |
| GERD | TB-BMD | rs9972944 | 17 | 63772091 | G | A | -0.036 | 0.006 | 6.86594E-10 |  | G | A | 0.006 | 0.005 | 0.259 | 0.000672098 | 37.852 |
| GERD | TB-BMD | rs9976876 | 21 | 36970350 | T | G | -0.038 | 0.006 | 8.00571E-11 |  | T | G | 0.008 | 0.005 | 0.098 | 0.000742163 | 41.801 |
| GERD | TB-BMD-1 | rs10824760 | 10 | 54439699 | T | C | 0.089 | 0.015 | 3.05401E-09 |  | T | C | -0.003 | 0.008 | 0.699 | 0.001568749 | 35.355 |
| GERD | TB-BMD-1 | rs11228240 | 11 | 68254328 | T | C | -0.085 | 0.011 | 1.11712E-13 |  | T | C | 0.010 | 0.005 | 0.073 | 0.002452764 | 55.328 |
| GERD | TB-BMD-1 | rs1936792 | 6 | 127423055 | A | G | 0.061 | 0.011 | 3.71604E-08 |  | A | G | -0.007 | 0.006 | 0.199 | 0.001351279 | 30.448 |
| GERD | TB-BMD-1 | rs2741856 | 17 | 41826839 | C | G | 0.139 | 0.019 | 7.0291E-13 |  | C | G | -0.007 | 0.009 | 0.397 | 0.002279293 | 51.406 |
| GERD | TB-BMD-1 | rs34920465 | 1 | 22706434 | G | A | 0.101 | 0.013 | 9.41022E-16 |  | G | A | -0.012 | 0.006 | 0.066 | 0.002835874 | 63.994 |
| GERD | TB-BMD-1 | rs3801387 | 7 | 120974765 | G | A | 0.134 | 0.011 | 2.81514E-35 |  | G | A | -0.014 | 0.005 | 0.012 | 0.006764069 | 153.242 |
| GERD | TB-BMD-1 | rs56104760 | 1 | 22492887 | G | A | -0.072 | 0.013 | 1.99802E-08 |  | G | A | -0.005 | 0.006 | 0.417 | 0.00140648 | 31.693 |
| GERD | TB-BMD-1 | rs61884328 | 11 | 46820788 | C | T | 0.106 | 0.017 | 2.45799E-10 |  | C | T | 0.002 | 0.008 | 0.840 | 0.001775891 | 40.032 |
| GERD | TB-BMD-1 | rs6942191 | 6 | 44623887 | A | G | -0.065 | 0.010 | 4.22902E-10 |  | A | G | 0.001 | 0.005 | 0.886 | 0.001739519 | 39.211 |
| GERD | TB-BMD-1 | rs7131442 | 11 | 16352545 | T | A | 0.076 | 0.012 | 1.80601E-10 |  | T | A | -0.006 | 0.006 | 0.275 | 0.001818719 | 40.999 |
| GERD | TB-BMD-1 | rs7548588 | 1 | 110482335 | C | T | 0.062 | 0.010 | 3.79901E-10 |  | C | T | -0.015 | 0.005 | 0.002 | 0.001723024 | 38.838 |
| GERD | TB-BMD-1 | rs7740042 | 6 | 151971720 | A | T | -0.074 | 0.012 | 9.04899E-10 |  | A | T | 0.011 | 0.006 | 0.051 | 0.00166373 | 37.500 |
| GERD | TB-BMD-1 | rs7787512 | 7 | 96141701 | T | C | 0.085 | 0.010 | 3.55795E-17 |  | T | C | -0.007 | 0.005 | 0.157 | 0.003130051 | 70.654 |
| GERD | TB-BMD-2 | rs13130558 | 4 | 88848608 | A | G | -0.070 | 0.011 | 1.47001E-10 |  | A | G | 0.002 | 0.005 | 0.655 | 0.002175898 | 41.003 |
| GERD | TB-BMD-2 | rs1968294 | 2 | 166618262 | T | C | -0.063 | 0.011 | 4.40798E-09 |  | T | C | -0.006 | 0.005 | 0.178 | 0.001817704 | 34.241 |
| GERD | TB-BMD-2 | rs3801387 | 7 | 120974765 | G | A | 0.136 | 0.012 | 3.4898E-30 |  | G | A | -0.014 | 0.005 | 0.012 | 0.006887634 | 130.406 |
| GERD | TB-BMD-2 | rs476597 | 11 | 86886175 | T | C | -0.071 | 0.012 | 8.32799E-10 |  | T | C | -0.001 | 0.005 | 0.811 | 0.002000188 | 37.685 |
| GERD | TB-BMD-2 | rs6465510 | 7 | 96148577 | A | C | 0.079 | 0.011 | 1.79308E-12 |  | A | C | -0.007 | 0.005 | 0.160 | 0.002618794 | 49.370 |
| GERD | TB-BMD-2 | rs6510186 | 19 | 31654615 | C | T | -0.068 | 0.012 | 3.11401E-08 |  | C | T | -0.001 | 0.006 | 0.897 | 0.001634834 | 30.790 |
| GERD | TB-BMD-2 | rs6679981 | 1 | 22698447 | A | G | 0.116 | 0.014 | 4.80507E-17 |  | A | G | -0.012 | 0.006 | 0.053 | 0.00375618 | 70.894 |
| GERD | TB-BMD-2 | rs6827815 | 4 | 999445 | G | C | -0.120 | 0.016 | 6.19441E-14 |  | G | C | -0.004 | 0.007 | 0.592 | 0.002982305 | 56.244 |
| GERD | TB-BMD-2 | rs6894139 | 5 | 88327782 | G | T | -0.062 | 0.011 | 9.74406E-09 |  | G | T | -0.005 | 0.005 | 0.315 | 0.001738203 | 32.740 |
| GERD | TB-BMD-2 | rs7010267 | 8 | 119957625 | A | C | 0.084 | 0.011 | 3.34195E-15 |  | A | C | -0.005 | 0.005 | 0.262 | 0.003312546 | 62.493 |
| GERD | TB-BMD-2 | rs7105218 | 11 | 68290234 | A | C | -0.109 | 0.015 | 1.78608E-13 |  | A | C | 0.019 | 0.007 | 0.004 | 0.002893963 | 54.573 |
| GERD | TB-BMD-2 | rs7398996 | 12 | 53662735 | T | C | -0.076 | 0.011 | 1.56315E-11 |  | T | C | 0.005 | 0.005 | 0.318 | 0.002412297 | 45.468 |
| GERD | TB-BMD-2 | rs7740042 | 6 | 151971720 | A | T | -0.077 | 0.013 | 6.99407E-09 |  | A | T | 0.011 | 0.006 | 0.051 | 0.001766478 | 33.274 |
| GERD | TB-BMD-2 | rs884205 | 18 | 60054857 | C | A | 0.072 | 0.013 | 9.96506E-09 |  | C | A | 0.004 | 0.006 | 0.430 | 0.001733393 | 32.650 |
| GERD | TB-BMD-2 | rs9594738 | 13 | 42952145 | T | C | -0.091 | 0.011 | 9.94947E-18 |  | T | C | 0.002 | 0.005 | 0.667 | 0.003921014 | 74.017 |
| GERD | TB-BMD-3 | rs10005067 | 4 | 88852643 | C | T | -0.088 | 0.015 | 2.16302E-09 |  | C | T | 0.002 | 0.005 | 0.612 | 0.003525169 | 35.589 |
| GERD | TB-BMD-3 | rs11228240 | 11 | 68254328 | T | C | -0.097 | 0.018 | 2.57899E-08 |  | T | C | 0.010 | 0.005 | 0.073 | 0.003069181 | 30.971 |
| GERD | TB-BMD-3 | rs12534510 | 7 | 120732120 | C | A | 0.083 | 0.015 | 2.19902E-08 |  | C | A | -0.004 | 0.005 | 0.458 | 0.003115969 | 31.445 |
| GERD | TB-BMD-3 | rs12742784 | 1 | 22682366 | T | C | 0.113 | 0.018 | 6.63896E-10 |  | T | C | -0.008 | 0.006 | 0.198 | 0.003789664 | 38.269 |
| GERD | TB-BMD-3 | rs3801387 | 7 | 120974765 | G | A | 0.162 | 0.016 | 1.42495E-23 |  | G | A | -0.014 | 0.005 | 0.012 | 0.009804527 | 99.610 |
| GERD | TB-BMD-3 | rs55781332 | 11 | 249131 | G | A | 0.109 | 0.018 | 6.98393E-10 |  | G | A | -0.002 | 0.006 | 0.685 | 0.003741099 | 37.777 |
| GERD | TB-BMD-3 | rs62259232 | 3 | 41113076 | A | G | 0.090 | 0.015 | 1.21099E-09 |  | A | G | -0.011 | 0.005 | 0.025 | 0.003653608 | 36.890 |
| GERD | TB-BMD-3 | rs7761420 | 6 | 151872567 | T | C | 0.120 | 0.015 | 7.95426E-16 |  | T | C | 0.001 | 0.005 | 0.785 | 0.006437589 | 65.182 |
| GERD | TB-BMD-4 | rs12097230 | 1 | 22685251 | A | G | 0.105 | 0.022 | 0.000002026 |  | A | G | -0.003 | 0.005 | 0.500 | 0.005353567 | 22.488 |
| GERD | TB-BMD-4 | rs2303518 | 15 | 42109975 | G | T | -0.113 | 0.024 | 2.42198E-06 |  | G | T | 0.010 | 0.005 | 0.039 | 0.005291412 | 22.225 |
| GERD | TB-BMD-4 | rs28830005 | 16 | 87194194 | A | G | -0.108 | 0.023 | 3.11602E-06 |  | A | G | 0.004 | 0.005 | 0.421 | 0.005157622 | 21.660 |
| GERD | TB-BMD-4 | rs56312530 | 11 | 68223074 | A | G | -0.172 | 0.033 | 2.601E-07 |  | A | G | 0.018 | 0.007 | 0.006 | 0.006320077 | 26.573 |
| GERD | TB-BMD-4 | rs56364616 | 7 | 121018579 | A | C | 0.139 | 0.026 | 1.426E-07 |  | A | C | -0.013 | 0.005 | 0.014 | 0.006607168 | 27.788 |
| GERD | TB-BMD-4 | rs628942 | 1 | 30879152 | G | T | -0.146 | 0.032 | 3.94203E-06 |  | G | T | 0.006 | 0.007 | 0.373 | 0.005060174 | 21.249 |
| GERD | TB-BMD-4 | rs7104977 | 11 | 121446059 | A | G | -0.110 | 0.024 | 4.20804E-06 |  | A | G | 0.012 | 0.005 | 0.020 | 0.005033058 | 21.134 |
| GERD | TB-BMD-5 | rs10896329 | 11 | 68254627 | G | A | -0.085 | 0.015 | 1.181E-08 |  | G | A | 0.010 | 0.005 | 0.073 | 0.002755173 | 32.615 |
| GERD | TB-BMD-5 | rs11934731 | 4 | 88849669 | A | G | -0.078 | 0.014 | 1.51901E-08 |  | A | G | -0.002 | 0.005 | 0.765 | 0.002716938 | 32.161 |
| GERD | TB-BMD-5 | rs35969972 | 2 | 166576956 | T | C | 0.077 | 0.013 | 2.00701E-09 |  | T | C | 0.004 | 0.005 | 0.364 | 0.003016323 | 35.715 |
| GERD | TB-BMD-5 | rs72699866 | 14 | 93115410 | A | G | -0.099 | 0.017 | 1.008E-08 |  | A | G | 0.005 | 0.006 | 0.412 | 0.002788229 | 33.007 |
| GERD | TB-BMD-5 | rs917726 | 7 | 121018857 | T | A | 0.137 | 0.015 | 5.07224E-21 |  | T | A | -0.014 | 0.005 | 0.013 | 0.007402341 | 88.036 |
| GERD | TB-BMD-5 | rs9525638 | 13 | 43128577 | C | T | 0.084 | 0.013 | 1.89802E-10 |  | C | T | 0.002 | 0.005 | 0.666 | 0.003450613 | 40.876 |
| GERD | FN-BMD | rs10170839 | 2 | 166583244 | C | A | -0.059 | 0.008 | 1.20005E-14 |  | C | A | -0.004 | 0.005 | 0.393 | 0.001244517 | 62.286 |
| GERD | FN-BMD | rs1366594 | 5 | 88376061 | C | A | -0.079 | 0.008 | 5.44001E-25 |  | C | A | -0.004 | 0.005 | 0.399 | 0.002225227 | 111.478 |
| GERD | FN-BMD | rs1485307 | 8 | 120008371 | C | T | -0.062 | 0.008 | 2.49E-15 |  | C | T | 0.006 | 0.005 | 0.201 | 0.001308926 | 65.514 |
| GERD | FN-BMD | rs1785493 | 11 | 68360887 | T | C | -0.045 | 0.008 | 4.06004E-08 |  | T | C | 0.005 | 0.005 | 0.372 | 0.000629795 | 31.501 |
| GERD | FN-BMD | rs2741856 | 17 | 41826839 | C | G | 0.088 | 0.014 | 1.33999E-09 |  | C | G | -0.007 | 0.009 | 0.397 | 0.000768723 | 38.455 |
| GERD | FN-BMD | rs3779381 | 7 | 120966790 | G | A | 0.058 | 0.009 | 2.87012E-11 |  | G | A | -0.013 | 0.005 | 0.015 | 0.000925423 | 46.301 |
| GERD | FN-BMD | rs436448 | 3 | 41121251 | C | T | 0.064 | 0.008 | 1.55991E-16 |  | C | T | -0.009 | 0.005 | 0.063 | 0.001422826 | 71.223 |
| GERD | FN-BMD | rs4448201 | 7 | 96154912 | C | G | 0.066 | 0.008 | 4.37019E-16 |  | C | G | -0.007 | 0.005 | 0.154 | 0.001380537 | 69.103 |
| GERD | FN-BMD | rs7108738 | 11 | 15710084 | G | T | 0.083 | 0.010 | 8.07049E-17 |  | G | T | -0.004 | 0.006 | 0.505 | 0.001450023 | 72.586 |
| GERD | FN-BMD | rs7524102 | 1 | 22698447 | G | A | 0.084 | 0.010 | 7.36038E-17 |  | G | A | -0.012 | 0.006 | 0.053 | 0.001453722 | 72.772 |
| GERD | FN-BMD | rs9478217 | 6 | 151895777 | G | A | 0.053 | 0.008 | 1.22999E-11 |  | G | A | 0.000 | 0.005 | 0.976 | 0.000960263 | 48.046 |
| GERD | LS-BMD | rs1023940 | 6 | 151932778 | G | A | -0.065 | 0.009 | 6.46994E-13 |  | G | A | 0.005 | 0.005 | 0.344 | 0.00121207 | 54.280 |
| GERD | LS-BMD | rs11002249 | 10 | 79447228 | T | C | 0.070 | 0.011 | 1.01E-09 |  | T | C | -0.005 | 0.006 | 0.440 | 0.000875157 | 39.179 |
| GERD | LS-BMD | rs1357651 | 7 | 38097862 | G | T | 0.068 | 0.009 | 3.74973E-13 |  | G | T | -0.006 | 0.005 | 0.251 | 0.001237165 | 55.406 |
| GERD | LS-BMD | rs2235811 | 20 | 10644158 | G | A | -0.054 | 0.009 | 4.66004E-09 |  | G | A | -0.007 | 0.005 | 0.133 | 0.000805082 | 36.040 |
| GERD | LS-BMD | rs2291467 | 11 | 68257059 | T | C | -0.077 | 0.010 | 9.64051E-14 |  | T | C | 0.009 | 0.005 | 0.077 | 0.001299521 | 58.202 |
| GERD | LS-BMD | rs6965122 | 7 | 96137674 | G | A | -0.062 | 0.009 | 7.39946E-11 |  | G | A | 0.007 | 0.005 | 0.158 | 0.000994588 | 44.531 |
| GERD | LS-BMD | rs73326583 | 14 | 91466507 | A | C | 0.072 | 0.012 | 2.83002E-09 |  | A | C | -0.002 | 0.006 | 0.742 | 0.00082779 | 37.057 |
| GERD | LS-BMD | rs7524102 | 1 | 22698447 | G | A | 0.090 | 0.011 | 2.40991E-14 |  | G | A | -0.012 | 0.006 | 0.053 | 0.001363396 | 61.067 |
| GERD | LS-BMD | rs7807953 | 7 | 121018579 | T | C | 0.075 | 0.010 | 4.1096E-14 |  | T | C | -0.013 | 0.005 | 0.014 | 0.0013389 | 59.968 |
| GERD | LS-BMD | rs884205 | 18 | 60054857 | C | A | 0.062 | 0.010 | 2.77E-09 |  | C | A | 0.004 | 0.006 | 0.430 | 0.000828793 | 37.102 |
| GERD | LS-BMD | rs894738 | 12 | 54418920 | A | G | -0.063 | 0.009 | 1.99986E-11 |  | A | G | 0.013 | 0.005 | 0.012 | 0.001054642 | 47.223 |
| GERD | LS-BMD | rs9749364 | 19 | 33527596 | G | T | 0.114 | 0.018 | 6.64003E-10 |  | G | T | 0.012 | 0.009 | 0.172 | 0.000894133 | 40.029 |
| GERD | LS-BMD | rs9921222 | 16 | 375782 | T | C | -0.053 | 0.009 | 3.16002E-09 |  | T | C | 0.009 | 0.005 | 0.062 | 0.000822891 | 36.837 |
| GERD | H-BMD | rs10015974 | 4 | 184212220 | G | A | -0.015 | 0.002 | 5.49997E-09 |  | G | A | -0.007 | 0.006 | 0.186 | 0.000109724 | 46.838 |
| GERD | H-BMD | rs10087008 | 8 | 89610885 | A | T | -0.014 | 0.002 | 9.90011E-10 |  | A | T | -0.004 | 0.006 | 0.490 | 0.000103288 | 44.090 |
| GERD | H-BMD | rs10145299 | 14 | 35224294 | C | T | 0.022 | 0.002 | 7.59976E-27 |  | C | T | -0.012 | 0.005 | 0.012 | 0.000329847 | 140.833 |
| GERD | H-BMD | rs10148621 | 14 | 89894880 | A | T | 0.015 | 0.003 | 6.80002E-09 |  | A | T | 0.006 | 0.007 | 0.361 | 8.61223E-05 | 36.762 |
| GERD | H-BMD | rs10206992 | 2 | 183730527 | G | T | -0.019 | 0.002 | 5.30029E-17 |  | G | T | 0.009 | 0.006 | 0.114 | 0.000182942 | 78.098 |
| GERD | H-BMD | rs10222078 | 21 | 37001464 | A | G | -0.019 | 0.002 | 1.29987E-16 |  | A | G | 0.008 | 0.005 | 0.132 | 0.000241408 | 103.063 |
| GERD | H-BMD | rs10245919 | 7 | 19549650 | T | C | -0.013 | 0.002 | 9.09913E-11 |  | T | C | 0.012 | 0.005 | 0.018 | 0.000110406 | 47.129 |
| GERD | H-BMD | rs10264106 | 7 | 37947818 | C | A | 0.049 | 0.002 | 1.50003E-72 |  | C | A | 0.000 | 0.006 | 0.941 | 0.001013779 | 433.142 |
| GERD | H-BMD | rs10407062 | 19 | 57822369 | C | T | -0.014 | 0.002 | 2.60016E-11 |  | C | T | -0.001 | 0.005 | 0.819 | 0.00012706 | 54.239 |
| GERD | H-BMD | rs10410950 | 19 | 13128861 | A | G | -0.032 | 0.004 | 6.59933E-11 |  | A | G | -0.004 | 0.011 | 0.743 | 0.000130601 | 55.751 |
| GERD | H-BMD | rs1042704 | 14 | 23312594 | A | G | -0.028 | 0.002 | 5.19996E-28 |  | A | G | -0.003 | 0.006 | 0.570 | 0.000351451 | 150.060 |
| GERD | H-BMD | rs1043003 | 10 | 3820787 | C | T | -0.017 | 0.002 | 3.59998E-12 |  | C | T | 0.002 | 0.005 | 0.741 | 0.000189686 | 80.978 |
| GERD | H-BMD | rs10474093 | 5 | 82447802 | C | T | -0.011 | 0.002 | 0.000000012 |  | C | T | 0.000 | 0.005 | 0.990 | 8.67835E-05 | 37.044 |
| GERD | H-BMD | rs10490046 | 2 | 40630678 | C | A | -0.029 | 0.002 | 7.00003E-30 |  | C | A | -0.002 | 0.006 | 0.686 | 0.00038742 | 165.424 |
| GERD | H-BMD | rs10739971 | 9 | 96937680 | A | G | 0.020 | 0.002 | 2.29985E-21 |  | A | G | 0.013 | 0.005 | 0.011 | 0.000226292 | 96.608 |
| GERD | H-BMD | rs10740042 | 10 | 62496586 | C | T | 0.016 | 0.002 | 3.80014E-12 |  | C | T | 0.003 | 0.005 | 0.502 | 0.000144054 | 61.494 |
| GERD | H-BMD | rs10764201 | 10 | 20379378 | T | C | 0.020 | 0.002 | 4.60045E-19 |  | T | C | 0.002 | 0.005 | 0.639 | 0.000280517 | 119.764 |
| GERD | H-BMD | rs10765568 | 11 | 92623515 | T | C | -0.017 | 0.002 | 2.70023E-15 |  | T | C | 0.002 | 0.005 | 0.631 | 0.000195237 | 83.348 |
| GERD | H-BMD | rs10779795 | 1 | 6677064 | G | A | -0.020 | 0.002 | 4.40048E-22 |  | G | A | -0.006 | 0.005 | 0.251 | 0.000243119 | 103.794 |
| GERD | H-BMD | rs10783573 | 12 | 53742885 | A | G | -0.016 | 0.002 | 5.40008E-11 |  | A | G | 0.001 | 0.005 | 0.819 | 0.000159062 | 67.902 |
| GERD | H-BMD | rs10788221 | 10 | 123658228 | A | G | 0.017 | 0.002 | 2.19989E-16 |  | A | G | 0.001 | 0.005 | 0.778 | 0.000171784 | 73.334 |
| GERD | H-BMD | rs10817895 | 9 | 119232655 | T | A | 0.016 | 0.002 | 3.2E-10 |  | T | A | -0.004 | 0.005 | 0.486 | 0.000130993 | 55.918 |
| GERD | H-BMD | rs10824766 | 10 | 54431210 | T | C | -0.129 | 0.003 | 1E-200 |  | T | C | 0.003 | 0.008 | 0.663 | 0.004360038 | 1869.109 |
| GERD | H-BMD | rs10842704 | 12 | 26465585 | G | T | 0.028 | 0.002 | 3.40017E-28 |  | G | T | 0.002 | 0.006 | 0.781 | 0.000388082 | 165.706 |
| GERD | H-BMD | rs10887745 | 10 | 89481871 | T | G | -0.016 | 0.002 | 1.99986E-14 |  | T | G | -0.003 | 0.005 | 0.510 | 0.000171145 | 73.061 |
| GERD | H-BMD | rs10896426 | 11 | 68869923 | C | T | 0.025 | 0.002 | 1.39991E-33 |  | C | T | 0.005 | 0.005 | 0.319 | 0.000433076 | 184.927 |
| GERD | H-BMD | rs10917449 | 1 | 19770256 | G | A | -0.019 | 0.002 | 8.69961E-17 |  | G | A | -0.003 | 0.005 | 0.627 | 0.000190504 | 81.327 |
| GERD | H-BMD | rs10920352 | 1 | 202148042 | C | T | 0.011 | 0.002 | 4E-10 |  | C | T | -0.004 | 0.005 | 0.423 | 8.91533E-05 | 38.056 |
| GERD | H-BMD | rs10931982 | 2 | 202832130 | C | T | 0.054 | 0.002 | 2.9992E-107 |  | C | T | -0.001 | 0.006 | 0.878 | 0.001418362 | 606.248 |
| GERD | H-BMD | rs11007241 | 10 | 29075122 | A | G | -0.016 | 0.002 | 8.60003E-12 |  | A | G | -0.011 | 0.006 | 0.050 | 0.000130449 | 55.686 |
| GERD | H-BMD | rs11023882 | 11 | 16253569 | A | G | -0.013 | 0.002 | 3.2E-09 |  | A | G | 0.003 | 0.005 | 0.511 | 0.000111373 | 47.542 |
| GERD | H-BMD | rs11067228 | 12 | 115094260 | G | A | 0.016 | 0.002 | 2.09991E-16 |  | G | A | 0.004 | 0.005 | 0.393 | 0.000167107 | 71.337 |
| GERD | H-BMD | rs11107263 | 12 | 94305374 | A | C | 0.018 | 0.003 | 1.7E-09 |  | A | C | 0.002 | 0.007 | 0.752 | 9.42402E-05 | 40.228 |
| GERD | H-BMD | rs11127656 | 3 | 79739700 | A | G | -0.012 | 0.002 | 2.80001E-10 |  | A | G | -0.007 | 0.005 | 0.192 | 7.76649E-05 | 33.152 |
| GERD | H-BMD | rs11175773 | 12 | 65861817 | T | C | 0.022 | 0.003 | 4.20001E-08 |  | T | C | -0.002 | 0.008 | 0.770 | 0.000107839 | 46.033 |
| GERD | H-BMD | rs11175835 | 12 | 65995687 | A | G | -0.023 | 0.002 | 2.09991E-22 |  | A | G | -0.005 | 0.005 | 0.329 | 0.00029485 | 125.886 |
| GERD | H-BMD | rs11196170 | 10 | 114726177 | A | G | -0.030 | 0.002 | 2.90001E-31 |  | A | G | 0.000 | 0.006 | 0.975 | 0.000405529 | 173.159 |
| GERD | H-BMD | rs11228240 | 11 | 68254328 | T | C | -0.043 | 0.002 | 8.00018E-77 |  | T | C | 0.010 | 0.005 | 0.073 | 0.001007337 | 430.387 |
| GERD | H-BMD | rs11238756 | 10 | 44370872 | C | T | 0.012 | 0.002 | 5.49997E-10 |  | C | T | -0.005 | 0.005 | 0.312 | 0.0001038 | 44.309 |
| GERD | H-BMD | rs11242776 | 6 | 2445280 | C | T | -0.014 | 0.002 | 3.79997E-09 |  | C | T | -0.002 | 0.005 | 0.754 | 0.000136143 | 58.117 |
| GERD | H-BMD | rs1133400 | 10 | 134459388 | G | A | 0.031 | 0.002 | 4.60045E-34 |  | G | A | 0.000 | 0.006 | 0.974 | 0.000445031 | 190.033 |
| GERD | H-BMD | rs113488960 | 7 | 119764382 | C | G | 0.055 | 0.008 | 3.59998E-11 |  | C | G | -0.031 | 0.018 | 0.083 | 0.0001272 | 54.298 |
| GERD | H-BMD | rs11576308 | 1 | 103379918 | A | G | 0.015 | 0.002 | 2.70023E-15 |  | A | G | 0.014 | 0.005 | 0.003 | 0.000155733 | 66.480 |
| GERD | H-BMD | rs1159798 | 10 | 54412493 | C | A | -0.064 | 0.002 | 6.6989E-154 |  | C | A | -0.003 | 0.006 | 0.657 | 0.001965123 | 840.409 |
| GERD | H-BMD | rs11643240 | 16 | 69635916 | G | A | -0.015 | 0.002 | 2.09991E-12 |  | G | A | 0.013 | 0.005 | 0.013 | 0.000125541 | 53.591 |
| GERD | H-BMD | rs11675489 | 2 | 85498783 | G | A | -0.013 | 0.002 | 1.29999E-09 |  | G | A | -0.001 | 0.005 | 0.837 | 0.000118934 | 50.770 |
| GERD | H-BMD | rs11696009 | 2 | 72330334 | C | A | 0.016 | 0.002 | 2.29985E-14 |  | C | A | -0.007 | 0.005 | 0.180 | 0.000154127 | 65.795 |
| GERD | H-BMD | rs11729023 | 4 | 71992629 | T | C | 0.025 | 0.003 | 1.29987E-14 |  | T | C | 0.002 | 0.007 | 0.765 | 0.000182247 | 77.801 |
| GERD | H-BMD | rs11752873 | 6 | 155363822 | T | C | 0.019 | 0.003 | 1.29999E-09 |  | T | C | 0.007 | 0.007 | 0.329 | 0.000108106 | 46.147 |
| GERD | H-BMD | rs1181334 | 12 | 12367588 | G | T | -0.023 | 0.002 | 5.40008E-20 |  | G | T | 0.003 | 0.006 | 0.664 | 0.000217045 | 92.660 |
| GERD | H-BMD | rs1187624 | 14 | 95666029 | C | A | -0.024 | 0.004 | 2.19999E-08 |  | C | A | 0.009 | 0.010 | 0.360 | 9.26299E-05 | 39.540 |
| GERD | H-BMD | rs11880992 | 19 | 2176403 | A | G | -0.017 | 0.002 | 2.19989E-15 |  | A | G | 0.003 | 0.005 | 0.607 | 0.000183041 | 78.140 |
| GERD | H-BMD | rs11915970 | 3 | 56169965 | T | A | 0.044 | 0.003 | 9.09913E-46 |  | T | A | 0.014 | 0.007 | 0.053 | 0.000555839 | 237.376 |
| GERD | H-BMD | rs11934731 | 4 | 88849669 | A | G | -0.036 | 0.002 | 3.40017E-53 |  | A | G | -0.002 | 0.005 | 0.765 | 0.000762717 | 325.793 |
| GERD | H-BMD | rs11995050 | 8 | 25310042 | C | A | 0.015 | 0.002 | 2.39994E-13 |  | C | A | -0.005 | 0.006 | 0.402 | 0.000101587 | 43.364 |
| GERD | H-BMD | rs12041821 | 1 | 41969059 | A | C | -0.014 | 0.002 | 0.000000032 |  | A | C | 0.008 | 0.006 | 0.209 | 8.21622E-05 | 35.072 |
| GERD | H-BMD | rs12121554 | 1 | 220031791 | A | G | 0.024 | 0.002 | 3.80014E-26 |  | A | G | 0.002 | 0.005 | 0.691 | 0.000334187 | 142.686 |
| GERD | H-BMD | rs12127020 | 1 | 68404391 | G | C | 0.017 | 0.002 | 7.29962E-12 |  | G | C | 0.004 | 0.006 | 0.523 | 0.000127395 | 54.382 |
| GERD | H-BMD | rs12149673 | 16 | 55069361 | G | A | 0.029 | 0.002 | 1.29987E-38 |  | G | A | -0.006 | 0.005 | 0.239 | 0.000475241 | 202.940 |
| GERD | H-BMD | rs12210993 | 6 | 2481290 | A | G | -0.022 | 0.002 | 3.59998E-26 |  | A | G | -0.016 | 0.005 | 0.001 | 0.000346259 | 147.842 |
| GERD | H-BMD | rs12215938 | 6 | 155329941 | T | C | 0.024 | 0.003 | 3.69999E-18 |  | T | C | 0.004 | 0.007 | 0.582 | 0.00019545 | 83.439 |
| GERD | H-BMD | rs12276167 | 11 | 27635571 | G | T | -0.014 | 0.002 | 4.10015E-12 |  | G | T | 0.009 | 0.005 | 0.061 | 0.00012137 | 51.810 |
| GERD | H-BMD | rs12372718 | 12 | 51171090 | G | A | -0.015 | 0.002 | 2.60016E-13 |  | G | A | 0.003 | 0.005 | 0.563 | 0.000145889 | 62.278 |
| GERD | H-BMD | rs12443188 | 15 | 67433941 | A | T | 0.022 | 0.002 | 1.29987E-18 |  | A | T | -0.005 | 0.006 | 0.386 | 0.000239541 | 102.266 |
| GERD | H-BMD | rs12448684 | 16 | 81583418 | A | G | -0.024 | 0.002 | 1.39991E-31 |  | A | G | 0.008 | 0.005 | 0.087 | 0.000392594 | 167.634 |
| GERD | H-BMD | rs12485244 | 22 | 29429369 | A | G | 0.017 | 0.002 | 5.50047E-15 |  | A | G | 0.000 | 0.005 | 0.928 | 0.000190479 | 81.316 |
| GERD | H-BMD | rs12487905 | 3 | 124521035 | C | T | 0.013 | 0.002 | 4.79999E-08 |  | C | T | -0.007 | 0.006 | 0.210 | 7.34751E-05 | 31.363 |
| GERD | H-BMD | rs12499988 | 4 | 146781251 | T | C | -0.016 | 0.002 | 1.59993E-13 |  | T | C | -0.005 | 0.005 | 0.280 | 0.000169199 | 72.230 |
| GERD | H-BMD | rs12545602 | 8 | 100846840 | A | C | 0.021 | 0.003 | 8.4004E-13 |  | A | C | 0.005 | 0.007 | 0.448 | 0.000136732 | 58.368 |
| GERD | H-BMD | rs1256065 | 14 | 64698932 | T | G | 0.012 | 0.002 | 0.000000015 |  | T | G | 0.001 | 0.005 | 0.831 | 9.84893E-05 | 42.042 |
| GERD | H-BMD | rs12569559 | 10 | 60314275 | G | T | 0.019 | 0.002 | 4.00037E-18 |  | G | T | 0.007 | 0.005 | 0.125 | 0.00023633 | 100.895 |
| GERD | H-BMD | rs12616772 | 2 | 177531503 | C | A | -0.011 | 0.002 | 5.49997E-09 |  | C | A | -0.005 | 0.005 | 0.297 | 8.36162E-05 | 35.692 |
| GERD | H-BMD | rs12733821 | 1 | 162086691 | C | G | 0.016 | 0.002 | 5.50047E-16 |  | C | G | -0.002 | 0.005 | 0.691 | 0.000165679 | 70.727 |
| GERD | H-BMD | rs12756373 | 1 | 85333182 | C | A | 0.021 | 0.003 | 1.79999E-10 |  | C | A | -0.004 | 0.009 | 0.649 | 9.54667E-05 | 40.751 |
| GERD | H-BMD | rs12811685 | 12 | 78222363 | G | C | -0.018 | 0.002 | 1.69981E-14 |  | G | C | 0.017 | 0.005 | 0.001 | 0.000192657 | 82.246 |
| GERD | H-BMD | rs1286075 | 14 | 91452841 | T | C | 0.031 | 0.002 | 2.99985E-30 |  | T | C | -0.002 | 0.006 | 0.757 | 0.000359165 | 153.355 |
| GERD | H-BMD | rs1286662 | 3 | 25529280 | G | A | -0.022 | 0.002 | 8.19974E-16 |  | G | A | 0.003 | 0.006 | 0.650 | 0.000205639 | 87.789 |
| GERD | H-BMD | rs12924535 | 16 | 51829902 | T | G | 0.022 | 0.002 | 1.29987E-25 |  | T | G | 0.000 | 0.005 | 0.923 | 0.00028871 | 123.263 |
| GERD | H-BMD | rs12941363 | 17 | 4069735 | A | C | 0.013 | 0.002 | 4.70002E-08 |  | A | C | -0.005 | 0.006 | 0.457 | 6.66813E-05 | 28.463 |
| GERD | H-BMD | rs12942736 | 17 | 69862975 | T | C | -0.024 | 0.002 | 3.80014E-23 |  | T | C | -0.003 | 0.006 | 0.582 | 0.00027043 | 115.457 |
| GERD | H-BMD | rs12986939 | 2 | 119089007 | A | G | -0.050 | 0.002 | 3.3963E-111 |  | A | G | -0.001 | 0.005 | 0.805 | 0.001397484 | 597.312 |
| GERD | H-BMD | rs13002567 | 2 | 25482970 | C | T | 0.018 | 0.002 | 5.60015E-17 |  | C | T | -0.006 | 0.005 | 0.248 | 0.000202339 | 86.380 |
| GERD | H-BMD | rs13022378 | 2 | 202954511 | T | C | 0.023 | 0.002 | 3.19963E-29 |  | T | C | 0.007 | 0.005 | 0.167 | 0.000339394 | 144.910 |
| GERD | H-BMD | rs13070996 | 3 | 196917572 | T | G | -0.014 | 0.002 | 9.69996E-10 |  | T | G | 0.009 | 0.005 | 0.109 | 0.000113042 | 48.254 |
| GERD | H-BMD | rs13072536 | 3 | 52861211 | T | A | 0.017 | 0.002 | 2.19999E-09 |  | T | A | 0.004 | 0.006 | 0.511 | 0.000122052 | 52.101 |
| GERD | H-BMD | rs13088318 | 3 | 101242751 | G | A | 0.015 | 0.002 | 9.20026E-12 |  | G | A | -0.003 | 0.005 | 0.545 | 0.000143051 | 61.066 |
| GERD | H-BMD | rs13133616 | 4 | 157547351 | G | A | 0.019 | 0.002 | 2.70023E-13 |  | G | A | -0.005 | 0.005 | 0.378 | 0.000186637 | 79.676 |
| GERD | H-BMD | rs13154707 | 5 | 42547255 | G | A | 0.021 | 0.002 | 3.90032E-21 |  | G | A | -0.004 | 0.006 | 0.467 | 0.000224246 | 95.735 |
| GERD | H-BMD | rs13179493 | 5 | 39426307 | C | T | 0.030 | 0.002 | 4.60045E-39 |  | C | T | 0.001 | 0.005 | 0.910 | 0.000483419 | 206.434 |
| GERD | H-BMD | rs134613 | 22 | 29480905 | G | T | -0.043 | 0.002 | 3.50026E-90 |  | G | T | 0.001 | 0.005 | 0.899 | 0.001127343 | 481.718 |
| GERD | H-BMD | rs1351623 | 4 | 104587977 | T | C | -0.016 | 0.002 | 4.79999E-09 |  | T | C | 0.000 | 0.006 | 0.979 | 9.73321E-05 | 41.548 |
| GERD | H-BMD | rs1386625 | 4 | 38361120 | G | A | -0.046 | 0.003 | 2.39994E-40 |  | G | A | 0.000 | 0.008 | 0.967 | 0.000492709 | 210.403 |
| GERD | H-BMD | rs1392798 | 3 | 157056287 | A | G | -0.015 | 0.002 | 6.79986E-13 |  | A | G | 0.011 | 0.005 | 0.028 | 0.000156975 | 67.011 |
| GERD | H-BMD | rs1414660 | 1 | 240586695 | T | C | 0.081 | 0.002 | 1E-200 |  | T | C | -0.009 | 0.006 | 0.158 | 0.002862816 | 1225.421 |
| GERD | H-BMD | rs1428968 | 5 | 36646946 | T | C | 0.023 | 0.002 | 7.19946E-19 |  | T | C | 0.007 | 0.006 | 0.243 | 0.00022248 | 94.981 |
| GERD | H-BMD | rs1470580 | 3 | 185529174 | A | T | 0.015 | 0.002 | 3.40001E-08 |  | A | T | 0.010 | 0.005 | 0.060 | 0.000127835 | 54.570 |
| GERD | H-BMD | rs1475120 | 6 | 105400837 | A | G | 0.013 | 0.002 | 1.6E-10 |  | A | G | 0.017 | 0.005 | 0.000 | 0.000124479 | 53.137 |
| GERD | H-BMD | rs1487241 | 8 | 128021488 | T | A | -0.025 | 0.002 | 2.90001E-28 |  | T | A | 0.002 | 0.005 | 0.694 | 0.000372674 | 159.125 |
| GERD | H-BMD | rs1491814 | 11 | 29149173 | T | A | -0.017 | 0.003 | 3.40001E-09 |  | T | A | -0.004 | 0.007 | 0.555 | 9.40475E-05 | 40.145 |
| GERD | H-BMD | rs1502201 | 6 | 55265497 | A | G | -0.017 | 0.002 | 1.99986E-16 |  | A | G | -0.002 | 0.006 | 0.768 | 0.000157305 | 67.152 |
| GERD | H-BMD | rs1521908 | 6 | 109613986 | C | T | -0.011 | 0.002 | 3.50002E-08 |  | C | T | -0.004 | 0.005 | 0.387 | 9.19005E-05 | 39.229 |
| GERD | H-BMD | rs1528602 | 16 | 50999201 | C | T | -0.017 | 0.002 | 3.10027E-13 |  | C | T | -0.009 | 0.005 | 0.105 | 0.000153182 | 65.391 |
| GERD | H-BMD | rs1533142 | 12 | 28614139 | G | C | -0.023 | 0.002 | 2.09991E-19 |  | G | C | -0.002 | 0.006 | 0.745 | 0.000263002 | 112.285 |
| GERD | H-BMD | rs1545920 | 3 | 56286046 | T | A | 0.016 | 0.002 | 1.09999E-10 |  | T | A | -0.018 | 0.006 | 0.004 | 0.000104019 | 44.402 |
| GERD | H-BMD | rs1548607 | 7 | 50901491 | G | A | -0.018 | 0.002 | 1.10002E-14 |  | G | A | 0.006 | 0.005 | 0.238 | 0.000202661 | 86.518 |
| GERD | H-BMD | rs1550270 | 4 | 166261800 | C | T | 0.016 | 0.002 | 7.29962E-15 |  | C | T | 0.003 | 0.005 | 0.600 | 0.000149828 | 63.959 |
| GERD | H-BMD | rs1555958 | 20 | 10976046 | G | C | 0.031 | 0.002 | 6.4998E-45 |  | G | C | -0.005 | 0.005 | 0.342 | 0.000549277 | 234.572 |
| GERD | H-BMD | rs159408 | 6 | 133702841 | T | A | -0.015 | 0.002 | 6.89922E-12 |  | T | A | -0.004 | 0.005 | 0.370 | 0.000144277 | 61.590 |
| GERD | H-BMD | rs1622638 | 11 | 121816530 | A | G | 0.015 | 0.002 | 6.59933E-16 |  | A | G | 0.011 | 0.005 | 0.029 | 0.000156472 | 66.796 |
| GERD | H-BMD | rs167365 | 1 | 89234909 | G | C | -0.019 | 0.002 | 1.9002E-15 |  | G | C | -0.001 | 0.005 | 0.809 | 0.000233466 | 99.672 |
| GERD | H-BMD | rs16878921 | 5 | 50778798 | A | G | 0.034 | 0.003 | 3.80014E-26 |  | A | G | 0.014 | 0.008 | 0.077 | 0.000275654 | 117.688 |
| GERD | H-BMD | rs17010957 | 4 | 86719165 | C | T | 0.033 | 0.003 | 1.10002E-28 |  | C | T | 0.007 | 0.007 | 0.277 | 0.000366706 | 156.576 |
| GERD | H-BMD | rs1708627 | 17 | 17146959 | G | A | -0.014 | 0.002 | 3.10027E-11 |  | G | A | 0.003 | 0.005 | 0.509 | 0.000127889 | 54.593 |
| GERD | H-BMD | rs17265513 | 20 | 39832628 | C | T | -0.033 | 0.002 | 4.70002E-36 |  | C | T | -0.003 | 0.006 | 0.676 | 0.000483396 | 206.424 |
| GERD | H-BMD | rs17472350 | 7 | 27231045 | C | T | -0.074 | 0.006 | 2.09991E-27 |  | C | T | -0.009 | 0.016 | 0.577 | 0.000333432 | 142.364 |
| GERD | H-BMD | rs17507577 | 11 | 15243059 | A | G | 0.060 | 0.004 | 1E-54 |  | A | G | -0.007 | 0.009 | 0.471 | 0.000677323 | 289.292 |
| GERD | H-BMD | rs1766790 | 1 | 119459929 | T | C | -0.026 | 0.002 | 2.70023E-22 |  | T | C | 0.014 | 0.006 | 0.026 | 0.000264672 | 112.998 |
| GERD | H-BMD | rs17679410 | 8 | 96700543 | G | A | 0.025 | 0.002 | 1.80011E-27 |  | G | A | -0.001 | 0.006 | 0.878 | 0.000299992 | 128.082 |
| GERD | H-BMD | rs1771982 | 10 | 45248484 | C | T | -0.009 | 0.002 | 3.59998E-08 |  | C | T | 0.004 | 0.005 | 0.410 | 6.06273E-05 | 25.879 |
| GERD | H-BMD | rs1777277 | 1 | 56996347 | A | C | -0.011 | 0.002 | 7.49998E-09 |  | A | C | 0.012 | 0.005 | 0.015 | 8.10485E-05 | 34.596 |
| GERD | H-BMD | rs181367 | 17 | 26579562 | G | C | 0.012 | 0.002 | 3.79997E-08 |  | G | C | 0.000 | 0.005 | 0.995 | 8.00141E-05 | 34.155 |
| GERD | H-BMD | rs1834554 | 11 | 60007573 | C | G | -0.022 | 0.002 | 2.39994E-16 |  | C | G | 0.002 | 0.006 | 0.731 | 0.000192803 | 82.309 |
| GERD | H-BMD | rs1844776 | 9 | 109470527 | C | T | 0.012 | 0.002 | 4.09996E-09 |  | C | T | -0.006 | 0.005 | 0.186 | 9.72792E-05 | 41.525 |
| GERD | H-BMD | rs1891002 | 6 | 151900047 | A | T | -0.084 | 0.002 | 1E-200 |  | A | T | -0.001 | 0.005 | 0.816 | 0.004012644 | 1719.585 |
| GERD | H-BMD | rs1897468 | 2 | 237690644 | T | C | -0.013 | 0.002 | 5.60003E-10 |  | T | C | -0.002 | 0.005 | 0.660 | 9.44134E-05 | 40.302 |
| GERD | H-BMD | rs1902177 | 4 | 15099037 | T | C | 0.015 | 0.002 | 7.90005E-10 |  | T | C | -0.014 | 0.005 | 0.004 | 0.000143478 | 61.248 |
| GERD | H-BMD | rs1907310 | 10 | 78299227 | A | G | -0.021 | 0.002 | 9.3994E-15 |  | A | G | 0.002 | 0.006 | 0.745 | 0.000167619 | 71.555 |
| GERD | H-BMD | rs1977576 | 6 | 131311108 | C | T | 0.023 | 0.002 | 1.20005E-24 |  | C | T | -0.002 | 0.005 | 0.704 | 0.000354524 | 151.372 |
| GERD | H-BMD | rs1991431 | 3 | 141133450 | A | G | -0.018 | 0.002 | 2.39994E-16 |  | A | G | -0.010 | 0.005 | 0.034 | 0.000215421 | 91.966 |
| GERD | H-BMD | rs2052480 | 5 | 80282962 | A | G | 0.027 | 0.002 | 3.59998E-31 |  | A | G | -0.010 | 0.005 | 0.070 | 0.000406274 | 173.477 |
| GERD | H-BMD | rs206432 | 18 | 10356076 | T | G | 0.016 | 0.002 | 1E-13 |  | T | G | 0.000 | 0.005 | 0.937 | 0.000171591 | 73.251 |
| GERD | H-BMD | rs2069442 | 7 | 150755839 | C | G | -0.019 | 0.002 | 1.59993E-16 |  | C | G | 0.003 | 0.006 | 0.571 | 0.000200604 | 85.639 |
| GERD | H-BMD | rs2073381 | 21 | 47888763 | C | G | 0.012 | 0.002 | 0.000000005 |  | C | G | -0.003 | 0.005 | 0.543 | 8.47456E-05 | 36.174 |
| GERD | H-BMD | rs2073489 | 1 | 227063671 | C | T | -0.012 | 0.002 | 3.2E-09 |  | C | T | 0.000 | 0.005 | 0.982 | 0.000104603 | 44.652 |
| GERD | H-BMD | rs2085490 | 10 | 82201651 | G | A | 0.013 | 0.002 | 6.4998E-11 |  | G | A | -0.006 | 0.005 | 0.198 | 0.000114666 | 48.948 |
| GERD | H-BMD | rs210374 | 14 | 54155963 | T | A | 0.014 | 0.002 | 2.30001E-09 |  | T | A | -0.004 | 0.005 | 0.467 | 0.000100252 | 42.794 |
| GERD | H-BMD | rs2109204 | 12 | 3229093 | G | C | -0.013 | 0.002 | 3.29997E-09 |  | G | C | 0.004 | 0.005 | 0.493 | 8.59533E-05 | 36.690 |
| GERD | H-BMD | rs212417 | 7 | 105494963 | A | G | -0.029 | 0.002 | 4.49987E-42 |  | A | G | 0.003 | 0.005 | 0.504 | 0.000525756 | 224.522 |
| GERD | H-BMD | rs215226 | 12 | 591300 | G | A | 0.024 | 0.002 | 4.00037E-32 |  | G | A | -0.011 | 0.005 | 0.028 | 0.000382854 | 163.473 |
| GERD | H-BMD | rs2216949 | 2 | 218092221 | T | C | 0.029 | 0.003 | 1.39991E-19 |  | T | C | 0.001 | 0.007 | 0.853 | 0.000246321 | 105.161 |
| GERD | H-BMD | rs2227607 | 1 | 173880752 | A | C | -0.021 | 0.003 | 1.6E-09 |  | A | C | -0.024 | 0.008 | 0.002 | 0.000115953 | 49.497 |
| GERD | H-BMD | rs2239626 | 3 | 185998884 | C | T | 0.017 | 0.002 | 1.29999E-10 |  | C | T | -0.003 | 0.005 | 0.509 | 0.000158658 | 67.729 |
| GERD | H-BMD | rs2240226 | 17 | 42266218 | T | C | 0.034 | 0.002 | 4.60045E-47 |  | T | C | 0.004 | 0.006 | 0.449 | 0.000619995 | 264.792 |
| GERD | H-BMD | rs2346204 | 2 | 153332159 | A | C | -0.015 | 0.002 | 2.70023E-11 |  | A | C | 0.002 | 0.006 | 0.732 | 0.000114591 | 48.915 |
| GERD | H-BMD | rs2382801 | 9 | 16527129 | A | C | 0.020 | 0.002 | 1E-20 |  | A | C | -0.001 | 0.005 | 0.786 | 0.0002684 | 114.590 |
| GERD | H-BMD | rs2388792 | 10 | 3629849 | G | A | 0.013 | 0.002 | 8.10028E-11 |  | G | A | -0.005 | 0.005 | 0.310 | 0.000117622 | 50.209 |
| GERD | H-BMD | rs2442599 | 8 | 6394151 | A | G | 0.015 | 0.002 | 1.2E-10 |  | A | G | 0.006 | 0.005 | 0.280 | 0.000125788 | 53.696 |
| GERD | H-BMD | rs2446415 | 15 | 51651150 | G | A | -0.037 | 0.006 | 1.09999E-09 |  | G | A | -0.037 | 0.015 | 0.011 | 9.90199E-05 | 42.268 |
| GERD | H-BMD | rs2509353 | 11 | 115493947 | T | C | -0.021 | 0.002 | 9.79941E-22 |  | T | C | 0.001 | 0.005 | 0.758 | 0.000295582 | 126.198 |
| GERD | H-BMD | rs2546985 | 5 | 159592857 | G | A | -0.015 | 0.002 | 2.99999E-08 |  | G | A | 0.003 | 0.006 | 0.553 | 0.000100561 | 42.926 |
| GERD | H-BMD | rs2553772 | 11 | 35085453 | G | T | 0.028 | 0.002 | 5.19996E-44 |  | G | T | -0.006 | 0.005 | 0.220 | 0.000545004 | 232.747 |
| GERD | H-BMD | rs2566774 | 1 | 68694877 | C | T | -0.038 | 0.002 | 1.29987E-45 |  | C | T | 0.010 | 0.006 | 0.108 | 0.000617863 | 263.880 |
| GERD | H-BMD | rs2627668 | 4 | 88706293 | T | G | 0.025 | 0.003 | 1.59993E-13 |  | T | G | -0.011 | 0.008 | 0.149 | 0.000156372 | 66.754 |
| GERD | H-BMD | rs2637318 | 10 | 27996863 | A | G | 0.025 | 0.003 | 5.00035E-12 |  | A | G | -0.002 | 0.009 | 0.783 | 0.000124727 | 53.243 |
| GERD | H-BMD | rs2639953 | 8 | 71925660 | A | G | -0.028 | 0.002 | 3.69999E-44 |  | A | G | 0.003 | 0.005 | 0.503 | 0.000549232 | 234.553 |
| GERD | H-BMD | rs2647462 | 1 | 220156090 | C | T | -0.033 | 0.002 | 4.60045E-38 |  | C | T | -0.005 | 0.006 | 0.419 | 0.000446539 | 190.678 |
| GERD | H-BMD | rs2653559 | 11 | 8900394 | T | C | -0.029 | 0.002 | 2.49977E-26 |  | T | C | 0.013 | 0.006 | 0.050 | 0.00030986 | 132.296 |
| GERD | H-BMD | rs2722299 | 7 | 38013798 | T | C | -0.022 | 0.002 | 7.59976E-29 |  | T | C | 0.003 | 0.005 | 0.605 | 0.000330278 | 141.016 |
| GERD | H-BMD | rs2736911 | 10 | 124214355 | T | C | 0.018 | 0.003 | 4.70002E-11 |  | T | C | 0.002 | 0.007 | 0.771 | 0.000120451 | 51.418 |
| GERD | H-BMD | rs2737252 | 8 | 116663898 | A | G | 0.035 | 0.002 | 2.90001E-55 |  | A | G | -0.018 | 0.005 | 0.001 | 0.000665839 | 284.384 |
| GERD | H-BMD | rs2741856 | 17 | 41826839 | C | G | 0.075 | 0.003 | 3.90032E-89 |  | C | G | -0.007 | 0.009 | 0.397 | 0.00115675 | 494.298 |
| GERD | H-BMD | rs2761884 | 14 | 54425052 | T | G | -0.050 | 0.002 | 7.9983E-125 |  | T | G | -0.014 | 0.005 | 0.003 | 0.001679599 | 718.096 |
| GERD | H-BMD | rs2799097 | 1 | 218524632 | G | A | 0.017 | 0.003 | 4.60002E-10 |  | G | A | -0.003 | 0.007 | 0.695 | 0.000105527 | 45.046 |
| GERD | H-BMD | rs2830913 | 21 | 28774023 | A | G | 0.024 | 0.002 | 2.29985E-30 |  | A | G | -0.007 | 0.005 | 0.131 | 0.0003692 | 157.641 |
| GERD | H-BMD | rs28557305 | 9 | 98265780 | A | G | -0.026 | 0.002 | 2.80027E-34 |  | A | G | -0.006 | 0.005 | 0.247 | 0.000420184 | 179.419 |
| GERD | H-BMD | rs2884691 | 1 | 198024608 | G | A | 0.013 | 0.002 | 2.19999E-08 |  | G | A | -0.008 | 0.005 | 0.091 | 0.000114468 | 48.863 |
| GERD | H-BMD | rs2906193 | 7 | 83698177 | T | G | 0.017 | 0.002 | 3.80014E-12 |  | T | G | -0.002 | 0.006 | 0.784 | 0.00013636 | 58.210 |
| GERD | H-BMD | rs2908007 | 7 | 120962164 | G | A | 0.168 | 0.002 | 1E-200 |  | G | A | -0.008 | 0.005 | 0.122 | 0.01846664 | 8030.260 |
| GERD | H-BMD | rs293736 | 20 | 31925189 | C | A | -0.012 | 0.002 | 8.4004E-11 |  | C | A | 0.003 | 0.005 | 0.532 | 7.59716E-05 | 32.429 |
| GERD | H-BMD | rs2982573 | 6 | 152010534 | C | T | 0.077 | 0.002 | 1E-200 |  | C | T | -0.001 | 0.005 | 0.858 | 0.004002486 | 1715.214 |
| GERD | H-BMD | rs3118906 | 13 | 51106788 | A | G | 0.026 | 0.002 | 7.29962E-35 |  | A | G | 0.014 | 0.005 | 0.009 | 0.000378743 | 161.717 |
| GERD | H-BMD | rs314344 | 7 | 100395003 | C | G | -0.011 | 0.002 | 3.69999E-08 |  | C | G | -0.002 | 0.005 | 0.691 | 7.82588E-05 | 33.405 |
| GERD | H-BMD | rs330091 | 8 | 9177129 | G | A | 0.019 | 0.002 | 1.10002E-11 |  | G | A | -0.005 | 0.006 | 0.338 | 0.000160338 | 68.447 |
| GERD | H-BMD | rs34068557 | 5 | 66885830 | T | C | -0.012 | 0.002 | 2.19999E-08 |  | T | C | -0.001 | 0.005 | 0.779 | 8.82431E-05 | 37.667 |
| GERD | H-BMD | rs34123233 | 16 | 2145787 | G | T | 0.021 | 0.002 | 3.59998E-17 |  | G | T | -0.009 | 0.006 | 0.154 | 0.000183224 | 78.218 |
| GERD | H-BMD | rs34220916 | 7 | 20337532 | G | C | 0.021 | 0.002 | 2.19989E-16 |  | G | C | -0.007 | 0.006 | 0.241 | 0.000173711 | 74.157 |
| GERD | H-BMD | rs344035 | 3 | 156461279 | A | G | -0.015 | 0.002 | 8.99912E-14 |  | A | G | -0.010 | 0.005 | 0.044 | 0.000146526 | 62.550 |
| GERD | H-BMD | rs344078 | 3 | 156554568 | T | C | -0.042 | 0.002 | 3.59998E-55 |  | T | C | -0.007 | 0.006 | 0.279 | 0.000701929 | 299.809 |
| GERD | H-BMD | rs34553872 | 1 | 22702231 | T | A | 0.061 | 0.002 | 2.6977E-116 |  | T | A | -0.012 | 0.006 | 0.060 | 0.001511599 | 646.160 |
| GERD | H-BMD | rs34575960 | 13 | 94097805 | A | C | 0.026 | 0.004 | 2.59998E-08 |  | A | C | -0.004 | 0.009 | 0.696 | 0.000120309 | 51.357 |
| GERD | H-BMD | rs35264941 | 9 | 18640814 | C | G | -0.057 | 0.007 | 1.20005E-11 |  | C | G | -0.008 | 0.018 | 0.634 | 0.000155625 | 66.434 |
| GERD | H-BMD | rs35404230 | 2 | 200358968 | C | T | 0.020 | 0.003 | 1.09999E-09 |  | C | T | -0.003 | 0.007 | 0.703 | 0.00012002 | 51.234 |
| GERD | H-BMD | rs35463915 | 17 | 79392089 | T | C | -0.020 | 0.002 | 4.60045E-17 |  | T | C | 0.002 | 0.006 | 0.729 | 0.000180389 | 77.008 |
| GERD | H-BMD | rs35657711 | 2 | 42228804 | C | T | 0.051 | 0.002 | 5.5976E-102 |  | C | T | -0.001 | 0.006 | 0.822 | 0.001197306 | 511.649 |
| GERD | H-BMD | rs35713954 | 15 | 63765035 | T | G | -0.011 | 0.002 | 9.80009E-09 |  | T | G | 0.004 | 0.005 | 0.389 | 7.90813E-05 | 33.756 |
| GERD | H-BMD | rs36009197 | 6 | 130447957 | T | A | 0.019 | 0.003 | 5.89997E-09 |  | T | A | 0.014 | 0.008 | 0.075 | 0.000105053 | 44.843 |
| GERD | H-BMD | rs36010930 | 2 | 65521092 | C | T | 0.014 | 0.002 | 2.69998E-10 |  | C | T | 0.008 | 0.005 | 0.161 | 0.000106024 | 45.258 |
| GERD | H-BMD | rs368510 | 5 | 148790285 | A | G | 0.024 | 0.002 | 1.29987E-29 |  | A | G | 0.003 | 0.005 | 0.546 | 0.000341981 | 146.015 |
| GERD | H-BMD | rs370387 | 3 | 41138550 | A | G | 0.047 | 0.002 | 2.8973E-111 |  | A | G | -0.011 | 0.005 | 0.019 | 0.001492289 | 637.893 |
| GERD | H-BMD | rs3747237 | 22 | 45785840 | G | A | -0.013 | 0.002 | 1.99986E-12 |  | G | A | -0.008 | 0.005 | 0.099 | 0.000105071 | 44.851 |
| GERD | H-BMD | rs3760456 | 17 | 27948844 | T | C | -0.023 | 0.002 | 1.29987E-26 |  | T | C | 0.013 | 0.005 | 0.010 | 0.000350461 | 149.637 |
| GERD | H-BMD | rs3765971 | 1 | 8445360 | T | C | 0.028 | 0.002 | 1.9002E-37 |  | T | C | 0.005 | 0.005 | 0.337 | 0.000507452 | 216.702 |
| GERD | H-BMD | rs3777787 | 6 | 133586425 | A | C | -0.052 | 0.002 | 1.4997E-143 |  | A | C | -0.005 | 0.005 | 0.303 | 0.001870125 | 799.706 |
| GERD | H-BMD | rs3796014 | 2 | 54839276 | G | A | 0.051 | 0.002 | 4.3954E-108 |  | G | A | -0.002 | 0.005 | 0.678 | 0.001465677 | 626.501 |
| GERD | H-BMD | rs3801427 | 7 | 15721178 | T | C | 0.033 | 0.002 | 5.79963E-45 |  | T | C | -0.006 | 0.006 | 0.257 | 0.000554848 | 236.953 |
| GERD | H-BMD | rs426810 | 17 | 29958324 | G | A | 0.011 | 0.002 | 0.00000001 |  | G | A | 0.001 | 0.005 | 0.912 | 8.69133E-05 | 37.100 |
| GERD | H-BMD | rs4305309 | 2 | 54684557 | C | T | -0.073 | 0.002 | 1E-200 |  | C | T | 0.004 | 0.005 | 0.418 | 0.003426312 | 1467.453 |
| GERD | H-BMD | rs4418639 | 1 | 197359999 | A | G | -0.016 | 0.002 | 1.69981E-13 |  | A | G | 0.015 | 0.005 | 0.002 | 0.00017949 | 76.624 |
| GERD | H-BMD | rs4505759 | 4 | 1003022 | T | C | 0.057 | 0.002 | 1E-131 |  | T | C | 0.003 | 0.005 | 0.513 | 0.001875049 | 801.816 |
| GERD | H-BMD | rs4635400 | 18 | 13719510 | A | G | -0.050 | 0.002 | 1.3002E-119 |  | A | G | -0.002 | 0.005 | 0.708 | 0.001563711 | 668.472 |
| GERD | H-BMD | rs4664604 | 2 | 153573408 | G | C | 0.018 | 0.002 | 3.29989E-11 |  | G | C | 0.005 | 0.006 | 0.405 | 0.000134265 | 57.315 |
| GERD | H-BMD | rs4669522 | 2 | 10194192 | C | T | 0.017 | 0.002 | 1.89998E-10 |  | C | T | -0.003 | 0.006 | 0.653 | 0.000133838 | 57.133 |
| GERD | H-BMD | rs4693374 | 4 | 95086164 | C | A | 0.019 | 0.002 | 1.80011E-18 |  | C | A | -0.008 | 0.005 | 0.106 | 0.000241971 | 103.303 |
| GERD | H-BMD | rs4739697 | 8 | 80822461 | G | A | -0.019 | 0.002 | 3.69999E-18 |  | G | A | 0.000 | 0.005 | 0.925 | 0.000208926 | 89.193 |
| GERD | H-BMD | rs4743930 | 9 | 96454513 | T | C | 0.029 | 0.002 | 3.80014E-35 |  | T | C | 0.014 | 0.005 | 0.009 | 0.000445485 | 190.227 |
| GERD | H-BMD | rs4759843 | 12 | 131580521 | G | A | -0.012 | 0.002 | 2.99999E-08 |  | G | A | 0.001 | 0.005 | 0.846 | 8.72608E-05 | 37.248 |
| GERD | H-BMD | rs4780403 | 16 | 12060163 | A | G | -0.056 | 0.008 | 3.40001E-10 |  | A | G | 0.017 | 0.022 | 0.457 | 0.000105708 | 45.123 |
| GERD | H-BMD | rs4806003 | 19 | 34751306 | A | C | -0.012 | 0.002 | 2.30001E-08 |  | A | C | 0.007 | 0.005 | 0.192 | 8.49121E-05 | 36.245 |
| GERD | H-BMD | rs4806862 | 19 | 2523781 | T | G | 0.020 | 0.002 | 2.29985E-22 |  | T | G | -0.004 | 0.005 | 0.385 | 0.000238051 | 101.630 |
| GERD | H-BMD | rs4869284 | 5 | 95742732 | G | C | -0.014 | 0.002 | 8.60003E-11 |  | G | C | 0.004 | 0.006 | 0.529 | 9.97606E-05 | 42.584 |
| GERD | H-BMD | rs4876361 | 8 | 117566387 | A | G | -0.013 | 0.002 | 1.6E-09 |  | A | G | 0.003 | 0.005 | 0.615 | 8.69152E-05 | 37.101 |
| GERD | H-BMD | rs4936266 | 11 | 113047896 | A | G | -0.097 | 0.007 | 2.39994E-37 |  | A | G | -0.009 | 0.018 | 0.613 | 0.00046022 | 196.523 |
| GERD | H-BMD | rs4961733 | 9 | 16690612 | A | G | 0.026 | 0.002 | 5.50047E-20 |  | A | G | 0.000 | 0.006 | 0.983 | 0.000296242 | 126.480 |
| GERD | H-BMD | rs4964511 | 12 | 107360246 | C | T | -0.013 | 0.002 | 4.49997E-10 |  | C | T | -0.009 | 0.005 | 0.076 | 0.000107399 | 45.845 |
| GERD | H-BMD | rs4979905 | 10 | 79419679 | T | C | -0.026 | 0.002 | 5.60015E-24 |  | T | C | -0.008 | 0.006 | 0.218 | 0.000279145 | 119.178 |
| GERD | H-BMD | rs55704141 | 17 | 54278715 | A | G | 0.029 | 0.002 | 3.50026E-45 |  | A | G | 0.005 | 0.005 | 0.322 | 0.000541752 | 231.357 |
| GERD | H-BMD | rs55711911 | 2 | 176887842 | A | G | -0.025 | 0.004 | 2.80001E-10 |  | A | G | -0.001 | 0.010 | 0.888 | 0.000106167 | 45.319 |
| GERD | H-BMD | rs55721532 | 2 | 28952581 | G | A | 0.022 | 0.002 | 4.49987E-23 |  | G | A | 0.000 | 0.005 | 0.967 | 0.000277641 | 118.536 |
| GERD | H-BMD | rs55772556 | 2 | 28885880 | T | G | 0.014 | 0.002 | 3.10027E-11 |  | T | G | -0.004 | 0.005 | 0.476 | 0.000114237 | 48.765 |
| GERD | H-BMD | rs56104760 | 1 | 22492887 | G | A | -0.041 | 0.002 | 1.39991E-56 |  | G | A | -0.005 | 0.006 | 0.417 | 0.000725458 | 309.866 |
| GERD | H-BMD | rs56320441 | 17 | 69207164 | C | T | 0.024 | 0.003 | 5.19996E-12 |  | C | T | 0.001 | 0.009 | 0.880 | 0.000128175 | 54.715 |
| GERD | H-BMD | rs56682471 | 1 | 172253634 | T | A | 0.027 | 0.002 | 7.19946E-30 |  | T | A | -0.007 | 0.006 | 0.217 | 0.000346509 | 147.949 |
| GERD | H-BMD | rs57043009 | 17 | 59030038 | T | C | 0.053 | 0.003 | 4.90004E-71 |  | T | C | 0.005 | 0.007 | 0.455 | 0.000961478 | 410.775 |
| GERD | H-BMD | rs571356 | 7 | 83312959 | A | G | -0.016 | 0.002 | 1.39991E-12 |  | A | G | 0.002 | 0.005 | 0.657 | 0.000151751 | 64.781 |
| GERD | H-BMD | rs5735 | 16 | 23200848 | C | T | -0.018 | 0.002 | 8.10028E-16 |  | C | T | 0.010 | 0.005 | 0.060 | 0.000185974 | 79.393 |
| GERD | H-BMD | rs5754387 | 22 | 21974703 | C | G | -0.016 | 0.002 | 1.2E-09 |  | C | G | -0.009 | 0.006 | 0.154 | 0.000106053 | 45.271 |
| GERD | H-BMD | rs578789 | 6 | 44596900 | T | C | 0.020 | 0.002 | 4.40048E-16 |  | T | C | 0.003 | 0.006 | 0.599 | 0.000174036 | 74.295 |
| GERD | H-BMD | rs59243338 | 2 | 144321495 | C | T | -0.014 | 0.002 | 4.30002E-08 |  | C | T | -0.013 | 0.006 | 0.023 | 0.000102374 | 43.700 |
| GERD | H-BMD | rs59813731 | 19 | 18154576 | T | C | 0.021 | 0.002 | 2.70023E-14 |  | T | C | 0.000 | 0.006 | 0.944 | 0.00018894 | 80.659 |
| GERD | H-BMD | rs602633 | 1 | 109821511 | G | T | -0.014 | 0.002 | 2.59998E-09 |  | G | T | 0.006 | 0.006 | 0.321 | 8.71974E-05 | 37.221 |
| GERD | H-BMD | rs603424 | 10 | 102075479 | A | G | -0.030 | 0.002 | 4.79954E-27 |  | A | G | -0.002 | 0.006 | 0.719 | 0.000350721 | 149.748 |
| GERD | H-BMD | rs60507951 | 19 | 33554109 | A | G | 0.101 | 0.003 | 5.902E-168 |  | A | G | 0.010 | 0.008 | 0.233 | 0.002313576 | 989.775 |
| GERD | H-BMD | rs6054777 | 20 | 7136244 | A | G | -0.018 | 0.002 | 1.69981E-13 |  | A | G | -0.004 | 0.005 | 0.449 | 0.000165176 | 70.512 |
| GERD | H-BMD | rs6117854 | 20 | 7551554 | A | G | -0.038 | 0.002 | 5.00035E-62 |  | A | G | 0.003 | 0.005 | 0.508 | 0.000873882 | 373.318 |
| GERD | H-BMD | rs6120819 | 20 | 33653038 | C | T | 0.025 | 0.002 | 1.9002E-22 |  | C | T | 0.002 | 0.006 | 0.799 | 0.000258409 | 110.323 |
| GERD | H-BMD | rs6129493 | 20 | 38746731 | G | A | -0.014 | 0.002 | 5.30005E-09 |  | G | A | 0.007 | 0.005 | 0.203 | 0.000104929 | 44.791 |
| GERD | H-BMD | rs614802 | 11 | 128687835 | G | A | 0.015 | 0.002 | 1.79999E-10 |  | G | A | 0.003 | 0.005 | 0.577 | 0.000133418 | 56.953 |
| GERD | H-BMD | rs61733768 | 16 | 67304915 | A | G | -0.076 | 0.006 | 7.70016E-36 |  | A | G | 0.018 | 0.014 | 0.206 | 0.000443407 | 189.340 |
| GERD | H-BMD | rs61780431 | 1 | 41503820 | A | G | -0.019 | 0.002 | 2.49977E-13 |  | A | G | 0.009 | 0.006 | 0.141 | 0.000164926 | 70.406 |
| GERD | H-BMD | rs62007684 | 14 | 103909203 | C | T | -0.043 | 0.002 | 8.49963E-81 |  | C | T | -0.006 | 0.005 | 0.224 | 0.001107756 | 473.339 |
| GERD | H-BMD | rs62182131 | 2 | 218602641 | A | G | -0.027 | 0.003 | 6.4003E-13 |  | A | G | 0.011 | 0.009 | 0.210 | 0.000151451 | 64.652 |
| GERD | H-BMD | rs62302300 | 4 | 77921005 | T | C | -0.024 | 0.003 | 1.50003E-14 |  | T | C | -0.005 | 0.006 | 0.481 | 0.000217013 | 92.646 |
| GERD | H-BMD | rs634277 | 11 | 86887931 | G | A | -0.068 | 0.002 | 1E-200 |  | G | A | -0.002 | 0.005 | 0.762 | 0.002774668 | 1187.585 |
| GERD | H-BMD | rs6465495 | 7 | 95874872 | G | T | -0.020 | 0.002 | 1.20005E-21 |  | G | T | -0.009 | 0.005 | 0.091 | 0.000256588 | 109.546 |
| GERD | H-BMD | rs6469765 | 8 | 119760796 | C | T | -0.024 | 0.002 | 3.19963E-19 |  | C | T | -0.003 | 0.006 | 0.558 | 0.000247101 | 105.494 |
| GERD | H-BMD | rs6471752 | 8 | 49309167 | T | C | -0.021 | 0.003 | 5.19996E-14 |  | T | C | 0.004 | 0.007 | 0.525 | 0.000156195 | 66.678 |
| GERD | H-BMD | rs6485702 | 11 | 46898771 | C | T | -0.035 | 0.002 | 2.80027E-59 |  | C | T | 0.011 | 0.005 | 0.037 | 0.000750781 | 320.691 |
| GERD | H-BMD | rs6540965 | 1 | 11326788 | C | G | 0.013 | 0.002 | 4.49997E-08 |  | C | G | -0.007 | 0.005 | 0.182 | 0.000109455 | 46.723 |
| GERD | H-BMD | rs6542920 | 2 | 100845088 | G | A | 0.012 | 0.002 | 6.59994E-10 |  | G | A | -0.009 | 0.005 | 0.061 | 9.14581E-05 | 39.040 |
| GERD | H-BMD | rs6546334 | 2 | 68082698 | T | C | -0.018 | 0.002 | 1.39991E-16 |  | T | C | 0.003 | 0.005 | 0.612 | 0.000193431 | 82.577 |
| GERD | H-BMD | rs665783 | 2 | 45883597 | A | G | -0.019 | 0.002 | 7.50067E-18 |  | A | G | 0.005 | 0.005 | 0.390 | 0.000222822 | 95.127 |
| GERD | H-BMD | rs66927142 | 2 | 202798759 | T | C | 0.032 | 0.003 | 4.00037E-23 |  | T | C | -0.006 | 0.007 | 0.385 | 0.000292619 | 124.933 |
| GERD | H-BMD | rs6722557 | 2 | 145755449 | C | G | 0.020 | 0.002 | 4.60045E-14 |  | C | G | 0.000 | 0.006 | 0.960 | 0.000203503 | 86.877 |
| GERD | H-BMD | rs6794670 | 3 | 126208908 | G | C | -0.015 | 0.002 | 8.4004E-11 |  | G | C | 0.004 | 0.006 | 0.474 | 9.47338E-05 | 40.438 |
| GERD | H-BMD | rs6846476 | 4 | 145650021 | G | T | -0.014 | 0.002 | 6.4998E-14 |  | G | T | 0.000 | 0.005 | 0.994 | 0.000133396 | 56.944 |
| GERD | H-BMD | rs6861681 | 5 | 173362458 | A | G | 0.012 | 0.002 | 3.69999E-08 |  | A | G | -0.003 | 0.005 | 0.527 | 8.11039E-05 | 34.620 |
| GERD | H-BMD | rs6864688 | 5 | 112054086 | T | C | -0.015 | 0.002 | 2.70023E-13 |  | T | C | -0.008 | 0.005 | 0.081 | 0.000149755 | 63.928 |
| GERD | H-BMD | rs6866190 | 5 | 67102159 | G | A | -0.016 | 0.003 | 6.59994E-09 |  | G | A | -0.009 | 0.007 | 0.189 | 9.9156E-05 | 42.326 |
| GERD | H-BMD | rs6882422 | 5 | 135430668 | A | G | -0.029 | 0.003 | 1.9002E-19 |  | A | G | -0.015 | 0.008 | 0.050 | 0.000234458 | 100.095 |
| GERD | H-BMD | rs6885822 | 5 | 158263144 | A | G | -0.023 | 0.003 | 2.90001E-20 |  | A | G | 0.000 | 0.007 | 0.969 | 0.000199471 | 85.155 |
| GERD | H-BMD | rs6938070 | 6 | 83745018 | A | T | -0.024 | 0.002 | 1.69981E-24 |  | A | T | 0.005 | 0.005 | 0.384 | 0.000331127 | 141.379 |
| GERD | H-BMD | rs6965122 | 7 | 96137674 | G | A | -0.046 | 0.002 | 2.60016E-93 |  | G | A | 0.007 | 0.005 | 0.158 | 0.001294766 | 553.351 |
| GERD | H-BMD | rs6977460 | 7 | 138667788 | G | A | -0.017 | 0.002 | 5.30029E-15 |  | G | A | 0.007 | 0.005 | 0.180 | 0.000161901 | 69.114 |
| GERD | H-BMD | rs7000279 | 8 | 142299511 | T | C | 0.013 | 0.002 | 7.69999E-09 |  | T | C | -0.007 | 0.005 | 0.202 | 9.34929E-05 | 39.909 |
| GERD | H-BMD | rs7017252 | 8 | 129950844 | T | C | 0.017 | 0.002 | 6.4003E-16 |  | T | C | 0.017 | 0.005 | 0.001 | 0.000174805 | 74.624 |
| GERD | H-BMD | rs7021585 | 9 | 111108250 | A | G | 0.019 | 0.002 | 3.29989E-15 |  | A | G | -0.005 | 0.006 | 0.375 | 0.000179281 | 76.535 |
| GERD | H-BMD | rs7040344 | 9 | 133464084 | T | C | -0.028 | 0.002 | 4.40048E-39 |  | T | C | -0.002 | 0.005 | 0.699 | 0.000471514 | 201.347 |
| GERD | H-BMD | rs7078201 | 10 | 94938228 | G | T | 0.013 | 0.002 | 1.29999E-09 |  | G | T | 0.003 | 0.005 | 0.505 | 0.000109505 | 46.744 |
| GERD | H-BMD | rs7102 | 16 | 11642242 | C | T | -0.020 | 0.002 | 3.50026E-20 |  | C | T | 0.009 | 0.005 | 0.078 | 0.000261715 | 111.735 |
| GERD | H-BMD | rs7121746 | 11 | 112437007 | G | A | -0.057 | 0.002 | 3.8994E-167 |  | G | A | 0.001 | 0.005 | 0.829 | 0.002132864 | 912.299 |
| GERD | H-BMD | rs713111 | 2 | 70796339 | A | G | 0.022 | 0.003 | 4.90004E-13 |  | A | G | 0.010 | 0.007 | 0.172 | 0.000150111 | 64.080 |
| GERD | H-BMD | rs7167692 | 15 | 85660184 | C | T | -0.046 | 0.004 | 3.40017E-25 |  | C | T | 0.002 | 0.011 | 0.857 | 0.000285966 | 122.091 |
| GERD | H-BMD | rs7175531 | 15 | 51534055 | C | T | 0.032 | 0.002 | 2.39994E-52 |  | C | T | -0.006 | 0.005 | 0.268 | 0.000651776 | 278.374 |
| GERD | H-BMD | rs7176579 | 15 | 51464187 | A | G | -0.016 | 0.002 | 3.90032E-13 |  | A | G | 0.003 | 0.005 | 0.517 | 0.000163007 | 69.586 |
| GERD | H-BMD | rs7191269 | 16 | 51784918 | G | A | -0.030 | 0.002 | 1.80011E-44 |  | G | A | -0.008 | 0.005 | 0.126 | 0.000607014 | 259.244 |
| GERD | H-BMD | rs7197197 | 16 | 72914328 | A | G | 0.015 | 0.002 | 2.29985E-11 |  | A | G | -0.001 | 0.005 | 0.842 | 0.000152188 | 64.967 |
| GERD | H-BMD | rs7237942 | 18 | 20746728 | G | A | -0.017 | 0.002 | 3.19963E-12 |  | G | A | -0.018 | 0.006 | 0.003 | 0.000134246 | 57.307 |
| GERD | H-BMD | rs72692842 | 1 | 149995979 | T | C | -0.029 | 0.003 | 4.90004E-13 |  | T | C | 0.005 | 0.008 | 0.552 | 0.000194777 | 83.151 |
| GERD | H-BMD | rs72790907 | 2 | 43575984 | T | C | 0.023 | 0.003 | 2.19989E-14 |  | T | C | 0.017 | 0.007 | 0.016 | 0.000158488 | 67.657 |
| GERD | H-BMD | rs7280028 | 21 | 36416840 | C | T | 0.013 | 0.002 | 2.19999E-08 |  | C | T | -0.003 | 0.006 | 0.651 | 7.11403E-05 | 30.366 |
| GERD | H-BMD | rs72868839 | 6 | 55636651 | T | A | 0.050 | 0.004 | 8.10028E-37 |  | T | A | -0.004 | 0.009 | 0.640 | 0.000447319 | 191.011 |
| GERD | H-BMD | rs72871127 | 3 | 41531540 | C | T | -0.029 | 0.004 | 1E-12 |  | C | T | 0.011 | 0.010 | 0.276 | 0.000133504 | 56.990 |
| GERD | H-BMD | rs72945685 | 6 | 107966468 | T | C | 0.023 | 0.003 | 1.99986E-12 |  | T | C | -0.016 | 0.008 | 0.041 | 0.000148654 | 63.458 |
| GERD | H-BMD | rs72971208 | 11 | 91552059 | C | G | -0.018 | 0.003 | 2.59998E-08 |  | C | G | 0.009 | 0.008 | 0.246 | 8.87229E-05 | 37.872 |
| GERD | H-BMD | rs73207790 | 4 | 867242 | A | G | -0.023 | 0.003 | 2.19989E-17 |  | A | G | 0.001 | 0.007 | 0.874 | 0.000189298 | 80.812 |
| GERD | H-BMD | rs73238169 | 3 | 142140511 | A | G | -0.019 | 0.003 | 2.1E-10 |  | A | G | -0.012 | 0.007 | 0.079 | 0.000117652 | 50.223 |
| GERD | H-BMD | rs7329483 | 13 | 24242801 | G | A | -0.024 | 0.003 | 1.69981E-13 |  | G | A | 0.006 | 0.007 | 0.432 | 0.000163863 | 69.952 |
| GERD | H-BMD | rs73520180 | 9 | 118838314 | C | T | 0.039 | 0.003 | 1.9002E-21 |  | C | T | -0.004 | 0.009 | 0.654 | 0.000294347 | 125.671 |
| GERD | H-BMD | rs74119759 | 10 | 20150985 | T | C | -0.029 | 0.003 | 1.69981E-26 |  | T | C | 0.004 | 0.007 | 0.560 | 0.000301867 | 128.882 |
| GERD | H-BMD | rs747091 | 9 | 34045014 | G | T | 0.013 | 0.002 | 7.29962E-11 |  | G | T | 0.005 | 0.005 | 0.337 | 0.000109632 | 46.798 |
| GERD | H-BMD | rs7484147 | 11 | 27302716 | C | T | -0.047 | 0.002 | 1.7989E-108 |  | C | T | 0.016 | 0.005 | 0.002 | 0.001378787 | 589.309 |
| GERD | H-BMD | rs7504492 | 18 | 45606093 | C | T | 0.013 | 0.002 | 4.60002E-10 |  | C | T | 0.000 | 0.005 | 0.935 | 0.000106701 | 45.547 |
| GERD | H-BMD | rs75230517 | 6 | 45349509 | C | G | -0.101 | 0.004 | 3.0974E-108 |  | C | G | 0.018 | 0.011 | 0.098 | 0.001353109 | 578.319 |
| GERD | H-BMD | rs7527300 | 1 | 221477744 | T | C | -0.025 | 0.002 | 3.40017E-30 |  | T | C | -0.010 | 0.005 | 0.045 | 0.000419863 | 179.282 |
| GERD | H-BMD | rs7546500 | 1 | 26496455 | G | T | 0.015 | 0.002 | 2.49977E-12 |  | G | T | 0.009 | 0.005 | 0.079 | 0.000147516 | 62.972 |
| GERD | H-BMD | rs757179 | 7 | 27276718 | C | G | -0.038 | 0.004 | 9.09913E-18 |  | C | G | -0.010 | 0.011 | 0.323 | 0.000209389 | 89.391 |
| GERD | H-BMD | rs757286 | 12 | 48148180 | T | C | 0.012 | 0.002 | 0.000000008 |  | T | C | -0.002 | 0.005 | 0.752 | 7.81298E-05 | 33.350 |
| GERD | H-BMD | rs75729494 | 3 | 50060979 | A | T | 0.041 | 0.003 | 1.39991E-28 |  | A | T | 0.011 | 0.009 | 0.184 | 0.000351043 | 149.885 |
| GERD | H-BMD | rs75809680 | 20 | 10637057 | T | C | -0.066 | 0.003 | 3.69999E-63 |  | T | C | -0.008 | 0.009 | 0.371 | 0.000850428 | 363.290 |
| GERD | H-BMD | rs7585120 | 2 | 118847841 | T | C | 0.018 | 0.002 | 2.29985E-12 |  | T | C | -0.004 | 0.005 | 0.427 | 0.000169947 | 72.549 |
| GERD | H-BMD | rs7599234 | 2 | 41055823 | T | G | -0.012 | 0.002 | 2.90001E-08 |  | T | G | -0.001 | 0.005 | 0.915 | 9.82721E-05 | 41.949 |
| GERD | H-BMD | rs7600317 | 2 | 238353513 | A | G | -0.022 | 0.002 | 2.60016E-18 |  | A | G | 0.004 | 0.006 | 0.531 | 0.000238345 | 101.755 |
| GERD | H-BMD | rs7653133 | 3 | 119037526 | G | C | 0.010 | 0.002 | 4.30002E-09 |  | G | C | 0.005 | 0.005 | 0.358 | 6.88407E-05 | 29.385 |
| GERD | H-BMD | rs7679094 | 4 | 55899115 | T | C | -0.014 | 0.002 | 0.000000004 |  | T | C | -0.007 | 0.005 | 0.171 | 0.000122294 | 52.204 |
| GERD | H-BMD | rs76833657 | 17 | 7784295 | A | G | -0.042 | 0.003 | 1.69981E-24 |  | A | G | 0.027 | 0.009 | 0.003 | 0.000342349 | 146.172 |
| GERD | H-BMD | rs7703751 | 5 | 122831981 | T | A | -0.024 | 0.002 | 1.99986E-26 |  | T | A | -0.008 | 0.006 | 0.149 | 0.000306715 | 130.953 |
| GERD | H-BMD | rs77112003 | 12 | 124492610 | T | C | 0.014 | 0.002 | 4.70002E-10 |  | T | C | 0.014 | 0.005 | 0.007 | 0.00011476 | 48.988 |
| GERD | H-BMD | rs7728907 | 5 | 72619418 | A | G | -0.019 | 0.002 | 7.8001E-16 |  | A | G | -0.006 | 0.006 | 0.310 | 0.000184164 | 78.620 |
| GERD | H-BMD | rs7741021 | 6 | 127468274 | C | A | 0.079 | 0.002 | 1E-200 |  | C | A | 0.003 | 0.005 | 0.481 | 0.004287731 | 1837.978 |
| GERD | H-BMD | rs77847666 | 7 | 120536934 | C | T | 0.075 | 0.005 | 4.19952E-45 |  | C | T | -0.013 | 0.014 | 0.348 | 0.000567802 | 242.488 |
| GERD | H-BMD | rs7789880 | 7 | 27917094 | T | C | -0.027 | 0.002 | 5.19996E-32 |  | T | C | -0.001 | 0.005 | 0.906 | 0.000410772 | 175.399 |
| GERD | H-BMD | rs7814941 | 8 | 130720646 | G | A | 0.021 | 0.002 | 1.29987E-13 |  | G | A | 0.002 | 0.006 | 0.739 | 0.000191846 | 81.900 |
| GERD | H-BMD | rs785836 | 9 | 248749 | T | C | 0.012 | 0.002 | 2.69998E-10 |  | T | C | 0.001 | 0.005 | 0.782 | 9.45419E-05 | 40.356 |
| GERD | H-BMD | rs7866211 | 9 | 110938366 | C | T | -0.025 | 0.002 | 3.59998E-28 |  | C | T | 0.001 | 0.005 | 0.785 | 0.000344202 | 146.963 |
| GERD | H-BMD | rs7898342 | 10 | 21317504 | A | G | 0.012 | 0.002 | 2.1E-09 |  | A | G | -0.001 | 0.005 | 0.855 | 9.55266E-05 | 40.777 |
| GERD | H-BMD | rs7969486 | 12 | 26148852 | C | T | 0.012 | 0.002 | 9.69996E-10 |  | C | T | -0.001 | 0.005 | 0.817 | 9.23074E-05 | 39.402 |
| GERD | H-BMD | rs79719017 | 2 | 181924295 | C | T | 0.020 | 0.002 | 8.00018E-20 |  | C | T | -0.016 | 0.005 | 0.003 | 0.000240916 | 102.853 |
| GERD | H-BMD | rs79730878 | 17 | 43895653 | C | T | -0.024 | 0.002 | 3.90032E-22 |  | C | T | 0.003 | 0.006 | 0.574 | 0.00027181 | 116.046 |
| GERD | H-BMD | rs7980899 | 12 | 15001023 | T | C | -0.016 | 0.002 | 4.60045E-16 |  | T | C | 0.003 | 0.005 | 0.610 | 0.000166309 | 70.996 |
| GERD | H-BMD | rs8008406 | 14 | 24811159 | C | T | -0.017 | 0.002 | 8.10028E-12 |  | C | T | 0.012 | 0.005 | 0.030 | 0.00014794 | 63.153 |
| GERD | H-BMD | rs8073697 | 17 | 738853 | T | A | -0.014 | 0.002 | 3.29997E-10 |  | T | A | 0.005 | 0.005 | 0.297 | 0.000126715 | 54.092 |
| GERD | H-BMD | rs8095921 | 18 | 33152792 | A | G | 0.016 | 0.002 | 1.09999E-09 |  | A | G | -0.011 | 0.006 | 0.067 | 0.000117871 | 50.316 |
| GERD | H-BMD | rs825453 | 12 | 124508758 | T | A | -0.016 | 0.002 | 1.59993E-14 |  | T | A | -0.005 | 0.005 | 0.329 | 0.000177294 | 75.686 |
| GERD | H-BMD | rs853163 | 5 | 142553082 | A | G | 0.013 | 0.002 | 4.79999E-08 |  | A | G | -0.003 | 0.005 | 0.649 | 9.25913E-05 | 39.524 |
| GERD | H-BMD | rs868127 | 15 | 70402095 | T | C | -0.017 | 0.002 | 9.09913E-13 |  | T | C | 0.002 | 0.005 | 0.701 | 0.000151971 | 64.874 |
| GERD | H-BMD | rs884205 | 18 | 60054857 | C | A | 0.026 | 0.002 | 2.99985E-26 |  | C | A | 0.004 | 0.006 | 0.430 | 0.000340583 | 145.418 |
| GERD | H-BMD | rs890074 | 2 | 12962678 | A | G | 0.012 | 0.002 | 7.90005E-09 |  | A | G | 0.009 | 0.005 | 0.069 | 0.000106801 | 45.590 |
| GERD | H-BMD | rs899631 | 4 | 57749363 | T | G | 0.018 | 0.002 | 2.70023E-19 |  | T | G | -0.003 | 0.005 | 0.553 | 0.000219761 | 93.820 |
| GERD | H-BMD | rs901865 | 3 | 11300707 | C | T | -0.027 | 0.002 | 8.4004E-22 |  | C | T | 0.013 | 0.006 | 0.049 | 0.000283627 | 121.092 |
| GERD | H-BMD | rs912146 | 13 | 24828827 | A | G | 0.025 | 0.003 | 3.50026E-18 |  | A | G | 0.010 | 0.007 | 0.124 | 0.000230925 | 98.587 |
| GERD | H-BMD | rs928486 | 20 | 45456177 | G | A | 0.017 | 0.002 | 3.10027E-11 |  | G | A | 0.001 | 0.006 | 0.903 | 0.000137555 | 58.720 |
| GERD | H-BMD | rs9290351 | 3 | 168692352 | T | G | 0.024 | 0.003 | 5.90065E-15 |  | T | G | 0.012 | 0.007 | 0.117 | 0.000166587 | 71.115 |
| GERD | H-BMD | rs9295508 | 6 | 21392864 | G | A | 0.010 | 0.002 | 1.40001E-08 |  | G | A | -0.002 | 0.005 | 0.656 | 6.4589E-05 | 27.570 |
| GERD | H-BMD | rs932828 | 20 | 6365190 | A | G | -0.032 | 0.002 | 6.4998E-54 |  | A | G | -0.006 | 0.005 | 0.197 | 0.000669247 | 285.841 |
| GERD | H-BMD | rs9364386 | 6 | 168432093 | G | T | -0.027 | 0.002 | 2.29985E-26 |  | G | T | -0.006 | 0.006 | 0.382 | 0.000289292 | 123.512 |
| GERD | H-BMD | rs9378485 | 6 | 6961571 | C | T | -0.014 | 0.002 | 1.50003E-11 |  | C | T | 0.008 | 0.005 | 0.089 | 0.000132944 | 56.751 |
| GERD | H-BMD | rs9385629 | 6 | 133255506 | T | C | -0.013 | 0.002 | 8.30004E-10 |  | T | C | -0.008 | 0.005 | 0.115 | 9.38143E-05 | 40.046 |
| GERD | H-BMD | rs939666 | 7 | 38110394 | T | C | 0.071 | 0.002 | 2.8973E-178 |  | T | C | -0.009 | 0.006 | 0.131 | 0.002285586 | 977.773 |
| GERD | H-BMD | rs9447004 | 6 | 74458737 | G | A | -0.024 | 0.002 | 1E-30 |  | G | A | -0.002 | 0.005 | 0.754 | 0.000386365 | 164.973 |
| GERD | H-BMD | rs947091 | 10 | 31054186 | A | G | 0.040 | 0.002 | 1.50003E-87 |  | A | G | 0.014 | 0.005 | 0.004 | 0.001113368 | 475.739 |
| GERD | H-BMD | rs9513510 | 13 | 99571922 | C | G | -0.035 | 0.002 | 1.39991E-56 |  | C | G | 0.013 | 0.005 | 0.010 | 0.000714098 | 305.011 |
| GERD | H-BMD | rs9521510 | 13 | 110426871 | C | T | -0.021 | 0.002 | 8.49963E-22 |  | C | T | -0.004 | 0.005 | 0.470 | 0.000269416 | 115.024 |
| GERD | H-BMD | rs9530279 | 13 | 74677446 | C | T | -0.019 | 0.002 | 1.69981E-15 |  | C | T | 0.002 | 0.005 | 0.737 | 0.00018224 | 77.798 |
| GERD | H-BMD | rs9532858 | 13 | 42073408 | A | G | -0.015 | 0.002 | 1.10002E-13 |  | A | G | 0.004 | 0.005 | 0.409 | 0.000131615 | 56.184 |
| GERD | H-BMD | rs9557349 | 13 | 100610831 | C | T | -0.021 | 0.002 | 2.80027E-17 |  | C | T | -0.006 | 0.006 | 0.358 | 0.000201608 | 86.068 |
| GERD | H-BMD | rs9594738 | 13 | 42952145 | T | C | -0.048 | 0.002 | 1.099E-119 |  | T | C | 0.002 | 0.005 | 0.667 | 0.001546573 | 661.134 |
| GERD | H-BMD | rs960192 | 5 | 172999405 | T | C | 0.017 | 0.002 | 8.99912E-19 |  | T | C | 0.009 | 0.005 | 0.059 | 0.000197323 | 84.238 |
| GERD | H-BMD | rs9606139 | 22 | 19679303 | A | G | -0.115 | 0.003 | 1E-200 |  | A | G | 0.015 | 0.008 | 0.055 | 0.003348085 | 1433.837 |
| GERD | H-BMD | rs9631060 | 2 | 54382335 | G | A | -0.021 | 0.002 | 6.79986E-17 |  | G | A | -0.001 | 0.006 | 0.908 | 0.000200819 | 85.731 |
| GERD | H-BMD | rs9873544 | 3 | 57708022 | C | T | 0.015 | 0.002 | 7.10068E-13 |  | C | T | -0.008 | 0.005 | 0.097 | 0.000145492 | 62.108 |
| GERD | H-BMD | rs9896306 | 17 | 63279798 | A | C | -0.026 | 0.002 | 4.79954E-27 |  | A | C | 0.009 | 0.005 | 0.099 | 0.000374439 | 159.879 |
| GERD | H-BMD | rs9909232 | 17 | 76855369 | C | A | -0.015 | 0.002 | 9.20026E-11 |  | C | A | 0.003 | 0.006 | 0.661 | 8.62932E-05 | 36.835 |
| GERD | H-BMD | rs9921222 | 16 | 375782 | T | C | -0.026 | 0.002 | 2.80027E-33 |  | T | C | 0.009 | 0.005 | 0.062 | 0.000449218 | 191.822 |
| GERD | H-BMD | rs9974172 | 21 | 46362483 | G | A | 0.018 | 0.003 | 3.40001E-08 |  | G | A | 0.012 | 0.007 | 0.078 | 0.000107025 | 45.686 |
| GERD | UF-BMD | rs2707518 | 7 | 120962164 | T | G | 0.182 | 0.011 | 1.74502E-60 |  | T | G | -0.008 | 0.005 | 0.122 | 0.012136635 | 269.119 |
| GERD | UF-BMD | rs4505759 | 4 | 1003022 | T | C | 0.069 | 0.012 | 2.74701E-09 |  | T | C | 0.003 | 0.005 | 0.513 | 0.001611298 | 35.352 |
| GERD | UF-BMD | rs4869742 | 6 | 151907748 | T | C | -0.108 | 0.012 | 2.43276E-19 |  | T | C | -0.002 | 0.005 | 0.765 | 0.003677099 | 80.844 |
| GERD | UF-BMD | rs76410205 | 17 | 41826839 | T | C | 0.111 | 0.018 | 1.19594E-09 |  | T | C | -0.007 | 0.009 | 0.397 | 0.001685029 | 36.973 |
| GERD | UF-BMD | rs7741021 | 6 | 127468274 | C | A | 0.068 | 0.011 | 1.97888E-10 |  | C | A | 0.003 | 0.005 | 0.481 | 0.00184474 | 40.484 |
| FN-BMD, Femoral neck bone mineral density; H-BMD, Heel bone mineral density; LS-BMD, Lumbar spine bone mineral density; TB-BMD, Total body bone mineral density; TB-BMD-1, Total body bone mineral density (age over 60); TB-BMD-2, Total body bone mineral density (age 45-60); TB-BMD-3, Total body bone mineral density (age 30-45); TB-BMD-4, Total body bone mineral density (age 15-30); TB-BMD-5, Total body bone mineral density (age 0-15); UF-BMD, Ultra-distal forearm bone mineral density; GERD, Gastroesophageal reflux disease;R2, the proportion of risk factor variance explained by genotype; F, F statistic; Chr, Chromosome; Pos, Position; EA, Effect allele; OA, Other allele; Se, Standard error. | | | | | | | | | | | | | | | | | |
