# Supplementary material for: Gastroesophageal reflux disease and osteoporosis: A bidirectional Mendelian randomization study
Source: Medicine (Baltimore). 2025 Apr 4;104(14):e42083. doi: 10.1097/MD.0000000000042083 (PMC11977714; doi:10.1097/MD.0000000000042083)

**Supplementary Figure 1**

**MR scatter plots for the relationship of BMDs with GERD.**

(A) Causal estimates for FN-BMD on GERD; (B) Causal estimates for LS-BMD on GERD; (C) Causal estimates for H-BMD on GERD; (D) Causal estimates for UF-BMD on GERD; (E) Causal estimates for TB-BMD on GERD; (F) Causal estimates for TB-BMD-1 (age over 60) on GERD; (G) Causal estimates for TB-BMD-2 (age 45-60) on GERD; (H) Causal estimates for TB-BMD-3 (age 30-45) on GERD; (I) Causal estimates for TB-BMD-4 (age 15-30) on GERD; (J) Causal estimates for TB-BMD-5 (age 0-15) on GERD. The slope of each line corresponds to the causal estimates for each method. Individual SNP effect on the outcome (point and vertical line) against its effect on the exposure (point and horizontal line) is delineated in the background. GERD, Gastroesophageal reflux disease; TB-BMD, Total body bone mineral density; FN-BMD, Femoral neck bone mineral density; LS-BMD, Lumbar spine bone mineral density; UF-BMD, Ultra-distal forearm bone mineral density; H-BMD, Heel bone mineral density; TB-BMD-1, Total body bone mineral density (age over 60); TB-BMD-2, Total body bone mineral density (age 45-60); TB-BMD-3, Total body bone mineral density (age 30-45); TB-BMD-4, Total body bone mineral density (age 15-30); TB-BMD-5, Total body bone mineral density (age 0-15).


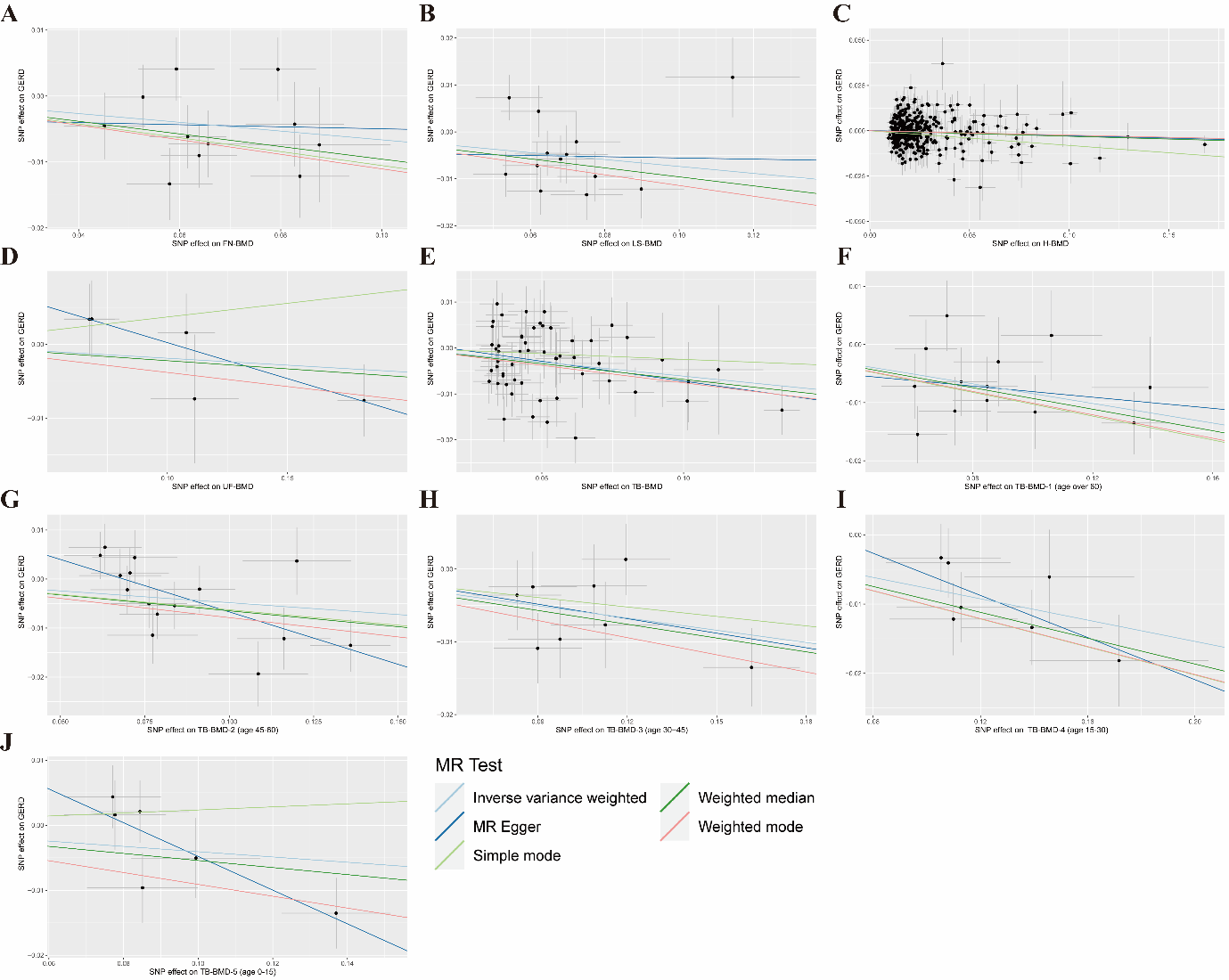


**Supplementary Figure 2**

**MR sensitivity analysis of GERD on BMD at different sites.**

Forest plot(A), leave-one-out sensitivity analysis(B) and funnel plot (C) of the effect of GERD on TB-BMD; forest plot(D), leave-one-out sensitivity analysis(E) and funnel plot (F) of the effect of GERD on FN-BMD; forest plot(G), leave-one-out sensitivity analysis(H) and funnel plot (I) of the effect of GERD on LS-BMD; forest plot(J), leave-one-out sensitivity analysis(K) and funnel plot (L) of the effect of GERD on H-BMD; forest plot(M), leave-one-out sensitivity analysis(N) and funnel plot (O) of the effect of GERD on UF-BMD. GERD, Gastroesophageal reflux disease; TB-BMD, Total body bone mineral density; FN-BMD, Femoral neck bone mineral density; LS-BMD, Lumbar spine bone mineral density; UF-BMD, Ultra-distal forearm bone mineral density; H-BMD, Heel bone mineral density.


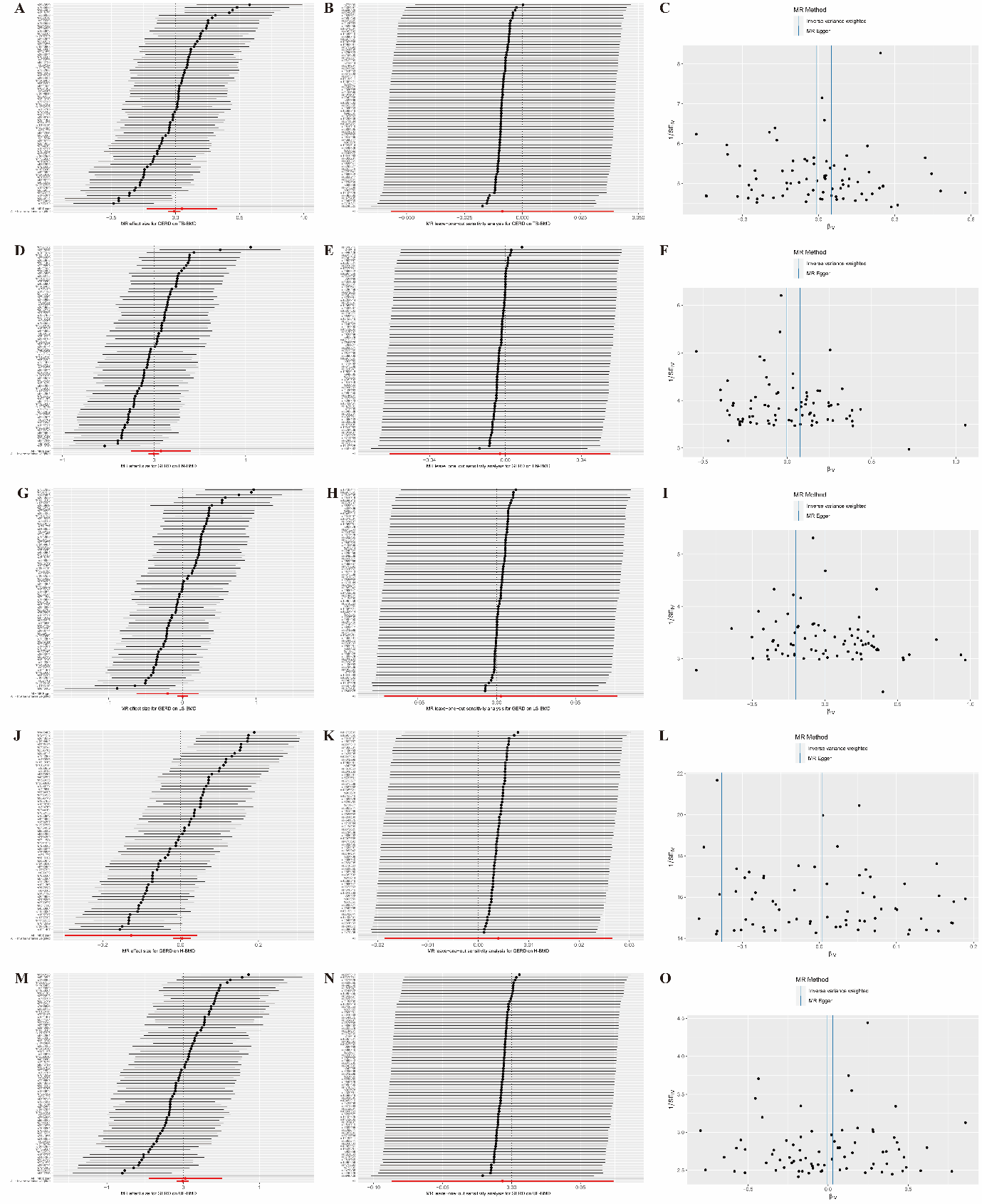


**Supplementary Figure 3**

**MR sensitivity analysis of GERD on BMD in different age groups.**

Forest plot(A), leave-one-out sensitivity analysis(B) and funnel plot (C) of the effect of GERD on TB-BMD-5; forest plot(D), leave-one-out sensitivity analysis(E) and funnel plot (F) of the effect of GERD on TB-BMD-4; forest plot(G), leave-one-out sensitivity analysis(H) and funnel plot (I) of the effect of GERD on TB-BMD-3; forest plot(J), leave-one-out sensitivity analysis(K) and funnel plot (L) of the effect of GERD on TB-BMD-2; forest plot(M), leave-one-out sensitivity analysis(N) and funnel plot (O) of the effect of GERD on TB-BMD-1. GERD, Gastroesophageal reflux disease; TB-BMD-1, Total body bone mineral density (age over 60); TB-BMD-2, Total body bone mineral density (age 45-60); TB-BMD-3, Total body bone mineral density (age 30-45); TB-BMD-4, Total body bone mineral density (age 15-30); TB-BMD-5, Total body bone mineral density (age 0-15).


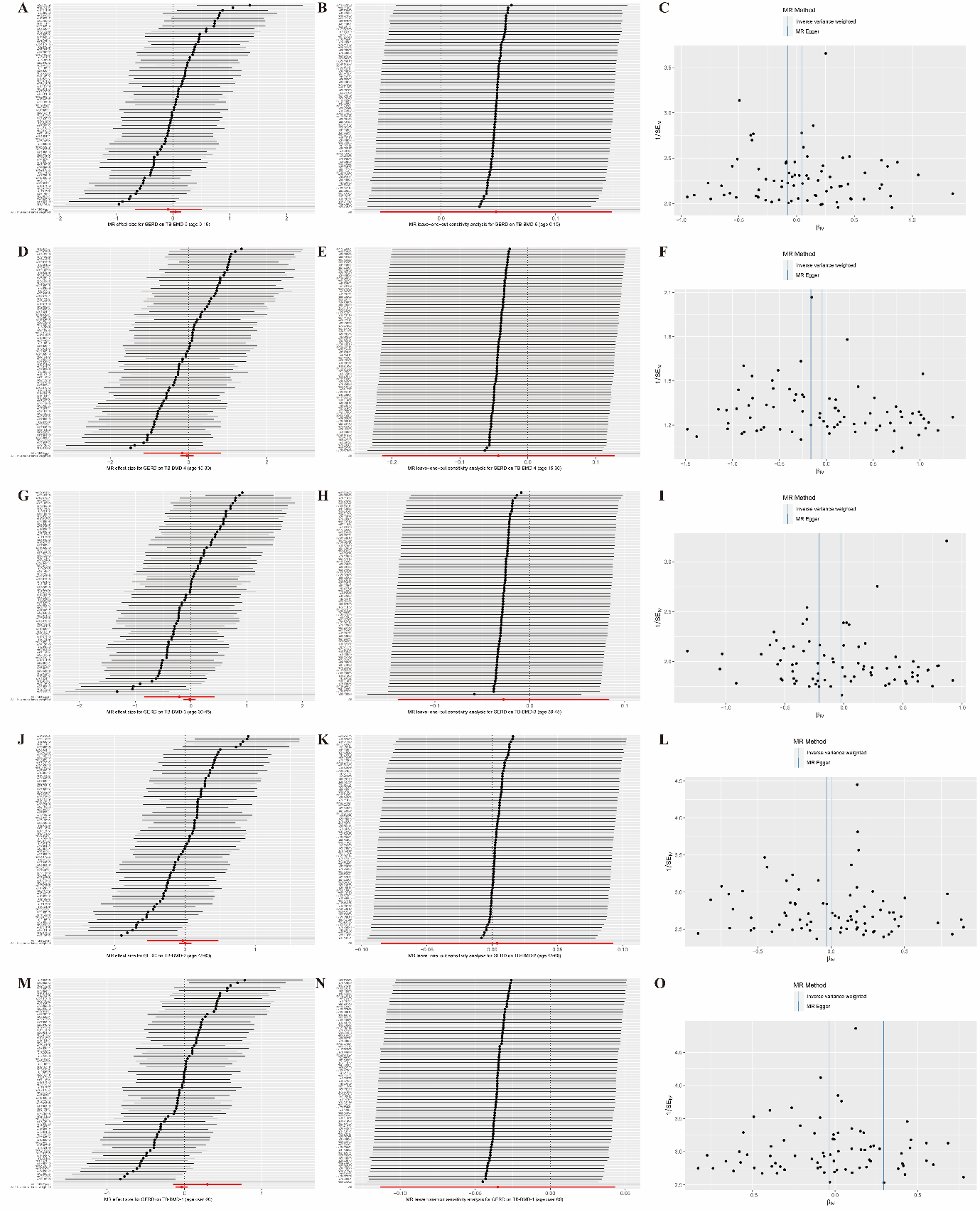


**Supplementary Figure 4**

**MR sensitivity analysis of BMD by site on GERD.**

Forest plot(A), leave-one-out sensitivity analysis(B) and funnel plot (C) of the effect of TB-BMD on GERD; forest plot(D), leave-one-out sensitivity analysis(E) and funnel plot (F) of the effect of FN-BMD on GERD; forest plot(G), leave-one-out sensitivity analysis(H) and funnel plot (I) of the effect of LS-BMD on GERD; forest plot(J), leave-one-out sensitivity analysis(K) and funnel plot (L) of the effect of H-BMD on GERD; forest plot(M), leave-one-out sensitivity analysis(N) and funnel plot (O) of the effect of UF-BMD on GERD. GERD, Gastroesophageal reflux disease; TB-BMD, Total body bone mineral density; FN-BMD, Femoral neck bone mineral density; LS-BMD, Lumbar spine bone mineral density; UF-BMD, Ultra-distal forearm bone mineral density; H-BMD, Heel bone mineral density.


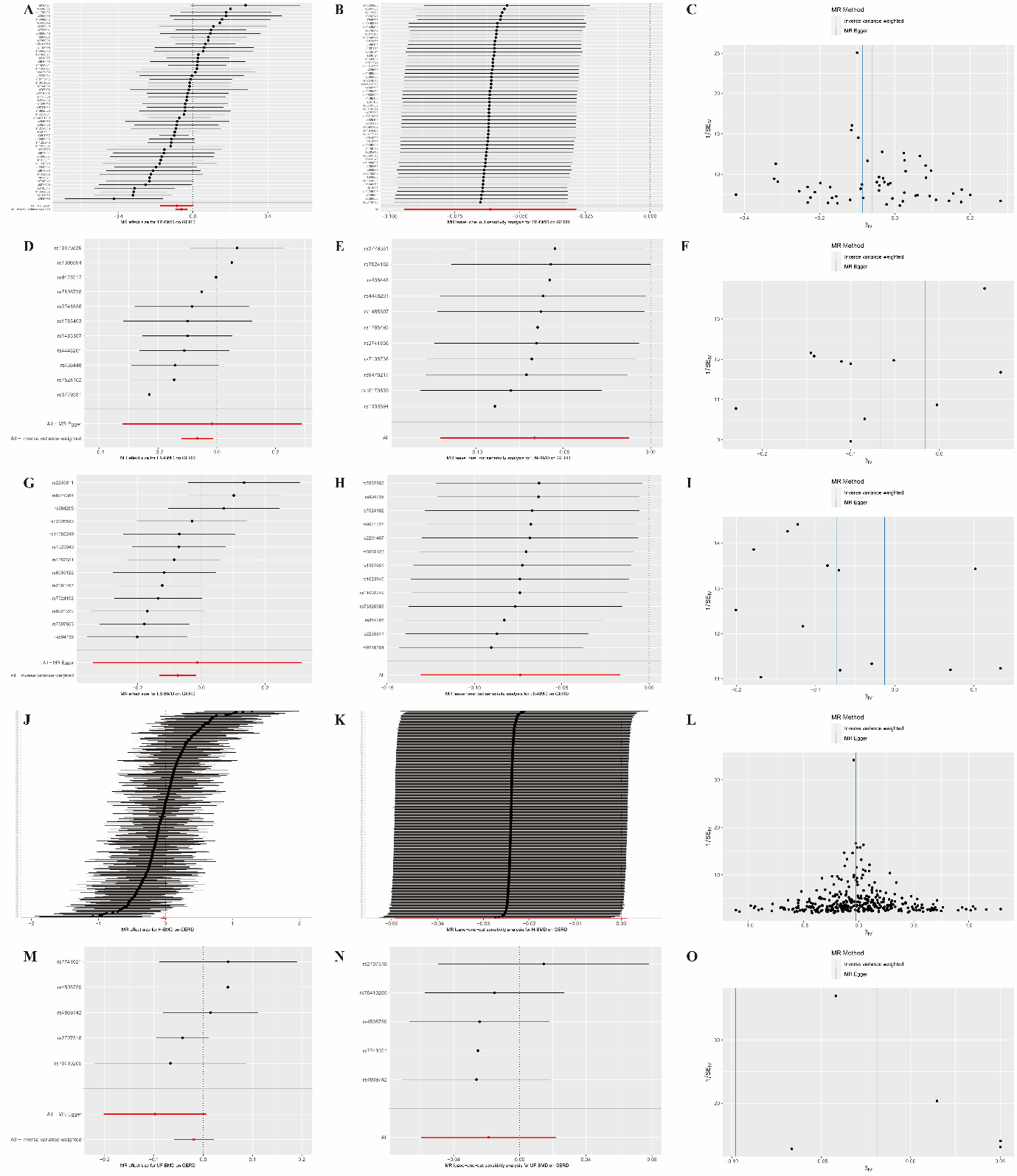


**Supplementary Figure 5**

**MR sensitivity analysis of BMD at different ages on GERD.**

Forest plot(A), leave-one-out sensitivity analysis(B) and funnel plot (C) of the effect of TB-BMD-5 on GERD; forest plot(D), leave-one-out sensitivity analysis(E) and funnel plot (F) of the effect of TB-BMD-4 on GERD; forest plot(G), leave-one-out sensitivity analysis(H) and funnel plot (I) of the effect of TB-BMD-3 on GERD; forest plot(J), leave-one-out sensitivity analysis(K) and funnel plot (L) of the effect of TB-BMD-2 on GERD; forest plot(M), leave-one-out sensitivity analysis(N) and funnel plot (O) of the effect of TB-BMD-1 on GERD. GERD, Gastroesophageal reflux disease; TB-BMD-1, Total body bone mineral density (age over 60); TB-BMD-2, Total body bone mineral density (age 45-60); TB-BMD-3, Total body bone mineral density (age 30-45); TB-BMD-4, Total body bone mineral density (age 15-30); TB-BMD-5, Total body bone mineral density (age 0-15).


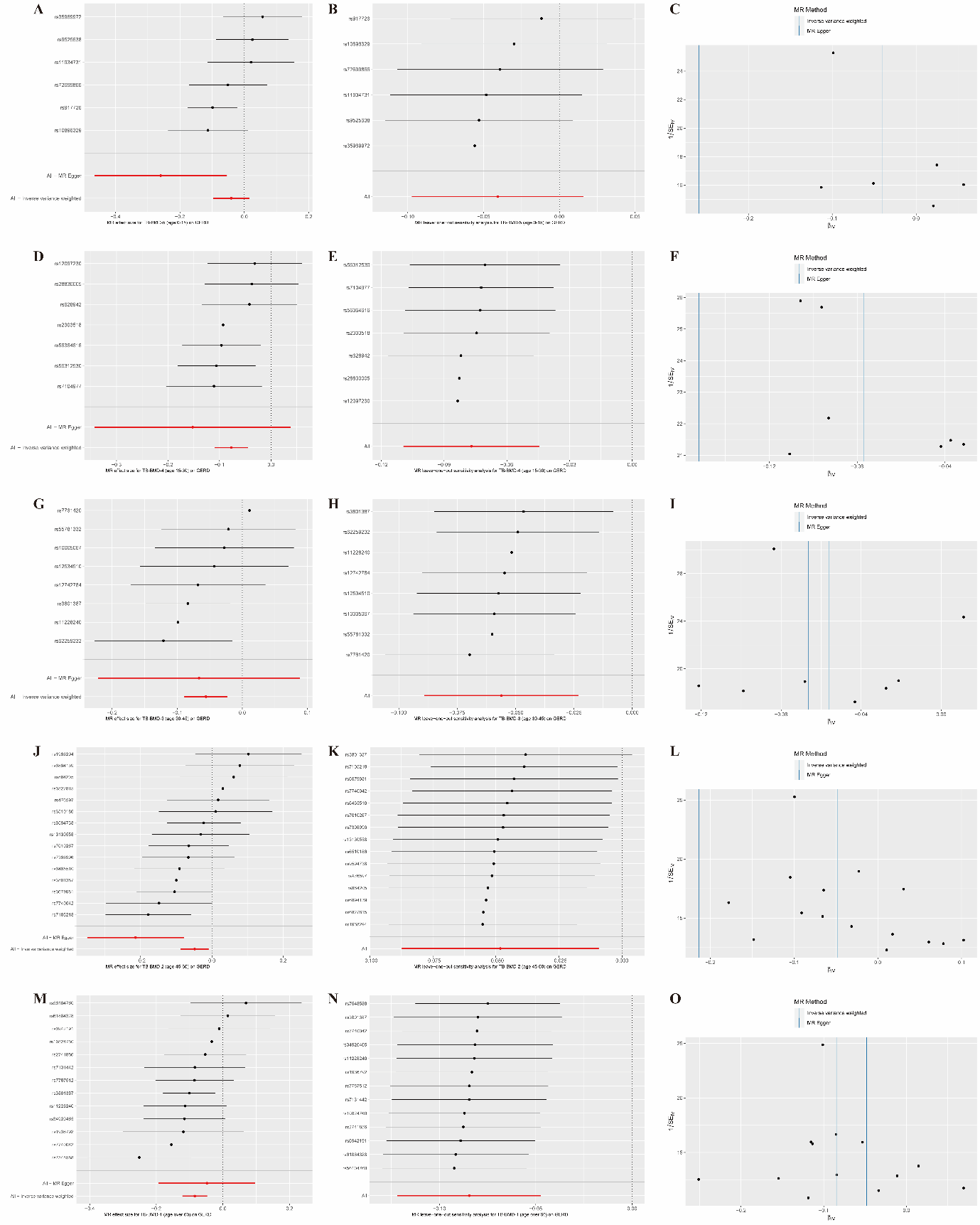

Supplement: Supplementary file 3 [file medi-104-e42083-s003.docx]
